# Global burden of polycystic ovary syndrome, uterine cancer and diabetes mellitus type 2 among women of childbearing age: trends in incidence, prevalence, mortality, and disability-adjusted life-years from 1990 to 2021

**DOI:** 10.3389/fmed.2025.1628462

**Published:** 2025-07-11

**Authors:** Ming Zhu Xie, MaoHua Qin, ZhaoJun Yang, RongXia Huang, Rong Qin

**Affiliations:** ^1^Department of Gynecology, Kunming Maternity and Child Care Hospital, Kunming, Yunnan, China; ^2^Department of Gastroenterology, Yan’an Hospital Affiliated to Kunming Medical University, Kunming, Yunnan, China

**Keywords:** polycystic ovary syndrome, uterine cancer, type 2 diabetes mellitus, global burden of disease, women of childbearing age, socioeconomic development

## Abstract

**Background:**

Polycystic ovary syndrome (PCOS), uterine cancer, and type 2 diabetes mellitus (T2DM) are major health concerns for women of childbearing age (WCBA), with complex pathogenesis and significant global burden. Understanding their epidemiological trends and risk factors is critical for public health planning.

**Methods:**

Data from the Global Burden of Disease (GBD) 2021 database were analyzed to assess the burden of PCOS, uterine cancer, and T2DM across 204 countries and territories from 1990 to 2021. Age-standardized rates of prevalence, mortality, disability-adjusted life-years (DALYs), and estimated annual percentage changes (EAPCs) were evaluated. Risk factor contributions, including high body mass index (BMI) and metabolic risks, were also examined.

**Results:**

In 2021, T2DM had the highest age-standardized incidence rate (267.85 per 100,000), followed by PCOS (64.44) and uterine cancer (2.87). From 1990 to 2021, T2DM incidence rose most significantly, while PCOS and uterine cancer also showed increasing trends. High BMI contributed to 30.76% of uterine cancer deaths and 62.87% of T2DM deaths, whereas metabolic risks accounted for 100% of T2DM deaths.

**Conclusion:**

The rising global burden of PCOS, uterine cancer, and T2DM underscores a pressing public health challenge. Prioritizing healthy lifestyles in high-burden regions and fostering international collaboration are essential to mitigate these trends.

## 1 Introduction

Polycystic ovary syndrome (PCOS), uterine cancer, and type 2 diabetes mellitus (T2DM) are significant health threats to women of reproductive age (15–49 years) worldwide. The pathogenesis of these conditions is complex. Insulin resistance, a hallmark of PCOS, not only contributes to its pathogenesis but also accelerates the development of T2DM (1). Moreover, insulin resistance induces hormonal imbalances that lead to elevated estrogen levels, stimulating excessive endometrial hyperplasia and increasing the risk of uterine cancer (2). T2DM often coexists with obesity and metabolic syndrome, and prolonged hyperglycemia contributes to the accumulation of advanced glycation end products, which activate tumor-related signaling pathways and further promote the progression of uterine cancer (3). Additionally, perimenopausal hormonal fluctuations can exacerbate ovulatory dysfunction and alter the estrogen-to-androgen ratio, significantly increasing the risk of T2DM and uterine cancer (4). Given the close interrelationship between these diseases and their substantial impact on women’s health, a comprehensive analysis of their global disease burden is crucial for the development of effective prevention and control strategies.

## 2 Materials and methods

### 2.1 Data sources

The Global Burden of Disease (GBD) 2021 study utilized anonymized data from a comprehensive database (5). This database encompasses 204 countries and territories, five socio-demographic indices (SDIs), 371 diseases, 88 risk factors, and injury-related information (6). These data were reviewed by the Institute for Health Metrics and Evaluation (IHME) at the University of Washington, United States, and access to the data was obtained using the GBD Visualization Tool (7).^1^ The findings of GBD 2021 are significant and can be useful for policymakers, public health professionals, and researchers to help identify different ways in which GBD 2021 can be used (8). The results of the GBD 2021 are significant for policymakers, public health professionals, and researchers in identifying health disparities across populations, tracking changes over time, assessing health development, and supporting the development of strategies to address health inequalities in the aftermath of the New Crown epidemic (9).

In this study, we investigated the incidence, prevalence, mortality, and disability-adjusted life years (DALYs) of polycystic ovary syndrome (PCOS), uterine cancer, and type 2 diabetes mellitus (T2DM). Polycystic ovary syndrome (PCOS) has zero reported mortality burden in the GBD study owing to its chronic non-fatal nature.

For benign diseases, data sources included hospital discharge records and claims data. Using the Bayesian meta-regression model DisMod-MR 2.1, estimates were generated based on the dimensions of age, sex, year, and country (10). For malignant cancer and type 2 diabetes, data were obtained from household surveys, census, vital statistics, and other health-related data, and cause-specific mortality rates were estimated by applying the Cause of Death Ensemble model (CDEM) (11). Disability weights (DWs) were used to capture the degree of health impairment or non-fatal disability status, and years of disability (YLDs) were calculated by multiplying the total number of cases by the number of hours of duration before remission or death multiplied by the DW. Years of premature deaths (YLLs) were derived by multiplying the number of deaths by life expectancy, determined on the basis of age, sex, geographic location, and year. DWs was calculated by multiplying the number of deaths by the number of years of life expectancy determined on the basis of age, sex, geographic location, and year. The disability-adjusted life years (DALYs) were derived by adding the number of years of pre-mature death (YLLs) to the number of years of disability (YLDs) (12).

The Socio-Demographic Index (SDI) is a composite indicator of a country’s overall level of development, considering parameters such as per capita income, average years of schooling, and young female fertility rates. The SDI ranges from 0 to 1, with the following levels: High (0.805129–1), medium (0.689504–0.805129), medium (0.607679 - 0.689504), medium-low (0.454743–0.607679), and low (0–0.454743). 0.607679– 0.689504), medium-low (0.454743–0.607679) and low (0–0.454743) (13).

### 2.2 Defnition of polycystic ovary syndrome, uterine cancer, and type 2 diabetes mellitus

In GBD 2021, polycystic ovary syndrome (PCOS), uterine cancer, and type 2 diabetes mellitus (T2DM) were identified according to the International Classification of Diseases (ICD), 9th edition of ICD-9, and 10th edition (ICD-10). Polycystic Ovary Syndrome (PCOS) (codes 256.4, E28.2), Uterine Cancer (codes 621.35, C54.1, D06.0), and type 2 Diabetes Mellitus (codes 249.00–249.11, E11.0-E11.9).

### 2.3 Attributing risk factors

In the study of risk factors in GBD 2021, exposure data were modeled using spatiotemporal Gaussian process regression or DisMod-MR 2.1. Nine quantitative relative risk estimates were generated for each set of risk-outcome pairs. These estimates were then combined with the corresponding exposure estimates to calculate the population attributable fraction (PAF) for each risk-outcome pair (14). The PAF was then multiplied by the outcome incidence rate in order to determine the number of years of attributable disability survival, years of life lost, and disability-adjusted life years. The reader is referred to previous studies for details of specific calculation procedures (15).

### 2.4 Statistical analysis

This study analyzed the impact of disease burden on polycystic ovary syndrome (PCOS), uterine cancer, and type 2 diabetes mellitus (T2DM) between 1990 and 2021. The analysis provides estimates of the burden of disease in the form of rates, age-standardized rates (ASRs) per 100,000 population, age-standardized incidence rates (ASIRs), age-standardized mortality rates (ASMRs), and age-standardized disability-adjusted life-year rates (ASDRs) (16). To assess trends over time, estimated annual percentage changes (EAPC) and their 95% confidence intervals (CIs) were calculated by linear regression modeling based on the equation Y = α + βx + ε, where Y represents the natural logarithm of the ASR, X represents the calendar year, and ε represents the error term. The EAPCs were subsequently determined based on the formula 100 × [exp (β) - 1] was determined. Statistical analyses and visualization were performed using the R software package (version 4.2.3) and JD_GBDR (V2.22, Kingdee Medical Technology Co., Ltd.) to complete the graphs (17).

## 3 Results

### 3.1 Global incidence, prevalence, mortality and DALYs

**FIGURE 1:**
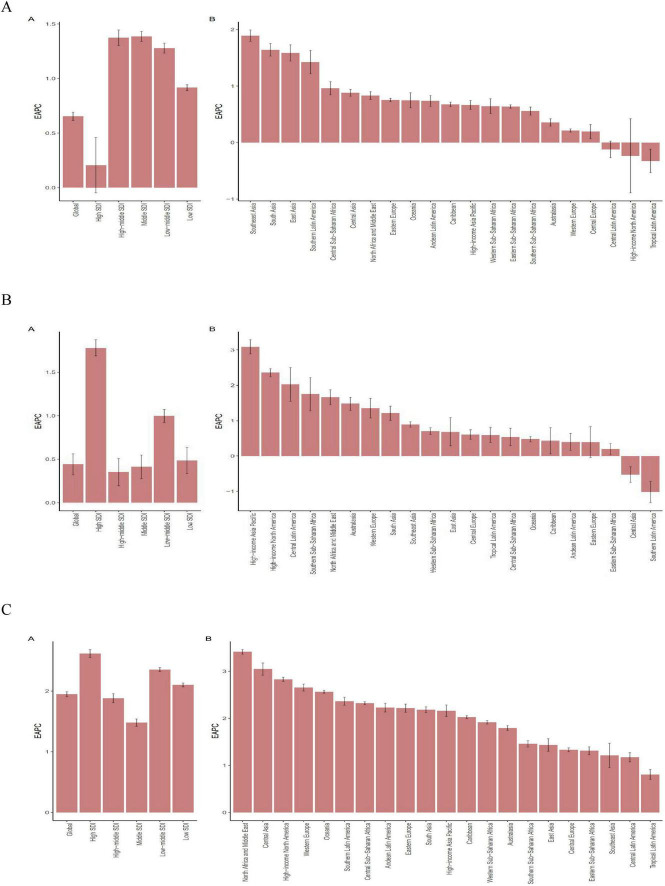
The EAPC of ASIR for PCOS, Uterine cancer, T2DM in global and 21 regions. ASIR age-standardized incidence rate, EAPC estimated annual percentage change. **(A)** PCOS Polycystic ovary syndrome. **(B)** Uterine cancer. **(C)** T2DM type 2 diabetes mellitus.

In 2021, the global incidence rate of polycystic ovary syndrome (PCOS) among women of childbearing age (WCBA) was 60.30 per 100,000 persons (95% UI 39.35–89.31), while uterine cancer had an incidence rate of 3.02 per 100,000 persons (95% UI 2.60–3.36) (Table 1; Figure 1). Diabetes mellitus type 2 had the highest global incidence rate, recorded at 272.87 per 100,000 persons (95% UI 242.83–307.43) (Table 1; Figure 1). The age-standardized incidence rate (ASIR) for type 2 diabetes mellitus was also the highest, at 267.85 per 100,000 persons (95% UI 198.90–348.61) (Table 1; Figure 1). In comparison, PCOS patients had an ASIR of 64.44 per 100,000 persons (95% UI 39.07–103.40), and uterine cancer was 2.87 per 100,000 persons (95% UI 2.44–3.23) (Table 1; Figure 1). From 1990 to 2021, the ASIR for PCOS demonstrated an upward trend, with an estimated annual percentage change (EAPC) of 0.65 persons (95% CI 0.62–0.69) (Table 1; Figure 1). Similarly, uterine cancer also showed a steady increase, with an EAPC of 0.44 (95% CI 0.32–0.56) (Table 1; Figure 1). Notably, type 2 diabetes mellitus exhibited the most significant rise, with an EAPC of 1.95 (95% CI 1.91–1.99) (Table 1; Figure 1). These findings highlight the growing burden of these conditions in recent decades (Table 1; Figure 1).

**TABLE 1:** Global incidence, prevalence, mortality, and DALYs of PCOS, Uterine cancer, T2DM from 1990 to 2021.

	PCOS	Uterine cancer	Diabetes mellitus type 2
**1990**			
Incidence (1/100,000, 95%UI)	58.84 (38.18, 87.96)	2.08 (1.73, 2.31)	132.17 (115.39, 149.92)
Prevalence (1/100,000, 95%UI)	2602.62 (1864.27, 3583.12)	16.63 (13.91, 18.31)	1575.61 (1366.49, 1809.29)
DALYs (1/100,000, 95%UI)	23.03 (10.23, 48.02)	21.82 (16.26, 25.45)	183.76 (149.67, 225.50)
Mortality (1/100,000, 95%UI)	–	0.42 (0.32, 0.49)	1.66 (1.53, 1.78)
ASIR (1/100,000, 95%UI)	52.00 (31.02, 85.27)	2.47 (2.04, 2.75)	142.14 (101.61, 192.05)
ASPR (1/100,000, 95%UI)	2628.48 (1870.21, 3668.82)	19.68 (16.44, 21.80)	1731.93 (1430.13, 2069.14)
ASMR (1/100,000, 95%UI)	–	0.60 (0.45, 0.70)	1.94 (1.76, 2.12)
ASDR (1/100,000, 95%UI)	23.16 (10.27, 48.43)	25.37 (18.86, 29.75)	207.61 (167.68, 257.59)
**2021**			
Incidence (1/100,000, 95%UI)	60.30 (39.35, 89.31)	3.02 (2.60, 3.36)	272.87 (242.83, 307.43)
Prevalence (1/100,000, 95%UI)	3374.68 (2394.97, 4649.68)	24.83 (21.44, 27.56)	3789.86 (3386.79, 4242.87)
DALYs (1/100,000, 95%UI)	29.56 (13.24, 61.66)	19.17 (15.86, 21.73)	353.83 (271.12, 451.44)
Mortality (1/100,000, 95%UI)	–	0.37 (0.31, 0.41)	2.16 (1.96, 2.36)
ASIR (1/100,000, 95%UI)	64.44 (39.07, 103.40)	2.87 (2.44, 3.23)	267.85 (198.90, 348.61)
ASPR (1/100,000, 95%UI)	3364.53 (2395.08, 4681.81)	23.59 (20.11, 26.54)	3678.58 (3154.75, 4254.46)
ASMR (1/100,000, 95%UI)	–	0.42 (0.35, 0.48)	2.06 (1.85, 2.28)
ASDR (1/100,000, 95%UI)	29.51 (13.09, 61.49)	18.29 (15.09, 20.88)	341.29 (258.87, 443.27)
**1990–2021**			
ASIR (EAPC, 95% CI)	0.65 (0.62, 0.69)	0.44 (0.32, 0.56)	1.95 (1.91, 1.99)
ASPR (EAPC, 95% CI)	0.74 (0.70, 0.77)	0.55 (0.43, 0.67)	2.43 (2.34, 2.52)
ASMR (EAPC, 95% CI)	–	–1.34 (–1.45, –1.23)	0.01 (–0.11, 0.13)
ASDR (EAPC, 95% CI)	0.72 (0.68, 0.76)	–1.24 (–1.34, –1.13)	1.51 (1.44, 1.58)

PCOS, polycystic ovary syndrome; T2DM, type 2 diabetes mellitus; DALYs, disability-adjusted life-years; ASIR, age-standardized incidence rate; ASPR, age-standardized prevalence rate; ASMR, age-standardized mortality rate; ASDR, age-standardized DALYs rate; EAPC, estimated annual percentage change; CI, confdence interval; UI, uncertainty intervals.

The global prevalence rate of polycystic ovary syndrome (PCOS) related to WCBA in 2021 is 3,374.68 per 100,000 persons (95% UI, 2,394.97–4,649.68), while uterine cancer had a prevalence of 24.83 per 100,000 persons (95% UI 21.44–27.56) (Table 1; Supplementary Table S1; Supplementary Figure S1). Type 2 diabetes had the highest prevalence, reaching 3,789.86 per 100,000 persons (95% UI 3,386.79–4,242.87). In terms of age-standardized prevalence rates (ASPR), type 2 diabetes also ranked highest at 3,678.58/100,000 persons (95% UI 3,154.75–4,254.46), followed by PCOS at 3,364.53 per 100,000 persons (95% UI, 2,395.08–4,681.81), and uterine cancer at 23.59 per 100,000 persons (95% UI 20.11–26.54) (Table 1; Supplementary Table S1; Supplementary Figure S1). From 1990 to 2021, the ASPR trends for these conditions showed notable differences. The ASPR for PCOS steadily increased, with an estimated annual percentage change (EAPC) of 0.74 (95% CI 0.70–0.77) (Table 1; Supplementary Table S1; Supplementary Figure S1). Uterine cancer also demonstrated an upward trend, with an EAPC of 0.55 (95% CI 0.43–0.67) (Table 1; Supplementary Table S1; Supplementary Figure S1). Type 2 diabetes exhibited the most significant increase, with an EAPC of 2.43 (95% CI 2.34–2.52) (Table 1; Supplementary Table S1; Supplementary Figure S1). These trends highlight the increasing burden of these diseases in the past few decades.

The global disability-adjusted life years (DALYs) for polycystic ovary syndrome (PCOS) are 29.56 per 100,000 persons (95% UI 13.24–61.66) in 2021, while uterine cancer contributed 19.17 per 100,000 persons (95% UI 15.86–21.73) (Table 1; Supplementary Table S2; Supplementary Figure S2). Type 2 diabetes had the highest DALYs, reported as 353.83 per 100,000 individuals (95% UI 271.12–451.44) (Table 1; Supplementary Table S2; Supplementary Figure S2). For age-standardized DALYs (ASDR), type 2 diabetes was also the highest at 341.29 per 100,000 persons (95% UI 258.87–443.27), followed by PCOS at 29.51 per 100,000 persons (95% UI 13.09–61.49) and uterine cancer at 18.29 per 100,000 persons (95% UI 15.09–20.88) (Table 1; Supplementary Table S2; Supplementary Figure S2). From 1990 to 2021, the trends in ASDR showed distinct patterns.

The ASDR for PCOS increased consistently, with an estimated annual percentage change (EAPC) of 0.72 (95% CI 0.68–0.76). In contrast, uterine cancer showed a declining trend, with an EAPC of –1.24 (95% CI –1.34 to –1.13) (Table 1; Supplementary Table S2; Supplementary Figure S2). Type 2 diabetes demonstrated the most significant upward trend, with an EAPC of 1.51 (95% CI 1.44–1.58) (Table 1; Supplementary Table S2; Supplementary Figure S2). These results illustrate the varying trajectories of the burden of these diseases in the past few decades.

Globally, the burden of PCOS-related deaths was reported to be 0 in the 2021 GBD study, reflecting its chronic and non-fatal nature. The mortality and age-standardized mortality rates (ASMRs) for uterine cancer and type 2 diabetes showed distinct patterns. The global mortality rate for uterine cancer is 0.37 per 100,000 persons (95% UI 0.31–0.41), whereas type 2 diabetes has a significantly higher mortality rate of 2.16 per 100,000 persons (95% UI 1.96–2.36) (Table 1; Supplementary Table 3; Supplementary Figure S3). The ASMR for uterine cancer was 0.42 per 100,000 persons (95% UI 0.35–0.48), compared to 2.06 per 100,000 persons (95% UI 1.85–2.28) for type 2 diabetes (Table 1; Supplementary Table 3; Supplementary Figure S3). Between 1990 and 2021, the trends in ASMR revealed notable differences. Uterine cancer showed a significant decline, with an estimated annual percentage change (EAPC) of –1.34 (95% CI –1.45 to –1.23) (Table 1; Supplementary Table 3; Supplementary Figure S3). In contrast, type 2 diabetes exhibited a slight upward trend, with an EAPC of 0.01 (95% CI –0.11 to 0.13). These trends have underscored the varying mortality dynamics of these conditions in recent decades (Table 1; Supplementary Table 3; Supplementary Figure S3).

### 3.2 Regional incidence, prevalence, mortality, and DALYs

In 2021, the highest absolute rates of ASIR, ASPR, and ASDR of PCOS in WCBA were observed in the High SDI, with 165.70 per 100,000 persons (95% UI 99.24,261.46), 6825.02 per 100,000 persons (95% UI 5014.42,9336.43), 60.32 per 100,000 persons (95% UI 27.37,122.77), respectively (Table 2; Figure 1; Supplementary Table S1). From 1990 to 2021, the High SDI region demonstrated steady stagnation in ASIR, ASPR, and ASDR rates, with an EAPC of 0.21 (95% CI –0.05,0.46), 0.10 (95% CI –0.08,0.27), 0.08 (95% CI –0.09, 0.25), respectively (Table 2; Figure 1; Supplementary Tables 1–3; Supplementary Figures S1–S3). At the GBD regional level, the High-income Asia Pacific region recorded the highest incidence, prevalence, and DALYs for PCOS, with rates of 197.24 per 100,000 persons (95% UI 112.76–306.01), 10,239.02 per 100,000 persons (95% UI 7,234.28–14,296.03), and 88.65 per 100,000 persons (95% UI 39.90–179.93), respectively (Table 2; Figure 1; Supplementary Tables 1–3; Supplementary Figures S1–S3). The region also had the highest age-standardized rates, including ASIR at 308.16/100,000 persons (95% UI 171.53–485.83), ASPR at 10,116.87 per 100,000 persons (95% UI 7,086.92–14,260.97), and ASDR at 88.17/100,000 persons (95% UI 39.21–180.74). In terms of trends, South Asia showed the highest EAPC for ASIR, ASPR, and ASDR, with values of 1.89 (95% CI 1.79–1.99), 2.30 (95% CI 2.19–2.40), and 2.26 (95% CI 2.16–2.36), respectively (Figure 1; Supplementary Table 4; Supplementary Figures S1–S3).

**TABLE 2:** Regional incidence and ASIR of PCOS, Uterine cancer, T2DM in 2021.

	PCOS		Uterine cancer		Diabetes mellitus type 2	
Location	Incidence (1/100,000, 95%UI)	ASIR (1/100,000, 95%UI)	Incidence (1/100,000, 95%UI)	ASIR (1/100,000, 95%UI)	Incidence (1/100,000, 95%UI)	ASIR (1/100,000, 95%UI)
Global	60.30 (39.35, 89.31)	64.44 (39.07, 103.40)	3.02 (2.60, 3.36)	2.87 (2.44, 3.23)	272.87 (242.83, 307.43)	267.85 (198.90, 348.61)
High SDI	119.34 (73.60, 183.49)	165.70 (99.24, 261.46)	6.37 (6.11, 6.64)	5.18 (4.91, 5.45)	294.61 (262.51, 329.87)	269.46 (203.17, 346.03)
High-middle SDI	47.41 (31.31, 70.22)	68.27 (42.10, 110.41)	5.99 (5.15, 6.94)	4.71 (3.98, 5.58)	261.94 (228.60, 300.14)	249.78 (179.81, 331.74)
Middle SDI	61.12 (40.64, 89.97)	71.14 (43.76, 113.70)	2.70 (2.03, 3.22)	2.44 (1.83, 2.95)	291.49 (258.98, 330.85)	280.79 (207.35, 365.97)
Low-middle SDI	49.54 (32.64, 73.09)	46.65 (27.76, 76.97)	1.24 (1.02, 1.48)	1.35 (1.09, 1.66)	285.16 (251.83, 321.09)	295.35 (218.74, 382.40)
Low SDI	40.34 (26.56, 59.25)	31.35 (18.29, 52.50)	0.73 (0.55, 0.96)	0.94 (0.70, 1.27)	200.58 (177.12, 225.50)	219.17 (159.14, 287.26)
Andean Latin America	88.49 (60.59, 134.57)	95.75 (62.97, 152.74)	3.39 (2.51, 4.49)	3.45 (2.40, 4.88)	199.60 (176.10, 223.68)	201.01 (151.54, 254.25)
Australasia	160.50 (96.32, 261.57)	219.55 (128.92, 364.51)	4.26 (3.63, 5.00)	3.59 (2.82, 4.52)	142.99 (120.25, 170.03)	129.50 (88.90, 176.37)
Caribbean	44.67 (30.09, 66.11)	48.07 (29.89, 77.78)	6.15 (5.07, 7.41)	5.93 (4.72, 7.32)	510.51 (456.90, 570.99)	501.46 (374.41, 637.34)
Central Asia	16.43 (10.75, 23.75)	19.11 (11.02, 32.34)	3.61 (3.03, 4.26)	3.49 (2.90, 4.14)	275.90 (247.67, 305.61)	268.04 (201.29, 341.53)
Central Europe	6.22 (4.25, 8.75)	8.76 (5.19, 14.72)	7.37 (6.52, 8.34)	5.44 (4.74, 6.20)	232.81 (200.24, 272.07)	184.61 (138.80, 237.25)
Central Latin America	75.35 (53.81, 106.59)	79.38 (54.19, 118.96)	2.50 (2.13, 2.88)	2.42 (2.04, 2.83)	449.12 (402.34, 500.34)	443.21 (331.63, 569.07)
Central Sub-Saharan Africa	37.43 (24.27, 56.30)	28.80 (16.68, 48.90)	0.70 (0.44, 1.04)	0.93 (0.53, 1.52)	191.18 (165.51, 219.22)	204.85 (145.46, 270.45)
East Asia	40.96 (26.79, 60.66)	60.34 (35.86, 100.15)	4.56 (3.20, 6.38)	3.57 (2.44, 4.99)	267.05 (232.48, 309.65)	277.88 (195.01, 374.77)
Eastern Europe	10.37 (7.42, 14.27)	14.20 (7.83, 24.39)	12.97 (11.45, 14.59)	9.73 (8.52, 11.06)	200.63 (169.52, 235.61)	168.59 (118.56, 227.04)
Eastern Sub-Saharan Africa	38.93 (25.40, 58.05)	29.75 (17.07, 50.50)	0.77 (0.53, 1.16)	1.03 (0.69, 1.59)	97.34 (84.59, 111.82)	104.58 (73.61, 140.13)
High-income Asia Pacific	197.24 (112.76, 306.01)	308.16 (171.53, 485.83)	6.29 (5.69, 6.99)	4.48 (3.89, 5.16)	297.48 (256.91, 343.55)	264.31 (190.73, 352.27)
High-income North America	156.71 (93.55, 241.36)	188.01 (110.12, 296.07)	8.88 (8.48, 9.28)	7.79 (7.35, 8.27)	342.84 (304.16, 383.31)	315.71 (238.45, 403.53)
North Africa and Middle East	68.06 (44.58, 104.76)	70.52 (43.90, 115.37)	2.08 (1.56, 2.50)	2.08 (1.52, 2.57)	462.22 (413.77, 519.07)	461.78 (353.64, 578.99)
Oceania	69.86 (45.63, 107.30)	65.58 (40.69, 106.64)	2.71 (1.45, 4.64)	3.05 (1.57, 5.36)	635.19 (568.76, 709.53)	659.24 (492.70, 833.65)
South Asia	44.64 (29.91, 63.62)	43.35 (25.60, 71.42)	0.82 (0.65, 1.12)	0.88 (0.68, 1.22)	291.41 (252.69, 332.19)	297.48 (213.09, 394.38)
Southeast Asia	101.03 (63.58, 155.99)	111.59 (67.37, 181.50)	3.11 (2.05, 3.91)	2.92 (1.82, 3.76)	220.95 (194.01, 251.03)	212.94 (159.06, 272.34)
Southern Latin America	72.04 (45.12, 113.15)	84.61 (49.37, 139.92)	1.95 (1.72, 2.23)	1.81 (1.45, 2.26)	242.52 (207.37, 276.07)	229.21 (167.17, 295.96)
Southern Sub-Saharan Africa	43.75 (28.59, 64.65)	44.21 (25.89, 74.23)	1.40 (1.10, 1.80)	1.49 (1.13, 1.98)	190.90 (162.63, 221.71)	196.78 (136.45, 267.62)
Tropical Latin America	20.18 (14.06, 28.85)	24.04 (14.51, 40.50)	2.15 (2.01, 2.30)	1.95 (1.76, 2.14)	250.93 (212.27, 293.68)	233.39 (166.31, 314.15)
Western Europe	90.62 (60.99, 137.76)	122.55 (80.73, 192.67)	5.18 (4.91, 5.48)	4.00 (3.69, 4.34)	211.65 (182.44, 242.80)	206.22 (144.18, 276.37)
Western Sub-Saharan Africa	41.10 (26.92, 60.52)	31.28 (17.97, 53.14)	0.45 (0.33, 0.63)	0.61 (0.43, 0.85)	161.23 (141.66, 182.82)	175.76 (125.21, 235.84)

ASIR, age-standardized incidence rate; UI, uncertainty intervals; PCOS, Polycystic ovary syndrome; T2DM, type 2 diabetes mellitus.

Uterine cancer of high SDI regions showing the highest rates of ASIR and ASPR with 5.18 per 100,000 persons (95% UI 4.91–5.45),43.91 per 100,000 persons (95% UI 41.61–46.23), in 2021 (Table 2; Figure 1; Supplementary Tables 1–3; Supplementary Figures S1–S3). Furthermore, from 1990 to 2021, the ASIR, ASPR, ASDR, and ASMR rates in WCBA with EAPC of Uterine cancer demonstrated a rapid increase, with 1.78 (95% CI 1.69–1.87),1.83 (95% CI 1.73–1.92), 0.40 (95% CI 0.30–0.51), 0.20 (95% CI 0.09–0.32) (Figure 1; Supplementary Table 4; Supplementary Figures S1–S3). At the regional level of GBD, Uterine cancer had the highest incidence and prevalence in Eastern Europe, with rates of 12.97 per 100,000 persons (95% UI 11.45–14.59) and 108.32 per 100,000 persons (95% UI 95.72–122.05) (Table 2; Figure 1; Supplementary Tables S1–S3; Supplementary Figures S1–S3). The region also had the highest age-standardized rates, with an ASIR of 9.73 per 100,000 persons (95% UI 8.52–11.06) and an ASPR of 81.37 per 100,000 persons (95% UI 71.28–92.44) (Table 2; Figure 1; Supplementary Tables 1–S3; Supplementary Figures S1–S3). In terms of DALYs, the Caribbean region in 2021 showed the highest burden, at 64.43 per 100,000 persons (95% UI 51.62–80.77). The Caribbean also had the highest ASDR, reported at 62.39 per 100,000 persons (95% UI 47.26–81.73), along with the highest mortality rate, at 1.27 per 100,000 persons (95% UI 1.01–1.61), and the highest ASMR, at 1.47 per 100,000 persons (95% UI, 1.12–1.93) (Table 2; Figure 1; Supplementary Tables 1–S3; Supplementary Figures S1–S3). Regarding the EAPC of uterine cancer, the high-income Asia-Pacific region recorded the highest ASIR and ASPR increases in 2021, with values of 3.09 (95% CI 2.89–3.29) and 3.16 (95% CI 2.97–3.36). The highest EAPC values for ASDR and ASMR were observed in the high-income North America region, at 1.69 (95% CI 1.54–1.85) and 1.55 (95% CI 1.39–1.71) (Figure 1; Supplementary Table S4; Supplementary Figures S1–S3).

Type 2 diabetes mellitus had the highest ASIR, ASPR and ASDR in 2021 of 295.35 per 100,000 persons (95% UI 218.74–382.40), 3857.22 per 100,000 persons (95% UI 3263.74,–4504.79) and 412.23 per 100,000 persons (95% UI 320.20–526.10) in Low-middle SDI (Table 2, Figure 1; Supplementary Tables 1–S3, Supplementary Figures S1–S3). The highest ASMR for Low SDI was 3.41 per 100 000 persons (95% UI 2.82, 4.02) (Table 2; Figure 1; Supplementary Tables 1–S3, Supplementary Figures S1–S3). High SDI had the highest upward trend in ASIR, ASPR, and ASDR EAPC with 2.62 (95% CI 2.55–2.69), 3.39 (95% CI 3.32–3.46), and 2.21 (95% CI 2.12–2.31), respectively. In the regional level of GBD, Oceania had the highest rates for type 2 diabetes in 2021, including incidence, prevalence, DALYs, and mortality. The incidence rate was 635.19 per 100,000 persons (95% UI 568.76–709.53), while the prevalence reached 8,303.13 per 100,000 persons (95% UI 7,419.92–9,288.47) (Table 2; Figure 1; Supplementary Tables 1–S3; Supplementary Figures S1–S3). DALYs were reported at 1,225.86 per 100,000 persons (95% UI 995.69–1,548.32), and the mortality rate was 13.82 per 100,000 persons (95% UI 10.85–17.44) (Table 2; Figure 1; Supplementary Tables 1–S3; Supplementary Figures S1–S3).

Oceania also had the highest age-standardized rates in 2021, with an ASIR of 659.24 per 100,000 persons (95% UI 492.70–833.65), an ASPR of 8,802.82 per 100,000 persons (95% UI 7,547.50–10,199.76), an ASDR of 1,334.68 per 100,000 persons (95% UI 1,039.92–1,700.89), and an ASMR of 15.39 per 100,000 persons (95% UI 11.50–20.33) (Table 2; Figure 1; Supplementary Tables 1–S3; Supplementary Figures S1–S3). For trends in EAPC, North Africa and the Middle East had the highest ASIR EAPC at 3.41 (95% CI 3.36–3.46). High-income North America recorded the highest ASPR EAPC at 3.90 (95% CI 3.79–4.02). Western Europe had the highest ASDR EAPC, 2.32 (95% CI 2.22–2.42), while Southern Sub-Saharan Africa reported the highest ASMR EAPC, reaching 1.54 (95% CI 0.90–2.19) (Figure 1; Supplementary Table 4, Supplementary Figures S1–S3).

National incidence, prevalence, mortality, and DALYs

In the WCBA, in 2021, Japan had the highest ASIR of polycystic ovary syndrome (PCOS) at 360.92 per 100,000 (95% UI 199.08–573.59) (Figure 3). Italy had the highest ASPR with 15,307.74 per 100,000 (95% UI 10,698.08–21,343.59) (Supplementary Table 5). Furthermore, Italy had the highest SADR at 135.48 per 100,000 (95% UI 59.84–288.85). In terms of EAPC, Maldives had the fastest improvement in ASIR EAPC at 2.86 (95% CI 2.61–3.11) (Supplementary Table 5). Maldives also showed the fastest improvement in SAPR EAPC at 3.39 (95% CI 3.10–3.67). Maldives had the fastest advancing SADR EAPC at 3.39 (95% CI 3.11–3.68) (Supplementary Table S5).

**FIGURE 2:**
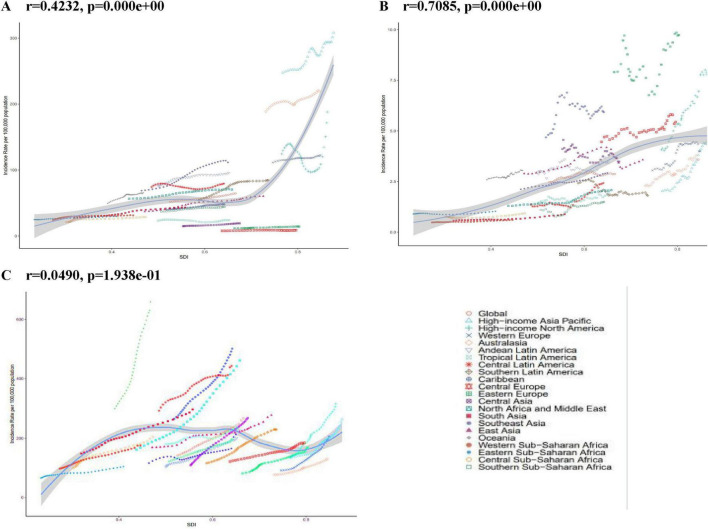
ASIR for PCOS, Uterine cancer, T2DM in global and 21 regions. From 1990 to 2021. ASIR age-standardized incidence rate. **(A)** PCOS Polycystic ovary syndrome. **(B)** Uterine cancer. **(C)** T2DM type 2 diabetes mellitus.

**FIGURE 3:**
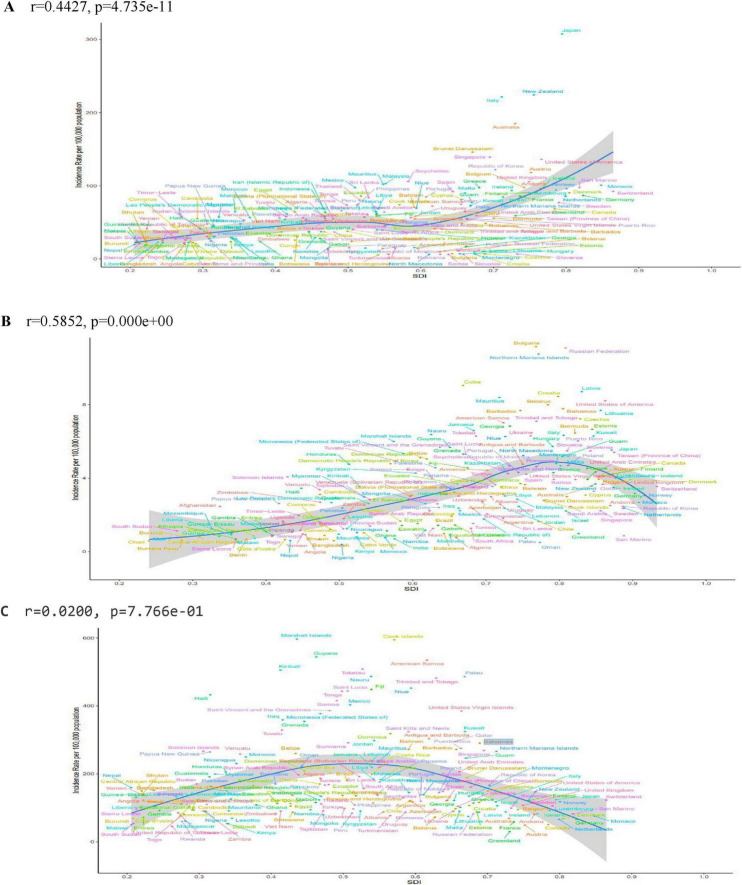
ASIR for PCOS, uterine cancer, T2DM in 204 countries and territories by SDI in 2021. ASIR age standardized incidence rate, SDI sociodemographic index. **(A)** PCOS Polycystic ovary syndrome. **(B)** Uterine cancer. **(C)** T2DM type 2 diabetes mellitus.

The ASIR for uterine cancer, In the WCBA, was highest in Bulgaria at 11.16 per 100,000 persons (95% UI 8.24–14.63) (Supplementary Table S6). Conversely, the Russian Federation demonstrated the highest SAPR, with a value of 93.12 per 100,000 persons (95% UI 80.50–105.58) (Supplementary Table S6). Guyana demonstrated the highest SADR and SAMR, with values of 84.26 per 100,000 persons (95% UI 53.66–124.24) and 2.01 per 100,000 persons (95% UI 1.28–2.95), respectively. From to 1990 to 2021, the SAIR rate and SAPR rate, with EAPC rising fastest in Italy at 4.66 (95% CI 3.57–5.77), 4.71 (95% CI 3.61–5.83) (Supplementary Table S6). Zimbabwe had the fastest rising SAIR and SAPR rates, with an EAPC of 4.13 (95% CI 3.00–5.28), 4.13 (95% CI 3.00–5.27), respectively (Supplementary Table S6).

In type 2 diabetes among the WCBA, the country with the highest value of the SAIR indicator was the Marshall Islands, with a value of 1374.15 per 100,000 persons (95% UI 969.34–1825.18) (Supplementary Table S7). The country with the highest value of EAPCs was Egypt, with 4.66 (95% CI 4.57–4.76) (Supplementary Table S7). The country to which the highest value of the SAPR indicator corresponds is the Marshall Islands with a value of 19293.07 per 100,000 persons (95% UI 16264.74– 22925.23), and its highest value of EAPCs is from Greenland with 7.84 (9 5% CI 7.70–7.98) (Supplementary Table S7). The SADR is indicated by the highest value, which corresponds to the Marshall Islands, with a value of 3370.49 per 100,000 persons (95% UI 2301.71–4684.49), and its highest EAPCs are from Lesotho, at 4.77 (95% CI 4.11–5.42) (Supplementary Table S7). The highest SAMR indicator belonged to the Marshall Islands, with a value of 43.53 per 100,000 persons (95% UI 23.94–68.31) and the highest EAPCs from Lesotho at 5.78 (95% CI 4.72–6.85) (Supplementary Table S7). Burden of three diseases by age.

Among the three diseases, ASIR and type 2 diabetes showed the most significant increase in incidence, especially in the 45–49 age group. The incidence of PCOS increased in the 15–19 age group, with little change in the remaining age groups. The incidence of uterine cancer increased relatively less but still showed an upward trend, especially in the 45–49 age group. In these three diseases, the ASPR, ASDR, and DALYs showed different changes. ASMR for uterine cancer and type 2 diabetes also differed with respect to age (Supplementary Figures S14–S17).

### 3.3 Burden of three diseases by SDI

At the national level, SAIR and SAPR were strongly and positively associated with SDI for uterine cancer, and SADR and SAMR were not linearly and statistically associated with SDI. At the regional level, SAIR and SAPR for uterine cancer were strongly and positively associated with SDI, and SADR and SAMR were weakly and positively associated. 2 For type 2 diabetes, at the national level, SAIR and SAPR were nearly linearly associated, and SADR was moderately and negatively associated; at the regional level, there was no linear or statistically significant association for SAIR, a weakly and significantly negative association for SAPR, and no statistically significant associations for SADR and SAMR. There was no significant correlation between SAIR EAPC and SDI for these diseases. SAPR EAPC was weakly negatively correlated with SDI for polycystic ovary syndrome, but not for uterine cancer and type 2 diabetes; SADR EAPC was weakly negatively correlated with SDI for polycystic ovary syndrome, weakly positively correlated with type 2 diabetes, and not statistically significant for uterine cancer. SADR EAPC for uterine cancer and type 2 diabetes mellitus was negatively correlated with SDI, type 2 diabetes mellitus had a stronger and more significant correlation, and uterine cancer had a weaker but significant correlation, indicating that the effect of SDI on SADR EAPC varies according to disease (Figure 2; Supplementary Figures S4–S13).

### 3.4 Attributable burden of diabetes mellitus type 2 and uterine cancer caused by various risk factors

In 2021, metabolic risks and high fasting plasma glucose were responsible for 100% (95% UI: 100.00–100.00) of global deaths and DALYs related to diabetes mellitus type 2. Additionally, a high body mass index contributed to 62.87% (95% UI: 79.26, 35.28) of global deaths and 62.44% (95% UI: 35.24–78.73) of DALYs from Diabetes mellitus type 2 in the year, particularly in regions with high SDI quintiles. For uterine cancer, 30.76% (95% UI: 39.95–22.29) of global deaths and 30.57% (95% UI: 39.69–22.15) of DALYs were attributable to high BMI. In 2021, there was an increase in the prevalence of high BMI compared to 1990. High BMI and metabolic risk were associated with a higher burden of attributable mortality and DALYs for Diabetes mellitus type 2 and uterine cancer in regions with high SDI quintiles (Supplementary Table S8).

## 4 Discussion

Globally, the increasing incidence and prevalence of PCOS is particularly striking, with an age–standardized incidence rate (ASIR) of 64.44 per 100,000 population in 2021 (95% UI: 39.07–103.40). Among the 21 global regions, high-income Asia-Pacific had the highest ASIR of 308.16 per 100 000 population, with Japan leading the world with an ASIR of 360.92 per 100 000 population (95% UI: 199.08–573.59), and Southeast Asia had the highest change (EAPC) in ASIR of 1.89 (95% CI: 1.79–1.99) Figures 1, 3; Table 2 this discrepancy may be attributable to the advanced medical infrastructure and heightened health awareness prevalent in high-income regions, which facilitate a more timely and accurate diagnosis of the disease (18). Conversely, the rapid increase in incidence observed in South Asia may be attributable to factors such as accelerated urbanization, dietary shifts, and physical inactivity within the region (19). Critically, these findings underscore an urgent need for region-specific screening protocols and public education initiatives to improve early detection in rapidly developing economies, while high-resource settings should leverage diagnostic capacity for longitudinal outcome studies. There was significant heterogeneity in the regional distribution of uterine cancers in the same year. Globally, there has been a small increase in the ASIR for uterine cancer, with an EAPC of 0.44 (95% CI: 0.32–0.56), which may be attributed to advances in screening technology, leading to increased rates of early diagnosis. Regionally, Eastern Europe had the highest ASIR of 9.73 per 100 000 population (95% CI: 8.52–11.06), while the Caribbean had the highest age–standardized mortality rate (ASMR) of 1.47 (95% CI: 1.12–1.93) per 100 000 population. This mortality disparity likely reflects critical gaps in healthcare access, including limited availability of diagnostic services, delayed presentation, and suboptimal treatment pathways in resource-constrained settings. The high-income Asia-Pacific region had the highest increase in the EAPC of the ASIR, which reached 3.09 (95% CI: 2.89–3.29), highlighting the urgency of sustained investment in universal screening programs and awareness campaigns in high-risk populations, particularly where obesity rates are rising.

Of the three diseases analyzed here, type 2 diabetes (T2D) has the highest disease burden, with a global ASIR of 267.85 per 100,000 people (95% UI: 198.90–348.61) in 2021. Oceania has the highest ASIR at 659.24 per 100,000 people (95% UI: 492.70–833.65), and its DALY was also the highest at 1,334.68 years per 100,000 people (95% UI: 1,039.92–1,700.89). Globally, ASIR has an EAPC of 1.95 (95% CI: 1.91–1.99), showing a significant upward trend over the past 32 years. North Africa and the Middle East demonstrated the highest ASIR (EAPC) with a value of 3.41 (95% CI: 3.36–3.46), while Oceania exhibited the highest ASMR of 15.39 per 100,000 (95% UI: 11.50–20.33). This escalating burden demands prioritized integration of diabetes prevention into primary healthcare systems, emphasizing culturally adapted lifestyle interventions and affordable metformin access in high-growth regions.

It has been established that high-income regions, such as North America and the high-income Asia-Pacific region, have achieved favorable outcomes in terms of diagnosis and mortality rates for polycystic ovary syndrome (PCOS) and uterine cancer, a phenomenon that can be attributed to the advanced healthcare systems and comprehensive health education programs in place. However, the escalating burden of type 2 diabetes (T2D) in these regions underscores the rapid propagation of obesity and unhealthy lifestyles, a multifaceted predicament that defies effective resolution through the application of conventional disease management interventions (20). Consequently, innovative policy measures—such as sugar-sweetened beverage taxation, urban design promoting physical activity, and mandatory nutritional labeling—are essential complements to clinical management. Conversely, low-income regions such as sub-Saharan Africa and Oceania encounter significant challenges in addressing T2D and uterine cancer. Age-standardized mortality rates (ASMR) and disability-adjusted life years (DALYs) in these regions exceed global averages, necessitating targeted international aid for essential medicine procurement, task-shifting of screening to community health workers, and mobile health technologies for patient follow-up. For instance, in Italy, the high prevalence of PCOS and the high number of DALYs (age-standardized prevalence rate ASPR: 15,307.74 per 100,000 population; 95% UI: 10,698.08–21,343.59) reflects the significant impact of lifestyle factors and advances in diagnostic technology on the prevalence of the disease and on DALYs. The high incidence of uterine cancer in Russia is closely related to genetic susceptibility and the high prevalence of smoking in the region. Conversely, the elevated uterine cancer mortality rate observed in the Caribbean is primarily attributable to the absence of screening programs and advanced stages at which the disease is diagnosed in the region. Implementing low-cost visual inspection methods (e.g., acetic acid cervicography) could mitigate this gap where cytology infrastructure is lacking.

In this study, PCOS, uterine cancer, and T2D were collectively examined because of the close relationship between their pathogenesis, particularly in terms of metabolic abnormalities and hormonal imbalance (21). A fundamental characteristic of PCOS is insulin resistance, in which elevated insulin levels stimulate the ovaries to produce excessive androgenes, resulting in irregular menstruation and impaired ovulation (22). Insulin resistance not only contributes to PCOS development but also accelerates the development of T2D. Concurrently, elevated estrogen levels in women promote endometrial hyperplasia, which increases the risk of uterine cancer (23). T2D is often accompanied by obesity and metabolic syndrome (24). Chronic hyperglycemia leads to the accumulation of glycosylation end-products, which activate tumor-related signaling pathways and promote the development of uterine cancer (25). Recent evidence further elucidates how hyperinsulinemia directly stimulates endometrial cell proliferation via PI3K/Akt/mTOR pathways, creating a shared oncogenic microenvironment across these conditions (26). The prevalence of uterine cancer is commonly observed in conjunction with obesity and metabolic disorders, and obesity drives uterine cancer by increasing the estrogen and insulin levels. Obesity and chronic inflammation are the prevalent risk factors for all three diseases (27). Pro-inflammatory cytokines (e.g., IL-6, TNF-α) derived from adipose tissue not only exacerbate insulin resistance but also foster a tumor-promoting milieu in the endometrium, creating a biological continuum linking these conditions (28). Furthermore, hormonal fluctuations during the perimenopausal period have been demonstrated to exacerbate ovulation disorders and an imbalance in the estrogen-to-androgen ratio, thereby increasing the risk of type 2 diabetes (T2D) and uterine cancer (29). The high prevalence of T2D in the 45–49 year age group may be related to an age-related decline in insulin sensitivity; consequently, there is a close interaction between polycystic ovary syndrome (PCOS), uterine cancer, and type 2 diabetes mellitus (30). The present study found that DALYs and mortality rates of uterine cancer and type 2 diabetes mellitus were highly correlated with metabolic abnormalities and BMI, which further confirmed the trend of mutual increase in the incidence of these three (31). This shared pathophysiology strongly supports integrated clinical guidelines advocating for universal metabolic screening (e.g., HbA1c, lipid panel) in women diagnosed with PCOS or endometrial hyperplasia, and conversely, gynecological surveillance in women with T2D and obesity.

Despite the comprehensive and in-depth analysis of the global burden of the three diseases based on authoritative GBD 2021 data, certain limitations remain. This study was based on secondary data, and the variable quality of data in different regions, in addition to differences in data collection methods, may introduce potential bias, which in turn affects the accuracy and reliability of the results (32). Furthermore, the estimation of disease burden is highly susceptible to interference from differences in reporting standards and levels of diagnostic technology in different regions, which may lead to overestimation or underestimation of the incidence, prevalence, and mortality in each region. To further investigate the complex association between PCOS, uterine cancer, and type 2 diabetes, future studies should combine prospective cohort studies with mechanistic studies to conduct comprehensive analyses at molecular, cellular, and population levels. Concurrently, the impact of social, economic, psychological, and environmental factors on the disease burden must be thoroughly considered, thereby providing a robust theoretical foundation for the development of more scientific and effective public health intervention strategies. Specifically, research should prioritize: (1) Validation of simplified diagnostic criteria for PCOS in low-resource settings; (2) Cost-effectiveness analyses of combined metformin-lifestyle interventions for primary prevention in high-risk women; and (3) Implementation science studies on integrating endometrial cancer risk assessment into diabetes clinics.

## 5 Conclusion

In summary, the present study revealed complex interactions between the burden of PCOS, uterine cancer, and type 2 diabetes in a population of women of childbearing age as well as significant regional differences. These findings emphasize the necessity for the development of comprehensive public health strategies, and that multifactorial challenges can be effectively addressed only through concerted multifaceted efforts for global health development.

## Data Availability

The original contributions presented in the study are included in the article/Supplementary material, further inquiries can be directed to the corresponding authors.

## Footnotes

1
https://vizhub.healthdata.org/gbd-results
