# Supplementary material for: Global burden of polycystic ovary syndrome, uterine cancer and diabetes mellitus type 2 among women of childbearing age: trends in incidence, prevalence, mortality, and disability-adjusted life-years from 1990 to 2021
Source: Front Med (Lausanne). 2025 Jul 11;12:1628462. doi: 10.3389/fmed.2025.1628462 (PMC12289557; doi:10.3389/fmed.2025.1628462)
Supplement: Supplementary file 1 [file Data_Sheet_1.pdf]

Table S1 Regional prevalence rate and ASPR of PCOS,Uterine cancer,T2DM in 2021

2021	pcos		Uterine cancer		Diabetes mellitus type 2	
location	Prevalence (1/100,000, 95%UI)	APPR (1/100,000, 95%UI)	Prevalence (1/100,000, 95%UI)	ASPR (1/100,000, 95%UI)	Prevalence (1/100,000, 95%UI)	ASPR (1/100,000, 95%UI)
Global	3374.68(2394.97,4649.68)	3364.53(2395.08,4681.81)	24.83(21.44,27.56)	23.59(20.11,26.54)	3789.86(3386.79,4242.87)	3678.58(3154.75,4254.46)
High SDI	6868.85(5049.20,9340.23)	6825.02(5014.42,9336.43)	54.03(51.78,56.27)	43.91(41.61,46.23)	3922.04(3512.66,4347.99)	3457.05(2971.76,3984.94)
High-middle SDI	3456.66(2437.81,4832.38)	3466.45(2432.25,4866.13)	49.75(42.87,57.58)	39.18(33.13,46.36)	4248.74(3725.56,4829.01)	3720.31(3148.53,4364.63)
Middle SDI	3758.17(2662.53,5222.38)	3758.81(2638.95,5246.88)	21.72(16.27,25.81)	19.59(14.70,23.64)	4081.72(3648.89,4561.87)	3829.65(3292.00,4430.65)
Low-middle SDI	2272.04(1581.38,3197.84)	2273.50(1593.33,3221.85)	9.93(8.14,11.79)	10.80(8.69,13.23)	3667.17(3238.79,4146.11)	3857.22(3263.74,4504.79)
Low SDI	1358.55(948.62,1927.76)	1369.98(948.19,1943.79)	5.63(4.23,7.49)	7.28(5.38,9.81)	2728.66(2432.62,3086.16)	3153.96(2679.11,3677.48)
Andean Latin America	6332.51(4336.27,8867.70)	6305.75(4312.97,8986.98)	27.32(20.17,36.05)	27.76(19.33,39.31)	2255.78(2003.00,2495.86)	2268.76(1945.04,2610.87)
Australasia	9213.66(6561.16,12814.35)	9156.94(6439.94,12788.23)	35.57(30.44,41.82)	30.03(23.55,37.75)	1511.17(1263.43,1781.89)	1329.42(1053.33,1640.92)
Caribbean	2825.76(1918.97,4075.49)	2823.51(1912.23,4041.39)	46.68(38.36,56.16)	45.02(35.97,55.55)	6097.90(5419.25,6777.38)	5960.67(5082.96,6914.99)
Central Asia	929.26(639.64,1304.20)	923.53(622.33,1311.00)	29.54(24.81,34.79)	28.50(23.72,33.79)	3300.25(2956.19,3675.39)	3169.65(2727.23,3661.85)
Central Europe	436.25(297.91,615.30)	436.26(297.37,620.93)	61.52(54.35,69.48)	45.41(39.63,51.79)	2020.61(1720.88,2328.98)	1516.81(1236.25,1833.31)
Central Latin America	5582.44(3878.65,7803.84)	5580.86(3873.19,7846.44)	19.82(16.92,22.89)	19.24(16.26,22.51)	5758.98(5122.27,6401.41)	5658.61(4841.85,6531.21)
Central Sub-Saharan Africa	1281.70(886.26,1849.84)	1291.50(880.09,1861.86)	5.45(3.39,8.08)	7.18(4.06,11.70)	2782.66(2445.74,3185.83)	3175.63(2670.21,3734.78)
East Asia	2983.92(2103.55,4236.17)	2967.58(2057.24,4219.21)	36.79(25.84,51.42)	28.87(19.66,40.32)	5406.86(4734.34,6177.52)	4825.37(4077.94,5668.03)
Eastern Europe	520.31(363.73,752.90)	507.61(344.81,744.99)	108.32(95.72,122.05)	81.37(71.28,92.44)	2240.77(1865.16,2581.35)	1746.69(1393.84,2123.38)
Eastern Sub-Saharan Africa	1272.44(894.04,1817.39)	1282.62(885.52,1826.00)	5.96(4.09,9.00)	8.00(5.35,12.32)	1416.75(1231.99,1605.61)	1642.75(1371.48,1940.27)
High-income Asia Pacific	10239.02(7234.28,14296.03)	10116.87(7086.92,14260.97)	53.28(48.31,59.14)	38.04(32.96,43.83)	4539.75(4034.83,5053.73)	3803.02(3232.13,4418.41)
High-income North America	7225.93(5394.79,9514.86)	7200.01(5306.10,9639.58)	75.67(72.24,79.09)	66.42(62.64,70.54)	3751.49(3373.52,4127.35)	3422.88(2951.47,3927.27)
North Africa and Middle East	3975.80(2791.13,5616.82)	3968.60(2762.78,5641.91)	17.51(13.03,20.96)	17.50(12.77,21.60)	4950.04(4389.30,5498.50)	4927.18(4215.01,5702.08)
Oceania	3393.43(2360.22,4846.83)	3384.68(2326.80,4838.62)	21.68(11.60,37.03)	24.33(12.52,42.68)	8303.13(7419.92,9288.47)	8802.82(7547.50,10199.76)
South Asia	2175.38(1537.88,3046.40)	2174.75(1528.67,3058.06)	6.52(5.18,8.91)	6.95(5.40,9.63)	4027.39(3517.61,4578.55)	4175.46(3503.05,4910.33)
Southeast Asia	5457.54(3851.15,7695.68)	5444.39(3819.23,7698.36)	25.45(16.78,32.03)	23.93(14.92,30.82)	2421.70(2124.50,2716.42)	2312.24(1930.01,2707.58)
Southern Latin America	3658.16(2573.53,5251.35)	3637.79(2531.50,5240.58)	16.13(14.23,18.37)	14.93(11.95,18.70)	2347.58(1985.86,2686.61)	2191.01(1778.06,2607.20)
Southern Sub-Saharan Africa	2103.88(1439.47,2969.21)	2094.17(1431.86,2995.98)	10.74(8.44,13.82)	11.44(8.70,15.22)	2572.73(2231.94,2943.86)	2629.14(2177.97,3119.72)
Tropical Latin America	1145.47(777.94,1624.71)	1140.94(776.47,1632.49)	17.32(16.18,18.55)	15.68(14.16,17.24)	2566.89(2189.37,2946.18)	2360.26(1900.07,2868.62)
Western Europe	7518.71(5296.66,10491.12)	7493.55(5251.94,10400.43)	44.03(41.73,46.64)	34.03(31.37,36.93)	3459.45(2995.94,3980.69)	3071.40(2525.48,3658.72)
Western Sub-Saharan Africa	1371.94(959.89,1957.59)	1385.41(951.70,1970.02)	3.53(2.57,4.88)	4.70(3.36,6.62)	2365.69(2095.39,2690.30)	2707.16(2286.16,3166.94)

ASPR age-standardized prevalence rate, UI uncertainty intervals .PCOS Polycystic ovary syndrome , T2DM type 2 diabetes mellitus

Table S2 Regional DALYs and ASDR of PCOS,Uterine cancer,T2DM in 2021

location	pcos		Uterine cancer		Diabetes mellitus type 2	
	DALYs (1/100,000, 95%UI)	ASDR (1/100,000, 95%UI)	DALYs (1/100,000, 95%UI)	ASDR (1/100,000, 95%UI)	DALYs (1/100,000, 95%UI)	ASDR (1/100,000, 95%UI)
Global	29.56(13.24,61.66)	29.51(13.09,61.49)	19.17(15.86,21.73)	18.29(15.09,20.88)	353.83(271.12,451.44)	341.29(258.87,443.27)
High SDI	60.40(27.50,123.44)	60.32(27.37,122.77)	22.66(21.35,24.17)	18.52(17.33,19.90)	287.35(205.17,383.15)	248.08(173.64,339.05)
High-middle SDI	30.07(13.34,63.54)	30.38(13.37,64.03)	28.62(24.40,33.49)	22.83(19.11,27.20)	310.18(218.49,420.15)	264.54(182.97,368.26)
Middle SDI	32.85(14.59,69.05)	32.95(14.39,69.38)	20.71(15.45,24.41)	18.78(14.05,22.48)	388.00(299.00,492.06)	358.48(272.16,465.23)
Low-middle SDI	20.00(8.71,42.13)	19.97(8.66,41.98)	14.25(11.65,17.34)	15.39(12.27,19.33)	386.17(306.75,486.57)	412.23(320.20,526.10)
Low SDI	11.89(5.10,25.14)	11.91(5.10,25.15)	11.11(8.33,14.89)	14.13(10.42,19.15)	323.67(261.87,402.99)	388.44(309.71,492.57)
Andean Latin America	54.70(24.02,114.59)	54.46(23.94,114.59)	29.19(21.56,38.15)	29.61(20.69,40.47)	265.82(213.16,334.06)	268.64(208.75,348.19)
Australasia	80.23(36.04,166.50)	79.97(35.50,166.66)	14.87(12.99,17.15)	12.60(10.23,15.44)	125.64(89.80,168.35)	109.37(75.82,152.69)
Caribbean	24.79(10.76,52.32)	24.79(10.61,52.51)	64.43(51.62,80.77)	62.39(47.26,81.73)	778.33(604.47,1012.47)	757.95(569.86,1009.03)
Central Asia	8.13(3.43,17.55)	8.10(3.40,17.41)	30.45(25.15,36.16)	29.35(24.16,35.43)	305.85(223.53,409.29)	295.67(212.52,404.98)
Central Europe	3.78(1.62,7.95)	3.81(1.62,8.15)	35.65(31.53,40.20)	26.64(23.29,30.23)	192.32(136.35,265.19)	144.24(101.20,198.89)
Central Latin America	48.43(21.06,101.44)	48.44(21.12,102.24)	22.06(18.72,25.90)	21.44(18.05,25.23)	710.29(560.54,890.33)	693.86(539.01,885.94)
Central Sub-Saharan Africa	11.15(4.81,22.90)	11.15(4.74,23.40)	11.14(6.88,16.38)	14.49(8.16,23.68)	369.90(288.30,483.15)	445.03(332.53,593.22)
East Asia	25.60(11.20,53.07)	25.64(11.15,53.82)	26.54(18.60,36.40)	21.12(14.40,29.37)	344.71(232.75,480.92)	299.64(198.05,426.40)
Eastern Europe	4.56(1.89,9.56)	4.51(1.86,9.53)	57.72(50.14,66.14)	43.81(37.75,50.99)	205.85(148.78,272.15)	159.02(114.39,215.70)
Eastern Sub-Saharan Africa	11.06(4.73,23.38)	11.07(4.74,23.42)	11.97(8.20,18.33)	15.80(10.57,24.58)	248.35(208.08,303.57)	305.48(249.65,378.20)
High-income Asia Pacific	88.65(39.90,179.93)	88.17(39.21,180.74)	27.19(25.02,29.49)	19.36(17.59,21.33)	322.81(218.94,449.39)	260.68(174.73,373.17)
High-income North America	63.90(29.12,129.28)	63.85(29.11,127.64)	27.25(25.43,29.34)	24.02(22.35,25.97)	292.07(218.02,384.12)	263.02(193.52,350.61)
North Africa and Middle East	35.28(15.76,74.74)	35.25(15.48,74.26)	11.95(9.19,14.38)	11.93(8.91,14.87)	441.55(333.92,579.79)	441.13(321.50,595.30)
Oceania	29.55(12.80,61.92)	29.44(12.73,61.41)	34.58(17.63,61.37)	38.47(19.40,69.70)	1225.86(995.69,1548.32)	1334.68(1039.92,1700.89)
South Asia	19.09(8.27,39.99)	19.06(8.21,39.87)	10.07(7.79,13.94)	10.68(8.08,15.08)	374.59(286.87,480.34)	393.88(297.37,510.75)
Southeast Asia	48.10(21.32,99.17)	48.06(20.99,99.85)	28.23(18.03,35.90)	26.63(16.31,34.48)	361.25(301.74,435.67)	342.77(279.81,424.25)
Southern Latin America	32.34(14.05,67.18)	32.22(13.93,66.95)	13.67(12.08,15.48)	12.71(10.28,15.71)	202.31(147.42,270.62)	188.00(133.95,255.45)
Southern Sub-Saharan Africa	18.22(7.80,38.40)	18.14(7.73,38.63)	17.76(13.65,22.93)	18.81(13.89,25.55)	418.62(354.68,501.26)	441.66(361.91,537.55)
Tropical Latin America	10.09(4.30,21.29)	10.08(4.28,21.62)	19.10(17.88,20.41)	17.38(15.91,18.99)	334.85(265.81,414.20)	306.13(240.03,389.68)
Western Europe	66.74(30.23,138.62)	66.97(29.95,140.01)	16.30(15.15,17.65)	12.74(11.62,14.07)	228.06(152.72,319.13)	197.66(129.17,282.54)
Western Sub-Saharan Africa	11.96(5.12,25.51)	11.98(5.11,25.47)	6.71(4.87,9.30)	8.78(6.25,12.44)	286.02(223.56,361.75)	341.68(263.98,439.32)

ASDR age-standardized DALYs rate, UI uncertainty intervals .PCOS Polycystic ovary syndrome , T2DM type 2 diabetes mellitus

Table S3 Regional mortality and ASMR of PCOS,Uterine cancer,T2DM in 2021

location	PCOS Mortality (1/100,000, 95%UI)	ASMR (1/100,000, 95%UI)	Uterine cancer Mortality (1/100,000, 95%UI)	ASMR (1/100,000, 95%UI)	Diabetes mellitus type 2 Mortality (1/100,000, 95%UI)	ASMR (1/100,000, 95%UI)
Global	-	-	0.37(0.31，0.41)	0.42(0.35，0.48)	2.16(1.96，2.36)	2.06(1.85，2.28)
High SDI	-	-	0.41(0.39，0.43)	0.40(0.38，0.42)	0.85(0.80，0.92)	0.70(0.64，0.78)
High-middle SDI	-	-	0.54(0.46，0.63)	0.51(0.43，0.61)	0.87(0.79，0.99)	0.70(0.62，0.80)
Middle SDI	-	-	0.40(0.31，0.47)	0.44(0.33，0.52)	2.45(2.26，2.63)	2.21(2.02，2.42)
Low-middle SDI	-	-	0.28(0.23，0.34)	0.37(0.29，0.46)	2.91(2.51，3.30)	3.16(2.69，3.66)
Low SDI	-	-	0.22(0.16，0.29)	0.34(0.25，0.46)	2.71(2.28，3.15)	3.41(2.82，4.02)
Andean Latin America	-	-	0.58(0.42，0.75)	0.70(0.49，0.96)	2.17(1.73，2.71)	2.21(1.68，2.87)
Australasia	-	-	0.27(0.24，0.31)	0.27(0.23，0.33)	0.50(0.46，0.55)	0.43(0.37，0.49)
Caribbean	-	-	1.27(1.01，1.61)	1.47(1.12，1.93)	5.50(4.26，7.03)	5.32(3.87，7.21)
Central Asia	-	-	0.59(0.49，0.70)	0.68(0.56，0.82)	1.40(1.16，1.66)	1.37(1.13，1.65)
Central Europe	-	-	0.68(0.61，0.76)	0.60(0.53，0.68)	0.66(0.60，0.73)	0.52(0.46，0.58)
Central Latin America	-	-	0.44(0.37，0.51)	0.51(0.43，0.60)	5.55(4.74，6.32)	5.39(4.60，6.18)
Central Sub-Saharan Africa	-	-	0.22(0.14，0.33)	0.36(0.20，0.58)	3.37(2.43，4.59)	4.26(2.84，6.15)
East Asia	-	-	0.51(0.36，0.69)	0.48(0.33，0.66)	0.64(0.49，0.81)	0.49(0.37，0.64)
Eastern Europe	-	-	1.08(0.94，1.25)	0.97(0.84，1.14)	0.82(0.73，0.92)	0.64(0.56，0.72)
Eastern Sub-Saharan Africa	-	-	0.23(0.16，0.36)	0.38(0.25，0.59)	2.71(2.20，3.27)	3.51(2.81，4.28)
High-income Asia Pacific	-	-	0.52(0.48，0.55)	0.43(0.40，0.47)	0.33(0.30，0.37)	0.25(0.21，0.30)
High-income North America	-	-	0.48(0.46，0.50)	0.50(0.48，0.53)	1.26(1.20，1.32)	1.10(1.05，1.16)
North Africa and Middle East	-	-	0.23(0.17，0.27)	0.27(0.20，0.34)	2.19(1.82，2.66)	2.21(1.79，2.72)
Oceania	-	-	0.69(0.35，1.23)	0.93(0.47，1.68)	13.82(10.85，17.44)	15.39(11.50，20.33)
South Asia	-	-	0.20(0.15，0.27)	0.25(0.19，0.36)	2.43(2.05，2.83)	2.61(2.15，3.16)
Southeast Asia	-	-	0.56(0.36，0.71)	0.63(0.38，0.82)	3.82(3.27，4.53)	3.60(3.01，4.34)
Southern Latin America	-	-	0.26(0.23，0.29)	0.29(0.23，0.36)	1.00(0.91，1.10)	0.93(0.81，1.05)
Southern Sub-Saharan Africa	-	-	0.35(0.27，0.46)	0.45(0.34，0.61)	4.79(4.20，5.45)	5.18(4.36，6.12)
Tropical Latin America	-	-	0.38(0.36，0.40)	0.41(0.38，0.45)	2.70(2.57，2.83)	2.46(2.31，2.62)
Western Europe	-	-	0.29(0.28，0.31)	0.27(0.25，0.29)	0.28(0.27，0.29)	0.22(0.21，0.23)
Western Sub-Saharan Africa	-	-	0.13(0.10，0.18)	0.21(0.15，0.30)	2.46(1.77，3.10)	3.09(2.22，3.96)

ASMR age-standardized mortality rate, UI uncertainty intervals .PCOS Polycystic ovary syndrome , T2DM type 2 diabetes mellitus

Table S4 EAPC of ASIR, ASPR, ASDR and ASMR for PCOS ,Uterine cancer,T2DM in Regional from 1990 to 2021.

	**PCOS**				**Uterine cancer**				**Diabetes mellitus type 2**			
**location_name**	**EAPC, 95% CI**				**EAPC, 95% CI**				**EAPC, 95% CI**			
	ASIR	ASPR	ASDR	ASMR	ASIR	ASPR	ASDR	ASMR	ASIR	ASPR	ASDR	ASMR
Global	0.65(0.62,0.69)	0.74(0.70,0.77)	0.72(0.68,0.76)	-	0.44(0.32,0.56)	0.55(0.43,0.67)	-1.24(-1.34,-1.13)	-1.34(-1.45,-1.23)	1.95(1.91,1.99)	2.43(2.34,2.52)	1.51(1.44,1.58)	0.01(-0.11,0.13)
High SDI	0.21(-0.05,0.46)	0.10(-0.08,0.27)	0.08(-0.09,0.25)	-	1.78(1.69,1.87)	1.83(1.73,1.92)	0.40(0.30,0.51)	0.20(0.09,0.32)	2.62(2.55,2.69)	3.39(3.32,3.46)	2.21(2.12,2.31)	-1.17(-1.33,-1.02)
High-middle SDI	1.37(1.30,1.45)	1.21(1.17,1.25)	1.20(1.16,1.25)	-	0.35(0.20,0.51)	0.46(0.31,0.62)	-1.90(-2.10,-1.70)	-2.10(-2.31,-1.88)	1.88(1.81,1.96)	2.67(2.48,2.85)	1.78(1.65,1.92)	-1.23(-1.39,-1.07)
Middle SDI	1.39(1.34,1.43)	1.73(1.69,1.78)	1.72(1.67,1.77)	-	0.41(0.28,0.55)	0.62(0.48,0.76)	-1.78(-1.90,-1.66)	-1.87(-1.98,-1.75)	1.48(1.42,1.54)	1.91(1.78,2.03)	1.04(0.94,1.13)	-0.37(-0.55,-0.18)
Low-middle SDI	1.28(1.23,1.32)	1.63(1.59,1.68)	1.59(1.55,1.63)	-	1.00(0.92,1.08)	1.11(1.03,1.19)	-0.10(-0.17,-0.03)	-0.14(-0.21,-0.07)	2.35(2.32,2.39)	2.44(2.40,2.47)	1.39(1.33,1.44)	0.24(0.19,0.29)
Low SDI	0.92(0.89,0.94)	1.23(1.20,1.26)	1.21(1.19,1.24)	-	0.48(0.33,0.64)	0.65(0.48,0.81)	-0.35(-0.46,-0.25)	-0.40(-0.50,-0.29)	2.10(2.07,2.13)	2.28(2.25,2.32)	0.69(0.61,0.77)	-0.66(-0.77,-0.56)
Andean Latin America	0.74(0.64,0.83)	1.09(1.01,1.16)	1.06(0.99,1.14)	-	0.40(0.15,0.64)	0.58(0.33,0.83)	-1.46(-1.69,-1.23)	-1.55(-1.78,-1.31)	2.23(2.13,2.32)	2.23(2.13,2.34)	0.81(0.72,0.90)	-0.50(-0.70,-0.30)
Australasia	0.36(0.29,0.42)	0.27(0.18,0.37)	0.28(0.19,0.37)	-	1.48(1.30,1.66)	1.54(1.36,1.73)	0.41(0.24,0.57)	0.27(0.10,0.44)	1.79(1.74,1.84)	2.27(2.15,2.39)	1.34(1.24,1.43)	-1.08(-1.42,-0.75)
Caribbean	0.68(0.63,0.72)	0.76(0.70,0.83)	0.75(0.68,0.82)	-	0.43(0.06,0.80)	0.43(0.05,0.80)	0.34(0.10,0.57)	0.34(0.11,0.57)	2.03(1.99,2.06)	2.28(2.24,2.33)	1.17(1.08,1.27)	-0.29(-0.47,-0.11)
Central Asia	0.88(0.82,0.94)	1.18(1.11,1.25)	1.16(1.09,1.23)	-	-0.53(-0.75,-0.30)	-0.44(-0.67,-0.22)	-1.57(-1.77,-1.38)	-1.63(-1.83,-1.43)	3.05(2.92,3.18)	2.97(2.85,3.09)	2.25(2.05,2.45)	0.57(0.08,1.05)
Central Europe	0.19(0.07,0.32)	0.64(0.59,0.70)	0.64(0.59,0.69)	-	0.61(0.47,0.74)	0.71(0.58,0.84)	-1.24(-1.43,-1.06)	-1.42(-1.60,-1.24)	1.33(1.29,1.37)	1.23(1.18,1.27)	0.75(0.68,0.82)	-0.97(-1.29,-0.66)
Central Latin America	-0.12(-0.27,0.02)	-0.09(-0.26,0.08)	-0.10(-0.27,0.06)	-	2.03(1.55,2.50)	2.19(1.72,2.65)	0.72(0.21,1.24)	0.65(0.13,1.17)	1.17(1.08,1.27)	1.39(1.28,1.50)	0.58(0.42,0.75)	-0.46(-0.98,0.06)
Central Sub-Saharan Africa	0.96(0.84,1.08)	1.28(1.13,1.43)	1.27(1.11,1.44)	-	0.54(0.29,0.79)	0.70(0.43,0.97)	-0.24(-0.39,-0.09)	-0.26(-0.41,-0.12)	2.32(2.29,2.35)	2.45(2.41,2.49)	0.98(0.94,1.02)	-0.17(-0.23,-0.11)
East Asia	1.59(1.44,1.73)	2.04(1.88,2.19)	2.05(1.89,2.22)	-	0.68(0.28,1.08)	0.94(0.52,1.36)	-2.51(-2.87,-2.16)	-2.71(-3.07,-2.35)	1.43(1.30,1.57)	2.59(2.30,2.89)	1.79(1.53,2.04)	-2.21(-2.51,-1.92)
Eastern Europe	0.75(0.72,0.78)	0.99(0.94,1.04)	0.98(0.93,1.03)	-	0.39(-0.04,0.83)	0.44(0.00,0.87)	-1.10(-1.50,-0.69)	-1.25(-1.66,-0.84)	2.22(2.13,2.30)	1.74(1.63,1.85)	1.54(1.36,1.72)	0.26(-0.65,1.18)
Eastern Sub-Saharan Africa	0.64(0.61,0.67)	0.86(0.83,0.89)	0.85(0.82,0.88)	-	0.20(0.04,0.35)	0.41(0.24,0.58)	-0.68(-0.81,-0.56)	-0.72(-0.84,-0.59)	1.31(1.23,1.40)	1.51(1.43,1.59)	-0.69(-0.83,-0.54)	-1.74(-1.90,-1.58)
High-income Asia Pacific	0.66(0.59,0.74)	0.27(0.22,0.32)	0.27(0.22,0.31)	-	3.09(2.89,3.29)	3.16(2.97,3.36)	0.84(0.60,1.07)	0.63(0.38,0.88)	2.16(2.04,2.28)	3.17(3.03,3.31)	2.22(2.03,2.41)	-3.75(-3.99,-3.51)
High-income North America	-0.24(-0.89,0.42)	-0.50(-1.01,0.01)	-0.51(-1.02,-0.01)	-	2.36(2.25,2.47)	2.37(2.26,2.49)	1.69(1.54,1.85)	1.55(1.39,1.71)	2.83(2.78,2.87)	3.90(3.79,4.02)	2.08(2.01,2.15)	-1.15(-1.37,-0.93)
North Africa and Middle East	0.83(0.76,0.90)	1.09(1.02,1.16)	1.04(0.96,1.11)	-	1.66(1.45,1.88)	1.75(1.54,1.96)	-0.42(-0.67,-0.18)	-0.59(-0.83,-0.34)	3.41(3.36,3.46)	3.44(3.40,3.49)	2.29(2.19,2.39)	0.14(-0.01,0.29)
Oceania	0.75(0.61,0.88)	0.81(0.64,0.99)	0.81(0.64,0.99)	-	0.48(0.40,0.55)	0.49(0.41,0.57)	0.47(0.43,0.52)	0.46(0.42,0.51)	2.56(2.53,2.60)	2.55(2.52,2.58)	1.05(1.00,1.11)	0.21(0.11,0.32)
South Asia	1.64(1.53,1.75)	2.14(2.01,2.27)	2.08(1.97,2.20)	-	1.21(1.01,1.41)	1.35(1.14,1.55)	-0.09(-0.22,0.04)	-0.16(-0.30,-0.02)	2.18(2.12,2.24)	2.31(2.28,2.34)	1.55(1.45,1.65)	0.46(0.34,0.58)

Table S4 EAPC of ASIR, ASPR, ASDR and ASMR for PCOS ,Uterine cancer,T2DM in Regional from 1990 to 2021. (continued)

location_name	PCOS EAPC, 95% CI				Uterine cancer EAPC, 95% CI				Diabetes mellitus type 2 EAPC, 95% CI			
	ASIR	ASPR	ASDR	ASMR	ASIR	ASPR	ASDR	ASMR	ASIR	ASPR	ASDR	ASMR
Southeast Asia	1.89(1.79,1.99)	2.30(2.19,2.40)	2.26(2.16,2.36)	-	0.89(0.82,0.97)	0.97(0.89,1.05)	-0.15(-0.26,-0.05)	-0.18(-0.28,-0.08)	1.21(0.95,1.47)	1.00(0.69,1.32)	0.04(-0.12,0.20)	-0.56(-0.71,-0.42)
Southern Latin America	1.42(1.22,1.63)	1.46(1.24,1.69)	1.46(1.24,1.68)	-	-1.02(-1.32,-0.72)	-0.95(-1.24,-0.65)	-2.33(-2.69,-1.97)	-2.45(-2.82,-2.08)	2.36(2.28,2.45)	3.12(3.05,3.20)	1.26(1.16,1.36)	-1.46(-1.66,-1.26)
Southern Sub-Saharan Africa	0.56(0.49,0.63)	0.79(0.70,0.87)	0.75(0.67,0.83)	-	1.75(1.28,2.22)	1.71(1.25,2.18)	1.41(0.91,1.93)	1.46(0.97,1.94)	1.46(1.39,1.53)	1.38(1.28,1.48)	1.35(0.97,1.74)	1.54(0.90,2.19)
Tropical Latin America	-0.33(-0.54,-0.12)	-0.17(-0.35,0.00)	-0.19(-0.37,-0.01)	-	0.59(0.38,0.81)	0.70(0.48,0.91)	-0.55(-0.72,-0.37)	-0.65(-0.82,-0.48)	0.81(0.70,0.91)	0.99(0.90,1.07)	-0.49(-0.64,-0.33)	-1.96(-2.17,-1.75)
Western Europe	0.21(0.19,0.24)	0.21(0.14,0.28)	0.22(0.15,0.28)	-	1.35(1.07,1.64)	1.41(1.12,1.70)	-0.02(-0.20,0.16)	-0.23(-0.41,-0.06)	2.65(2.58,2.73)	3.10(3.02,3.18)	2.32(2.22,2.42)	-2.58(-2.70,-2.46)
Western Sub-Saharan Africa	0.64(0.51,0.78)	0.92(0.74,1.11)	0.92(0.74,1.11)	-	0.70(0.61,0.79)	0.81(0.70,0.92)	-0.07(-0.13,-0.01)	-0.11(-0.17,-0.05)	1.92(1.88,1.95)	2.20(2.14,2.25)	1.00(0.89,1.12)	-0.02(-0.20,0.16)

PCOS polycystic ovary syndrome, T2DM type 2 diabetes mellitus, DALYs disability-adjusted life-years, ASIR age-standardized incidence rate, ASPR age-standardized prevalence rate, ASMR age-standardized mortality rate, ASDR age-standardized DALYs rate, EAPC estimated annual percentage change, CI confdence interval, UI uncertainty intervals

Table S5 The ASIR, ASPR,and ASDR of PCOS in 204 countries and territories in 2021,and the EAPC from 1990 to 2021.

location_name	SAIR (1/100,000, 95%UI)	EAPC ASIR (EAPC, 95% CI)	SAPR (1/100,000, 95%UI)	EAPC SAPR (EAPC, 95% CI)	SADR (1/100,000, 95%UI)	EAPC SADR (EAPC, 95% CI)	SAMR	EAPC SAMR
Afghanistan	49.94(30.38,83.64)	1.42(1.19,1.66)	2480.11(1691.03,3536.79)	1.68(1.40,1.97)	21.28(9.15,45.71)	1.73(1.44,2.02)	-	-
Albania	8.00(4.44,13.95)	0.81(0.74,0.87)	364.43(237.51,546.81)	0.86(0.76,0.95)	3.23(1.28,7.16)	0.87(0.77,0.98)	-	-
Algeria	75.04(46.85,124.47)	1.17(1.12,1.22)	4213.51(2861.57,6046.05)	1.40(1.36,1.45)	37.15(16.04,77.88)	1.37(1.31,1.43)	-	-
American Samoa	92.92(58.83,154.55)	0.78(0.63,0.94)	5315.55(3642.68,7714.10)	0.90(0.72,1.08)	46.56(20.13,98.06)	0.89(0.71,1.07)	-	-
Andorra	116.35(74.21,185.23)	0.66(0.57,0.76)	6819.98(4695.82,9646.27)	0.64(0.54,0.75)	61.14(26.92,127.49)	0.63(0.53,0.74)	-	-
Angola	30.77(17.56,52.79)	1.34(1.22,1.45)	1407.11(940.01,2045.79)	1.80(1.65,1.95)	12.16(5.08,25.74)	1.82(1.67,1.96)	-	-
Antigua and Barbuda	51.35(32.29,85.09)	0.71(0.67,0.75)	2997.85(2026.30,4278.36)	0.81(0.75,0.86)	26.50(11.39,55.72)	0.80(0.75,0.86)	-	-
Argentina	81.04(47.31,133.57)	1.34(1.17,1.50)	3450.24(2404.42,4962.20)	1.35(1.17,1.52)	30.61(13.31,63.72)	1.35(1.17,1.52)	-	-
Armenia	19.46(11.25,33.70)	1.19(1.15,1.23)	912.99(611.24,1337.51)	1.43(1.38,1.49)	7.91(3.34,17.08)	1.41(1.36,1.47)	-	-
Australia	216.47(126.22,358.38)	0.45(0.39,0.50)	8818.88(6166.58,12413.72)	0.38(0.25,0.50)	77.03(34.06,160.76)	0.38(0.26,0.50)	-	-
Austria	124.51(80.49,203.71)	0.08(0.03,0.14)	7515.50(5167.94,10643.50)	-0.05(-0.13,0.03)	66.69(28.68,138.00)	-0.09(-0.18,-0.00)	-	-
Azerbaijan	19.82(11.47,34.25)	1.28(1.18,1.37)	965.52(640.19,1409.11)	1.73(1.61,1.85)	8.53(3.46,18.66)	1.74(1.61,1.86)	-	-
Bahamas	56.44(35.47,91.46)	0.46(0.40,0.52)	3409.45(2280.10,4948.02)	0.52(0.44,0.59)	30.16(13.02,65.18)	0.51(0.44,0.59)	-	-
Bahrain	79.89(49.15,134.23)	0.39(0.35,0.43)	4579.65(3144.31,6574.31)	0.40(0.35,0.46)	40.40(17.52,85.50)	0.41(0.36,0.45)	-	-
Bangladesh	28.13(16.52,47.49)	1.51(1.38,1.64)	1178.89(801.19,1679.62)	1.88(1.74,2.01)	10.43(4.33,21.72)	1.84(1.71,1.96)	-	-
Barbados	55.19(34.59,89.39)	0.44(0.40,0.47)	3318.26(2246.66,4723.37)	0.47(0.44,0.51)	29.32(12.50,62.49)	0.47(0.43,0.50)	-	-
Belarus	12.60(6.97,22.26)	0.87(0.82,0.92)	508.97(338.42,757.64)	1.12(1.04,1.20)	4.50(1.79,9.85)	1.11(1.03,1.19)	-	-
Belgium	114.35(73.54,179.58)	0.45(0.27,0.63)	6761.69(4714.53,9503.07)	0.43(0.23,0.62)	60.37(26.77,127.28)	0.39(0.20,0.58)	-	-
Belize	54.91(34.80,89.64)	0.82(0.62,1.02)	3225.15(2195.05,4575.62)	0.97(0.70,1.24)	28.77(12.22,60.80)	0.94(0.68,1.20)	-	-
Benin	34.19(19.61,58.68)	1.39(1.19,1.59)	1608.92(1080.11,2309.23)	1.85(1.57,2.13)	13.77(5.83,29.37)	1.83(1.56,2.10)	-	-
Bermuda	61.18(39.54,94.04)	0.28(0.23,0.33)	3800.59(2561.92,5529.51)	0.24(0.18,0.30)	33.65(14.29,70.36)	0.24(0.19,0.30)	-	-
Bhutan	38.72(22.68,65.73)	1.66(1.56,1.76)	1821.77(1236.37,2635.66)	2.11(1.99,2.24)	16.13(6.82,33.88)	2.12(2.00,2.25)	-	-
Bolivia (Plurinational State of)	88.26(57.51,142.20)	0.77(0.73,0.81)	5703.66(3820.50,8100.56)	1.00(0.92,1.08)	49.24(21.31,106.97)	0.98(0.91,1.06)	-	-
Bosnia and Herzegovina	7.84(4.28,13.68)	1.26(1.10,1.42)	364.77(234.91,551.34)	1.59(1.36,1.81)	3.18(1.24,7.03)	1.57(1.35,1.79)	-	-
Botswana	41.87(24.78,70.52)	1.27(1.11,1.43)	2066.34(1398.36,2952.68)	1.67(1.46,1.89)	17.63(7.58,37.11)	1.66(1.45,1.87)	-	-
Brazil	24.06(14.53,40.50)	-0.36(-0.58,-0.15)	1140.88(776.65,1631.48)	-0.21(-0.39,-0.03)	10.08(4.29,21.62)	-0.23(-0.41,-0.05)	-	-

Table S5 The ASIR, ASPR,and ASDR of PCOS in 204 countries and territories in 2021,and the EAPC from 1990 to 2021. (continued)

location_name	SAIR (1/100,000, 95%UI)	EAPC ASIR (EAPC, 95% CI)	SAPR (1/100,000, 95%UI)	EAPC SAPR (EAPC, 95% CI)	SADR (1/100,000, 95%UI)	EAPC SADR (EAPC, 95% CI)	SAMR	EAPC SAMR
Brunei Darussalam	228.76(125.51,363.60)	1.57(1.40,1.74)	7355.42(5060.01,10478.12)	1.51(1.35,1.67)	64.19(28.69,132.53)	1.50(1.34,1.65)	-	-
Bulgaria	8.69(4.94,14.98)	0.72(0.68,0.76)	417.23(272.36,623.57)	0.82(0.78,0.86)	3.62(1.44,7.92)	0.83(0.79,0.87)	-	-
Burkina Faso	29.53(16.91,50.49)	0.95(0.80,1.10)	1324.59(898.02,1907.54)	1.29(1.08,1.50)	11.45(4.80,24.31)	1.22(1.03,1.42)	-	-
Burundi	22.19(12.68,37.73)	0.10(0.05,0.15)	884.19(593.73,1264.18)	0.09(0.02,0.17)	7.64(3.16,16.24)	0.10(0.00,0.19)	-	-
Cabo Verde	33.87(19.78,57.06)	1.07(0.91,1.23)	1602.44(1084.46,2335.39)	1.45(1.23,1.67)	13.81(5.84,29.64)	1.44(1.23,1.66)	-	-
Cambodia	84.48(50.48,137.99)	1.58(1.53,1.63)	3852.40(2652.31,5494.77)	1.88(1.85,1.92)	33.48(14.52,69.65)	1.94(1.89,1.99)	-	-
Cameroon	36.17(20.95,62.16)	0.57(0.49,0.64)	1765.28(1167.93,2580.57)	0.74(0.64,0.84)	15.48(6.57,32.82)	0.74(0.64,0.85)	-	-
Canada	87.47(51.96,140.96)	0.82(0.70,0.94)	3546.13(2445.90,5163.66)	0.72(0.62,0.83)	31.79(13.65,66.44)	0.72(0.61,0.82)	-	-
Central African Republic	25.56(14.87,43.92)	0.06(-0.04,0.15)	1104.23(749.77,1578.85)	0.05(-0.08,0.18)	9.64(4.05,20.51)	0.07(-0.06,0.19)	-	-
Chad	23.59(13.46,39.72)	0.66(0.48,0.84)	992.59(670.62,1429.00)	0.90(0.65,1.14)	8.47(3.51,17.91)	0.87(0.61,1.12)	-	-
Chile	94.51(55.05,156.75)	1.63(1.33,1.93)	4085.53(2807.86,5913.41)	1.71(1.40,2.03)	36.08(15.29,74.57)	1.70(1.39,2.01)	-	-
China	60.42(35.84,100.32)	1.62(1.47,1.77)	2959.21(2049.56,4213.17)	2.07(1.91,2.24)	25.57(11.12,53.72)	2.09(1.92,2.26)	-	-
Colombia	63.40(41.16,98.88)	0.68(0.63,0.73)	4133.29(2800.41,6002.71)	0.79(0.73,0.85)	36.77(15.76,78.64)	0.79(0.74,0.85)	-	-
Comoros	31.96(18.56,55.80)	0.25(0.13,0.38)	1477.86(999.86,2130.61)	0.31(0.14,0.47)	13.17(5.55,27.38)	0.34(0.18,0.50)	-	-
Congo	32.53(18.89,55.67)	0.69(0.58,0.80)	1520.75(1029.12,2200.93)	0.90(0.76,1.04)	12.99(5.54,27.31)	0.88(0.72,1.04)	-	-
Cook Islands	100.32(64.30,166.10)	0.97(0.86,1.09)	5892.50(3998.00,8493.04)	1.12(0.99,1.26)	51.50(22.31,109.37)	1.12(0.99,1.26)	-	-
Costa Rica	73.85(48.68,114.26)	0.70(0.64,0.76)	5173.80(3455.52,7466.21)	0.80(0.74,0.86)	45.23(19.21,97.68)	0.79(0.73,0.86)	-	-
Croatia	8.50(4.75,14.93)	0.91(0.83,0.99)	403.90(262.67,610.13)	1.07(0.97,1.17)	3.53(1.35,7.70)	1.08(0.98,1.18)	-	-
Cuba	54.67(34.34,89.87)	0.78(0.74,0.82)	3268.01(2175.31,4681.63)	0.94(0.89,0.99)	28.88(12.24,61.32)	0.95(0.90,1.00)	-	-
Cyprus	110.23(70.38,178.43)	1.19(1.03,1.35)	6321.90(4326.37,9087.59)	1.31(1.14,1.48)	56.95(25.10,119.36)	1.29(1.13,1.46)	-	-
Czechia	8.31(4.71,14.39)	0.72(0.67,0.77)	396.05(256.97,595.81)	0.78(0.73,0.84)	3.44(1.35,7.25)	0.76(0.70,0.82)	-	-
Côte d'Ivoire	31.85(18.42,54.91)	1.06(0.85,1.27)	1474.26(996.69,2158.14)	1.41(1.13,1.70)	12.71(5.34,27.25)	1.42(1.13,1.71)	-	-
Democratic People's Republic of Korea	41.73(24.88,69.14)	0.26(0.18,0.33)	1882.44(1287.90,2684.85)	0.32(0.23,0.41)	16.28(6.88,34.17)	0.33(0.25,0.42)	-	-
Democratic Republic of the Congo	27.71(15.90,47.31)	0.91(0.79,1.04)	1218.93(825.61,1755.55)	1.19(1.02,1.35)	10.53(4.46,22.12)	1.18(0.99,1.36)	-	-
Denmark	110.57(71.49,177.38)	0.70(0.60,0.80)	6227.72(4296.80,8781.31)	0.79(0.66,0.92)	56.46(25.01,117.40)	0.79(0.66,0.92)	-	-
Djibouti	35.14(20.04,60.42)	1.38(1.28,1.49)	1675.39(1122.87,2456.37)	1.81(1.67,1.96)	14.51(6.10,31.39)	1.83(1.69,1.97)	-	-
Dominica	52.29(32.63,83.86)	0.73(0.64,0.82)	3067.91(2045.04,4446.14)	0.86(0.74,0.98)	27.17(11.62,58.62)	0.85(0.73,0.97)	-	-

Table S5 The ASIR, ASPR,and ASDR of PCOS in 204 countries and territories in 2021,and the EAPC from 1990 to 2021. (continued)

location_name	SAIR (1/100,000, 95%UI)	EAPC ASIR (EAPC, 95% CI)	SAPR (1/100,000, 95%UI)	EAPC SAPR (EAPC, 95% CI)	SADR (1/100,000, 95%UI)	EAPC SADR (EAPC, 95% CI)	SAMR	EAPC SAMR
Dominican Republic	52.92(33.23,86.37)	1.20(1.14,1.27)	3092.81(2061.25,4513.28)	1.55(1.46,1.64)	27.21(11.43,58.28)	1.53(1.44,1.63)	-	-
Ecuador	105.59(72.04,168.19)	0.62(0.36,0.88)	7052.64(4906.80,9805.99)	0.88(0.64,1.11)	61.21(26.04,128.17)	0.85(0.61,1.08)	-	-
Egypt	77.44(48.72,128.96)	0.59(0.54,0.63)	4406.99(3040.68,6300.09)	0.63(0.56,0.69)	39.46(17.33,82.55)	0.52(0.45,0.60)	-	-
El Salvador	67.42(44.59,102.74)	0.85(0.73,0.97)	4530.90(3076.02,6574.04)	1.06(0.95,1.16)	39.45(16.90,84.34)	1.04(0.93,1.14)	-	-
Equatorial Guinea	41.30(24.64,70.74)	2.22(1.89,2.55)	2089.87(1404.15,2997.69)	2.98(2.53,3.43)	17.99(7.57,37.44)	3.00(2.54,3.45)	-	-
Eritrea	25.82(14.73,43.61)	0.95(0.81,1.09)	1107.49(743.84,1594.10)	1.27(1.06,1.47)	9.67(3.94,20.46)	1.26(1.07,1.46)	-	-
Estonia	13.72(7.53,24.45)	1.08(1.01,1.15)	577.43(380.94,863.94)	1.42(1.33,1.51)	5.14(2.04,11.27)	1.41(1.32,1.50)	-	-
Eswatini	43.34(25.75,73.82)	0.34(0.15,0.53)	2145.53(1459.37,3042.39)	0.42(0.17,0.68)	18.68(7.77,39.09)	0.38(0.13,0.63)	-	-
Ethiopia	28.74(16.43,49.09)	1.08(1.03,1.14)	1142.60(792.13,1639.21)	1.56(1.50,1.63)	9.95(4.23,21.14)	1.55(1.49,1.61)	-	-
Fiji	84.16(51.97,138.16)	1.07(0.97,1.18)	4688.94(3216.32,6714.67)	1.24(1.09,1.38)	41.37(17.87,86.22)	1.24(1.09,1.38)	-	-
Finland	111.03(70.83,177.86)	0.67(0.61,0.73)	6342.16(4357.37,8968.38)	0.70(0.65,0.74)	57.06(25.17,118.08)	0.69(0.65,0.74)	-	-
France	104.92(66.95,168.85)	0.68(0.63,0.73)	6058.46(4126.39,8652.34)	0.70(0.66,0.74)	55.25(24.31,114.71)	0.68(0.64,0.72)	-	-
Gabon	37.86(21.71,66.47)	0.96(0.84,1.07)	1861.45(1233.45,2759.73)	1.18(1.03,1.32)	16.00(6.75,34.06)	1.18(1.04,1.33)	-	-
Gambia	29.75(17.07,50.40)	0.79(0.66,0.92)	1344.74(921.72,1948.59)	1.03(0.86,1.19)	11.60(4.82,24.44)	1.03(0.87,1.19)	-	-
Georgia	26.07(15.73,45.03)	1.80(1.60,2.00)	1379.37(935.32,1946.97)	2.31(2.06,2.56)	12.10(5.07,25.27)	2.30(2.05,2.55)	-	-
Germany	105.75(67.42,168.06)	0.63(0.58,0.68)	5882.79(4096.09,8269.39)	0.65(0.59,0.70)	52.57(23.35,108.20)	0.60(0.55,0.65)	-	-
Ghana	30.60(17.48,52.26)	0.72(0.54,0.91)	1395.60(952.90,2018.73)	0.93(0.68,1.18)	12.01(5.09,24.95)	0.88(0.64,1.14)	-	-
Greece	117.81(75.95,185.52)	0.54(0.38,0.70)	6886.58(4721.70,9802.15)	0.52(0.34,0.70)	61.67(27.13,130.22)	0.51(0.33,0.69)	-	-
Greenland	78.03(45.80,127.75)	1.02(0.91,1.13)	3139.38(2180.13,4494.44)	0.93(0.84,1.02)	28.07(12.19,58.67)	0.93(0.85,1.02)	-	-
Grenada	48.39(30.75,78.20)	0.83(0.73,0.93)	2764.78(1849.60,3975.98)	1.00(0.87,1.14)	24.49(10.60,53.26)	1.00(0.87,1.13)	-	-
Guam	102.07(63.77,172.59)	0.95(0.87,1.03)	5967.62(4081.27,8467.62)	1.11(1.02,1.20)	52.35(22.68,109.82)	1.11(1.01,1.20)	-	-
Guatemala	59.40(38.87,90.96)	0.83(0.73,0.93)	3904.87(2576.25,5719.42)	0.96(0.84,1.07)	33.45(14.08,71.17)	0.97(0.85,1.08)	-	-
Guinea	27.92(16.06,47.24)	0.86(0.75,0.96)	1235.53(847.10,1787.25)	1.11(0.97,1.25)	10.60(4.47,23.01)	1.09(0.94,1.24)	-	-
Guinea-Bissau	27.99(15.99,48.03)	0.78(0.58,0.98)	1233.64(834.36,1780.11)	1.05(0.78,1.32)	10.65(4.49,22.50)	1.05(0.79,1.33)	-	-
Guyana	49.78(31.10,80.85)	0.95(0.87,1.03)	2886.18(1931.13,4151.73)	1.17(1.07,1.27)	25.37(10.80,54.24)	1.18(1.06,1.30)	-	-
Haiti	36.26(22.41,58.69)	0.69(0.66,0.73)	1862.16(1261.06,2655.67)	0.83(0.78,0.88)	16.06(6.81,34.43)	0.77(0.71,0.83)	-	-
Honduras	59.99(39.29,93.81)	1.06(0.95,1.18)	3942.76(2672.75,5757.31)	1.27(1.16,1.38)	34.01(14.58,72.73)	1.24(1.12,1.36)	-	-
Hungary	8.53(4.83,14.91)	0.64(0.58,0.70)	410.28(266.44,622.51)	0.68(0.60,0.75)	3.57(1.43,7.87)	0.68(0.61,0.75)	-	-
Iceland	120.09(77.50,194.92)	0.74(0.67,0.82)	7109.79(4926.57,10120.19)	0.74(0.69,0.79)	63.80(28.29,133.01)	0.73(0.67,0.78)	-	-

Table S5 The ASIR, ASPR,and ASDR of PCOS in 204 countries and territories in 2021,and the EAPC from 1990 to 2021. (continued)

location_name	SAIR (1/100,000, 95%UI)	EAPC ASIR (EAPC, 95% CI)	SAPR (1/100,000, 95%UI)	EAPC SAPR (EAPC, 95% CI)	SADR (1/100,000, 95%UI)	EAPC SADR (EAPC, 95% CI)	SAMR	EAPC SAMR
India	46.94(27.96,77.04)	1.78(1.66,1.91)	2429.87(1713.86,3409.12)	2.35(2.20,2.50)	21.22(9.14,44.45)	2.29(2.14,2.43)	-	-
Indonesia	112.78(68.28,183.76)	1.94(1.84,2.03)	5510.68(3855.35,7827.70)	2.37(2.28,2.46)	48.69(21.32,101.59)	2.32(2.24,2.40)	-	-
Iran (Islamic Republic of)	79.60(48.98,132.07)	1.16(0.87,1.45)	4240.11(2933.54,6070.59)	1.37(1.01,1.73)	38.99(16.98,81.40)	1.39(1.03,1.75)	-	-
Iraq	70.58(44.09,117.13)	0.56(0.46,0.65)	3881.34(2644.00,5618.46)	0.61(0.49,0.73)	34.14(14.68,71.71)	0.60(0.48,0.72)	-	-
Ireland	113.86(72.73,184.92)	0.66(0.57,0.74)	6587.16(4579.85,9384.99)	0.66(0.56,0.75)	59.04(25.76,123.14)	0.64(0.55,0.74)	-	-
Israel	106.66(67.70,173.92)	0.73(0.64,0.82)	6012.38(4148.61,8535.26)	0.73(0.65,0.82)	53.71(23.28,111.28)	0.72(0.63,0.80)	-	-
Italy	201.75(143.26,289.41)	-0.56(-0.70,-0.42)	15307.74(10698.08,21343.59)	-0.42(-0.54,-0.29)	135.48(59.84,288.85)	-0.37(-0.49,-0.25)	-	-
Jamaica	50.16(30.60,83.01)	0.80(0.75,0.86)	2922.68(1965.20,4190.79)	0.97(0.89,1.04)	25.91(10.97,55.07)	0.96(0.89,1.04)	-	-
Japan	360.92(199.08,573.59)	0.62(0.48,0.75)	12234.24(8601.85,17244.66)	0.21(0.16,0.26)	106.56(47.24,217.76)	0.21(0.16,0.26)	-	-
Jordan	72.27(44.56,119.06)	0.79(0.74,0.84)	3972.24(2737.61,5619.07)	0.87(0.81,0.93)	35.12(15.01,74.71)	0.79(0.70,0.88)	-	-
Kazakhstan	20.32(11.77,35.18)	0.91(0.90,0.93)	992.21(666.36,1419.88)	1.21(1.18,1.25)	8.72(3.64,18.37)	1.19(1.16,1.22)	-	-
Kenya	33.67(19.25,57.74)	0.40(0.32,0.47)	1430.64(983.28,2048.44)	0.53(0.42,0.64)	12.33(5.25,26.07)	0.54(0.43,0.65)	-	-
Kiribati	72.29(45.56,117.40)	0.95(0.77,1.14)	3855.72(2659.56,5435.91)	1.12(0.90,1.34)	33.73(14.64,71.05)	1.12(0.90,1.34)	-	-
Kuwait	87.10(55.23,144.48)	0.70(0.65,0.75)	5169.37(3523.96,7376.48)	0.80(0.74,0.86)	45.70(19.84,96.94)	0.79(0.73,0.85)	-	-
Kyrgyzstan	17.00(9.66,29.28)	0.39(0.32,0.45)	777.95(512.85,1131.53)	0.45(0.37,0.53)	6.80(2.78,14.42)	0.46(0.38,0.53)	-	-
Lao People's Democratic Republic	99.21(58.55,163.36)	1.84(1.78,1.90)	4667.77(3241.32,6691.83)	2.22(2.16,2.28)	41.15(17.86,84.61)	2.22(2.16,2.28)	-	-
Latvia	12.97(7.12,22.81)	0.84(0.79,0.88)	530.92(357.33,771.01)	1.08(1.00,1.15)	4.67(1.87,10.08)	1.05(0.98,1.12)	-	-
Lebanon	77.88(48.72,130.33)	0.77(0.75,0.80)	4456.87(3054.64,6435.80)	0.89(0.87,0.91)	38.89(16.53,81.19)	0.90(0.88,0.91)	-	-
Lesotho	37.64(22.04,63.31)	0.98(0.88,1.08)	1789.18(1214.95,2601.78)	1.25(1.12,1.38)	15.48(6.47,32.93)	1.24(1.11,1.37)	-	-
Liberia	30.02(17.23,51.23)	1.02(0.93,1.11)	1363.87(923.09,1976.70)	1.40(1.27,1.52)	11.55(4.90,24.59)	1.35(1.24,1.46)	-	-
Libya	75.31(46.93,125.50)	0.44(0.40,0.49)	4233.95(2908.67,6049.89)	0.46(0.42,0.51)	37.52(15.88,78.49)	0.47(0.41,0.52)	-	-
Lithuania	12.61(6.85,22.28)	0.99(0.94,1.04)	511.61(342.06,769.97)	1.30(1.21,1.38)	4.49(1.77,9.63)	1.26(1.18,1.34)	-	-
Luxembourg	118.11(76.29,188.49)	0.77(0.70,0.83)	6888.97(4742.87,9785.91)	0.79(0.74,0.85)	61.83(26.62,130.18)	0.79(0.73,0.84)	-	-
Madagascar	25.03(14.17,42.65)	0.40(0.36,0.43)	1064.57(719.94,1536.13)	0.53(0.48,0.58)	9.39(3.97,19.61)	0.57(0.52,0.62)	-	-
Malawi	31.14(17.99,54.35)	0.41(0.33,0.49)	1377.41(938.96,1985.09)	0.44(0.32,0.57)	11.77(5.09,24.55)	0.42(0.30,0.54)	-	-
Malaysia	149.17(92.27,255.09)	1.48(1.34,1.62)	7877.54(5429.38,11194.77)	1.88(1.73,2.04)	68.11(30.00,140.09)	1.88(1.72,2.03)	-	-
Maldives	125.64(75.43,212.02)	2.86(2.61,3.11)	6307.02(4333.53,9061.94)	3.39(3.10,3.67)	55.95(24.13,121.75)	3.39(3.11,3.68)	-	-
Mali	25.89(14.92,43.44)	0.94(0.79,1.10)	1110.53(749.76,1598.46)	1.25(1.04,1.47)	9.58(4.07,20.59)	1.24(1.02,1.47)	-	-

Table S5 The ASIR, ASPR,and ASDR of PCOS in 204 countries and territories in 2021,and the EAPC from 1990 to 2021. (continued)

location_name	SAIR (1/100,000, 95%UI)	EAPC ASIR (EAPC, 95% CI)	SAPR (1/100,000, 95%UI)	EAPC SAPR (EAPC, 95% CI)	SADR (1/100,000, 95%UI)	EAPC SADR (EAPC, 95% CI)	SAMR	EAPC SAMR
Malta	117.29(75.57,190.29)	0.87(0.73,1.01)	6858.63(4688.34,9790.52)	0.91(0.77,1.05)	61.50(26.91,128.68)	0.89(0.75,1.03)	-	-
Marshall Islands	71.66(44.42,119.04)	1.23(1.08,1.38)	3772.05(2564.59,5372.34)	1.47(1.29,1.65)	32.97(14.25,68.85)	1.46(1.28,1.64)	-	-
Mauritania	33.94(19.66,59.00)	0.58(0.44,0.72)	1609.40(1092.70,2313.76)	0.76(0.57,0.96)	14.07(5.90,29.19)	0.76(0.57,0.96)	-	-
Mauritius	139.27(84.76,237.87)	1.28(1.17,1.39)	7204.68(5002.82,10251.44)	1.67(1.54,1.79)	63.06(27.44,132.36)	1.66(1.54,1.77)	-	-
Mexico	93.93(64.87,138.96)	-0.49(-0.71,-0.27)	6800.66(4741.46,9458.27)	-0.53(-0.77,-0.28)	58.71(25.55,125.24)	-0.55(-0.79,-0.30)	-	-
Micronesia (Federated States of)	76.62(47.90,123.87)	0.85(0.68,1.02)	4149.01(2852.97,5923.55)	0.97(0.76,1.17)	36.39(15.65,74.72)	0.96(0.75,1.17)	-	-
Monaco	118.27(76.01,189.87)	0.55(0.48,0.63)	6935.51(4755.86,9790.87)	0.48(0.41,0.55)	62.20(26.99,131.56)	0.46(0.40,0.53)	-	-
Mongolia	18.57(10.79,31.24)	0.96(0.91,1.00)	879.31(589.12,1267.28)	1.25(1.20,1.30)	7.71(3.20,16.66)	1.26(1.21,1.31)	-	-
Montenegro	8.57(4.87,14.78)	0.86(0.80,0.92)	412.15(266.10,613.38)	1.03(0.94,1.11)	3.60(1.42,7.85)	1.04(0.95,1.12)	-	-
Morocco	70.46(43.13,117.30)	0.79(0.77,0.81)	3880.30(2655.36,5631.03)	1.01(0.97,1.05)	34.57(15.19,72.13)	0.96(0.92,1.00)	-	-
Mozambique	30.08(17.08,52.25)	1.01(0.95,1.07)	1364.10(918.53,1959.68)	1.33(1.25,1.42)	11.64(4.90,24.38)	1.32(1.23,1.42)	-	-
Myanmar	101.12(61.28,165.67)	2.34(2.20,2.48)	4766.35(3308.13,6713.01)	2.76(2.62,2.90)	42.16(18.26,88.43)	2.77(2.63,2.91)	-	-
Namibia	36.30(21.33,61.32)	0.72(0.62,0.82)	1693.89(1164.20,2446.55)	0.92(0.79,1.05)	14.74(6.37,30.82)	0.88(0.73,1.02)	-	-
Nauru	86.56(53.94,144.39)	0.91(0.85,0.96)	4827.54(3310.93,7022.42)	1.06(0.99,1.12)	42.19(18.51,88.37)	1.06(0.99,1.12)	-	-
Nepal	29.75(17.28,49.78)	1.50(1.46,1.55)	1255.47(841.66,1783.34)	1.81(1.76,1.86)	11.26(4.71,23.55)	1.80(1.76,1.84)	-	-
Netherlands	105.73(66.46,170.12)	0.71(0.66,0.77)	6001.76(4119.29,8576.83)	0.70(0.65,0.74)	53.97(23.38,113.86)	0.68(0.63,0.73)	-	-
New Zealand	233.96(140.65,397.33)	-0.04(-0.24,0.17)	10847.39(7646.21,15149.89)	-0.10(-0.22,0.01)	94.60(42.45,198.63)	-0.09(-0.20,0.02)	-	-
Nicaragua	63.70(41.86,96.84)	0.89(0.80,0.97)	4240.35(2822.03,6077.79)	1.05(0.96,1.14)	36.92(15.91,79.53)	1.03(0.94,1.13)	-	-
Niger	23.47(13.51,39.77)	0.68(0.57,0.79)	992.13(671.34,1431.49)	0.91(0.76,1.05)	8.57(3.55,18.19)	0.83(0.69,0.97)	-	-
Nigeria	33.14(19.06,56.53)	0.51(0.39,0.63)	1418.26(972.87,2023.71)	0.74(0.56,0.91)	12.28(5.20,26.28)	0.76(0.58,0.94)	-	-
Niue	95.33(60.59,158.42)	1.06(0.92,1.19)	5514.80(3767.48,7955.70)	1.26(1.10,1.43)	48.28(20.77,100.07)	1.26(1.10,1.42)	-	-
North Macedonia	8.03(4.46,14.04)	0.96(0.90,1.02)	373.65(241.17,565.59)	1.16(1.09,1.24)	3.25(1.28,7.10)	1.16(1.08,1.23)	-	-
Northern Mariana Islands	91.91(57.73,153.33)	0.61(0.48,0.74)	5245.34(3565.21,7441.85)	0.66(0.50,0.82)	46.00(19.92,97.06)	0.66(0.50,0.82)	-	-
Norway	110.44(69.59,183.02)	0.34(0.23,0.44)	6138.26(4261.95,8699.41)	0.32(0.22,0.43)	55.08(24.17,114.09)	0.32(0.22,0.43)	-	-
Oman	80.03(49.81,130.68)	1.42(1.34,1.49)	4600.78(3124.43,6595.14)	1.74(1.65,1.82)	40.53(17.35,85.34)	1.74(1.65,1.83)	-	-
Pakistan	35.54(20.15,60.91)	0.59(0.50,0.67)	1468.96(1000.91,2097.27)	0.54(0.39,0.69)	13.26(5.63,28.21)	0.55(0.38,0.73)	-	-

Table S5 The ASIR, ASPR,and ASDR of PCOS in 204 countries and territories in 2021,and the EAPC from 1990 to 2021. (continued)

location_name	SAIR (1/100,000, 95%UI)	EAPC ASIR (EAPC, 95% CI)	SAPR (1/100,000, 95%UI)	EAPC SAPR (EAPC, 95% CI)	SADR (1/100,000, 95%UI)	EAPC SADR (EAPC, 95% CI)	SAMR	EAPC SAMR
Palau	95.35(59.85,160.62)	0.89(0.76,1.02)	5501.51(3751.82,7922.00)	1.04(0.87,1.20)	48.13(20.79,99.42)	1.03(0.87,1.20)	-	-
Palestine	68.27(42.15,111.74)	0.66(0.62,0.70)	3713.23(2529.61,5322.37)	0.75(0.70,0.80)	32.75(14.10,69.41)	0.75(0.70,0.80)	-	-
Panama	65.13(42.34,102.21)	1.13(1.06,1.20)	4403.74(2963.54,6461.78)	1.36(1.27,1.45)	38.02(16.33,83.14)	1.35(1.25,1.44)	-	-
Papua New Guinea	61.91(38.22,101.31)	0.86(0.72,1.00)	3128.83(2137.81,4505.22)	1.00(0.83,1.17)	27.12(11.58,56.70)	1.01(0.83,1.19)	-	-
Paraguay	23.29(13.68,40.38)	1.33(1.29,1.38)	1145.87(758.91,1687.55)	1.67(1.61,1.73)	10.07(4.12,21.58)	1.65(1.59,1.71)	-	-
Peru	93.18(59.70,148.57)	0.78(0.75,0.82)	6134.63(4150.05,8983.03)	1.24(1.20,1.28)	52.84(22.84,112.75)	1.22(1.18,1.26)	-	-
Philippines	111.16(66.27,182.90)	1.62(1.53,1.71)	5366.33(3733.00,7654.25)	2.17(2.02,2.31)	47.69(20.33,99.66)	2.15(2.02,2.29)	-	-
Poland	9.66(5.95,15.54)	-0.91(-1.16,-0.66)	513.10(358.28,702.95)	-0.01(-0.08,0.06)	4.50(1.96,9.42)	-0.01(-0.08,0.05)	-	-
Portugal	109.00(68.92,175.41)	0.73(0.57,0.89)	6230.59(4257.31,8850.11)	0.74(0.55,0.92)	55.90(24.75,115.75)	0.73(0.55,0.91)	-	-
Puerto Rico	67.29(43.27,107.22)	0.69(0.61,0.76)	4303.67(2827.34,6248.61)	0.82(0.74,0.90)	38.06(16.35,79.85)	0.82(0.74,0.90)	-	-
Qatar	86.38(54.06,144.52)	0.51(0.49,0.54)	5075.53(3448.38,7343.35)	0.54(0.50,0.58)	44.75(19.17,92.82)	0.53(0.49,0.57)	-	-
Republic of Korea	185.19(105.04,293.32)	1.12(0.75,1.48)	6038.89(4131.52,8659.97)	1.11(0.77,1.45)	52.77(23.51,109.05)	1.10(0.76,1.44)	-	-
Republic of Moldova	11.83(6.47,20.13)	1.15(1.04,1.26)	472.95(315.19,696.08)	1.52(1.37,1.67)	4.22(1.70,9.37)	1.49(1.36,1.62)	-	-
Romania	8.37(4.64,14.54)	1.03(0.98,1.08)	396.30(259.51,600.38)	1.24(1.19,1.29)	3.46(1.34,7.60)	1.24(1.19,1.30)	-	-
Russian Federation	14.66(8.03,25.12)	0.75(0.72,0.78)	523.36(356.35,766.63)	1.00(0.96,1.04)	4.63(1.91,9.76)	1.00(0.95,1.04)	-	-
Rwanda	30.64(17.43,52.06)	0.66(0.62,0.71)	1377.45(933.23,2008.44)	0.81(0.75,0.88)	11.92(5.01,25.24)	0.78(0.71,0.86)	-	-
Saint Kitts and Nevis	57.04(36.44,91.88)	0.76(0.70,0.82)	3476.08(2335.04,5070.45)	0.90(0.81,0.98)	30.75(13.24,64.52)	0.90(0.82,0.98)	-	-
Saint Lucia	49.50(30.46,81.81)	0.60(0.49,0.70)	2854.98(1910.16,4013.40)	0.68(0.54,0.82)	25.26(10.81,54.00)	0.67(0.53,0.82)	-	-
Saint Vincent and the Grenadines	50.39(31.24,81.83)	1.11(1.02,1.20)	2926.26(1954.56,4221.93)	1.37(1.27,1.47)	25.92(11.11,55.88)	1.36(1.26,1.46)	-	-
Samoa	83.73(52.31,142.55)	0.75(0.64,0.86)	4636.23(3169.76,6647.75)	0.86(0.73,0.99)	40.57(17.64,86.43)	0.86(0.73,0.99)	-	-
San Marino	115.08(73.06,184.35)	0.56(0.48,0.64)	6704.95(4569.41,9440.71)	0.52(0.43,0.60)	60.13(26.05,127.65)	0.50(0.42,0.59)	-	-
Sao Tome and Principe	31.75(18.20,54.41)	0.79(0.64,0.94)	1447.41(979.79,2092.55)	1.05(0.84,1.25)	12.49(5.28,26.39)	1.05(0.84,1.27)	-	-
Saudi Arabia	85.28(53.44,144.32)	1.02(0.99,1.04)	4975.37(3389.93,7197.71)	1.22(1.19,1.24)	43.86(19.40,92.77)	1.21(1.19,1.23)	-	-
Senegal	29.61(16.87,51.39)	0.36(0.27,0.45)	1325.73(897.11,1907.21)	0.52(0.39,0.64)	11.55(4.86,24.57)	0.51(0.39,0.64)	-	-
Serbia	8.06(4.46,14.21)	0.94(0.89,1.00)	377.84(244.71,554.14)	1.11(1.05,1.18)	3.31(1.29,7.29)	1.11(1.05,1.17)	-	-
Seychelles	132.81(81.26,220.14)	0.85(0.79,0.91)	6738.58(4663.83,9572.58)	1.08(1.02,1.14)	59.28(25.63,123.37)	1.07(1.01,1.13)	-	-
Sierra Leone	29.98(17.33,51.95)	1.15(1.04,1.25)	1366.94(922.07,1999.28)	1.53(1.39,1.67)	11.66(4.87,24.77)	1.51(1.36,1.66)	-	-
Singapore	219.56(122.83,349.57)	1.58(1.43,1.73)	7156.57(4937.48,10277.61)	1.54(1.39,1.69)	62.48(28.20,130.21)	1.53(1.38,1.68)	-	-

Table S5 The ASIR, ASPR,and ASDR of PCOS in 204 countries and territories in 2021,and the EAPC from 1990 to 2021. (continued)

location_name	SAIR (1/100,000, 95%UI)	EAPC ASIR (EAPC, 95% CI)	SAPR (1/100,000, 95%UI)	EAPC SAPR (EAPC, 95% CI)	SADR (1/100,000, 95%UI)	EAPC SADR (EAPC, 95% CI)	SAMR	EAPC SAMR
Slovakia	8.41(4.72,14.59)	0.93(0.89,0.97)	399.95(262.18,601.29)	1.07(1.03,1.12)	3.49(1.38,7.81)	1.08(1.04,1.12)	-	-
Slovenia	8.81(4.96,15.37)	0.93(0.85,1.02)	426.34(275.90,633.90)	1.16(1.05,1.27)	3.66(1.47,7.92)	1.14(1.03,1.25)	-	-
Solomon Islands	67.47(41.48,112.33)	1.04(0.84,1.24)	3492.48(2370.50,4963.72)	1.23(0.98,1.47)	30.58(13.20,65.47)	1.23(0.98,1.48)	-	-
Somalia	25.14(14.36,42.75)	0.47(0.43,0.50)	1068.11(726.60,1564.60)	0.59(0.55,0.64)	9.25(3.79,19.50)	0.62(0.57,0.67)	-	-
South Africa	48.64(28.46,82.77)	0.71(0.66,0.76)	2254.18(1541.10,3239.34)	0.93(0.85,1.00)	19.57(8.29,41.51)	0.89(0.82,0.96)	-	-
South Sudan	26.10(14.72,44.62)	0.15(0.06,0.23)	1124.47(761.63,1631.09)	0.18(0.07,0.29)	9.76(4.08,20.91)	0.20(0.09,0.30)	-	-
Spain	112.26(71.36,182.74)	0.59(0.48,0.71)	6294.89(4337.06,8876.96)	0.66(0.53,0.80)	55.87(24.56,117.91)	0.63(0.50,0.76)	-	-
Sri Lanka	122.93(72.77,207.02)	1.52(1.29,1.75)	6055.35(4172.08,8579.01)	1.86(1.64,2.08)	53.25(23.15,110.06)	1.83(1.62,2.05)	-	-
Sudan	64.40(39.77,107.04)	1.59(1.47,1.72)	3477.76(2400.42,4977.00)	1.95(1.80,2.11)	29.99(13.10,63.17)	1.93(1.76,2.09)	-	-
Suriname	52.00(32.15,86.52)	0.80(0.77,0.84)	3032.21(2025.34,4365.82)	0.99(0.95,1.03)	26.73(11.50,57.49)	0.99(0.95,1.03)	-	-
Sweden	93.33(57.41,152.16)	0.52(0.31,0.74)	4980.61(3411.68,7086.13)	0.49(0.23,0.74)	44.65(19.60,92.67)	0.48(0.22,0.73)	-	-
Switzerland	105.19(66.15,173.57)	0.48(0.44,0.51)	5959.38(4110.32,8488.14)	0.41(0.40,0.43)	53.42(22.77,110.24)	0.41(0.39,0.42)	-	-
Syrian Arab Republic	70.69(43.22,118.53)	0.83(0.76,0.90)	3873.00(2641.05,5594.09)	0.99(0.91,1.07)	33.89(14.66,71.57)	0.98(0.90,1.07)	-	-
Taiwan (Province of China)	87.39(54.74,146.99)	1.46(1.39,1.53)	4753.67(3344.72,6536.85)	1.78(1.70,1.86)	41.06(17.78,83.72)	1.78(1.70,1.86)	-	-
Tajikistan	15.27(8.61,26.14)	0.73(0.67,0.79)	665.58(437.87,963.57)	0.90(0.83,0.96)	5.94(2.38,12.91)	0.93(0.86,1.00)	-	-
Thailand	131.58(81.30,222.71)	1.75(1.51,1.98)	6659.42(4637.85,9552.96)	2.24(2.08,2.40)	59.34(26.04,125.05)	2.20(2.05,2.35)	-	-
Timor-Leste	86.01(51.86,141.56)	1.64(1.53,1.76)	3938.39(2731.85,5700.70)	1.96(1.85,2.07)	34.96(14.89,72.13)	1.97(1.86,2.08)	-	-
Togo	28.81(16.51,49.31)	0.81(0.68,0.93)	1283.46(868.74,1865.83)	1.09(0.92,1.26)	11.14(4.67,23.85)	1.10(0.93,1.27)	-	-
Tokelau	88.65(55.48,147.66)	1.24(1.12,1.36)	4988.32(3403.03,7139.72)	1.49(1.34,1.64)	43.62(19.36,90.89)	1.48(1.34,1.63)	-	-
Tonga	92.83(58.05,152.50)	0.64(0.47,0.81)	5318.19(3604.59,7677.86)	0.69(0.48,0.91)	46.50(19.90,96.81)	0.69(0.48,0.90)	-	-
Trinidad and Tobago	54.49(34.06,89.03)	1.05(0.94,1.16)	3245.88(2164.83,4673.03)	1.32(1.17,1.46)	28.70(12.25,60.07)	1.29(1.15,1.44)	-	-
Tunisia	71.81(44.91,120.34)	1.03(0.99,1.07)	3978.43(2736.75,5675.02)	1.21(1.17,1.26)	35.39(15.40,74.51)	1.19(1.14,1.24)	-	-
Turkmenistan	19.64(11.18,34.03)	0.95(0.92,0.98)	953.55(638.74,1392.89)	1.26(1.23,1.28)	8.33(3.44,18.00)	1.25(1.23,1.28)	-	-
Tuvalu	82.46(50.90,137.76)	1.09(0.95,1.24)	4502.03(3084.80,6459.78)	1.26(1.08,1.44)	39.38(17.55,83.33)	1.28(1.11,1.46)	-	-
Turkey	68.55(42.01,113.39)	1.01(0.96,1.06)	3735.04(2559.60,5317.71)	1.18(1.12,1.24)	33.01(14.31,69.52)	1.06(0.99,1.14)	-	-
Uganda	29.21(16.62,49.53)	0.56(0.54,0.58)	1287.88(881.20,1849.69)	0.72(0.69,0.76)	11.06(4.62,23.21)	0.67(0.62,0.72)	-	-
Ukraine	13.32(7.22,23.30)	0.62(0.57,0.67)	454.13(301.85,671.71)	0.79(0.72,0.87)	4.08(1.66,8.74)	0.77(0.70,0.85)	-	-
United Arab Emirates	80.37(50.51,135.36)	0.75(0.66,0.84)	4644.27(3183.76,6775.54)	0.85(0.73,0.96)	40.86(17.76,85.53)	0.84(0.72,0.96)	-	-

Table S5 The ASIR, ASPR,and ASDR of PCOS in 204 countries and territories in 2021,and the EAPC from 1990 to 2021. (continued)

location_name	SAIR (1/100,000, 95%UI)	EAPC ASIR (EAPC, 95% CI)	SAPR (1/100,000, 95%UI)	EAPC SAPR (EAPC, 95% CI)	SADR (1/100,000, 95%UI)	EAPC SADR (EAPC, 95% CI)	SAMR	EAPC SAMR
United Kingdom	127.51(81.45,207.41)	0.51(0.41,0.62)	7306.96(5077.77,10369.41)	0.48(0.35,0.61)	65.39(28.94,136.60)	0.47(0.34,0.60)	-	-
United Republic of Tanzania	32.12(18.45,55.28)	0.52(0.44,0.59)	1467.31(988.75,2110.59)	0.63(0.54,0.72)	12.52(5.47,26.39)	0.62(0.53,0.70)	-	-
United States of America	197.42(115.84,310.40)	-0.33(-1.03,0.38)	7599.79(5617.03,10130.88)	-0.58(-1.12,-0.04)	67.35(30.78,134.59)	-0.60(-1.13,-0.06)	-	-
United States Virgin Islands	65.29(42.21,106.13)	0.64(0.55,0.72)	4128.47(2737.71,5958.45)	0.71(0.61,0.82)	36.50(15.64,77.43)	0.70(0.60,0.81)	-	-
Uruguay	86.89(50.67,144.13)	1.53(1.36,1.71)	3781.93(2630.86,5514.93)	1.61(1.42,1.81)	33.46(14.21,69.13)	1.60(1.40,1.79)	-	-
Uzbekistan	19.49(11.23,33.10)	0.88(0.77,0.99)	938.68(622.54,1328.35)	1.15(1.02,1.28)	8.20(3.32,17.77)	1.12(0.99,1.25)	-	-
Vanuatu	72.21(45.26,118.64)	1.03(0.98,1.08)	3834.48(2608.78,5565.57)	1.21(1.16,1.27)	33.61(14.55,71.45)	1.21(1.15,1.26)	-	-
Venezuela (Bolivarian Republic of)	69.62(46.49,106.76)	0.61(0.56,0.66)	4777.65(3214.71,6961.74)	0.67(0.60,0.74)	41.67(17.87,88.19)	0.66(0.59,0.73)	-	-
Viet Nam	94.98(56.60,152.26)	2.36(2.19,2.53)	4371.36(3018.74,6229.93)	2.80(2.61,2.98)	38.41(16.57,80.56)	2.76(2.58,2.94)	-	-
Yemen	49.86(30.84,82.15)	0.86(0.76,0.97)	2500.45(1690.99,3610.80)	0.99(0.86,1.12)	21.97(9.44,46.82)	0.99(0.86,1.12)	-	-
Zambia	35.60(20.13,61.34)	0.43(0.39,0.46)	1691.37(1132.23,2455.00)	0.51(0.46,0.56)	14.54(6.09,30.71)	0.47(0.38,0.55)	-	-
Zimbabwe	33.89(20.30,57.46)	-0.05(-0.19,0.09)	1575.34(1069.09,2247.71)	-0.12(-0.31,0.06)	13.55(5.71,28.93)	-0.15(-0.34,0.05)	-	-

PCOS polycystic ovary syndrome, DALYs disability-adjusted life-years, ASIR age-standardized incidence rate, ASPR age-standardized prevalence rate, ASMR age-standardized mortality rate, ASDR age-standardized DALYs rate, EAPC estimated annual percentage change, CI confdence interval, UI uncertainty intervals

Table S6 The ASIR, ASPR, ASDR and ASMR of Uterine cancer in 204 countries and territories in 2021,and the EAPC from 1990 to 2021.

location_name	SAIR (1/100,000, 95%UI)	EAPC ASIR (EAPC, 95% CI)	SAPR (1/100,000, 95%UI)	EAPC SAPR (EAPC, 95% CI)	SADR (1/100,000, 95%UI)	EAPC SADR (EAPC, 95% CI)	SAMR (1/100,000, 95%UI)	EAPC SAMR (EAPC, 95% CI)
Afghanistan	2.46(0.96,5.01)	2.26(1.92,2.60)	19.74(7.72,40.16)	2.38(2.04,2.74)	31.11(12.16,61.79)	1.13(0.94,1.31)	0.75(0.29,1.48)	1.08(0.89,1.26)
Albania	3.00(1.69,4.88)	1.90(1.51,2.28)	25.09(14.15,40.67)	2.05(1.65,2.45)	16.40(9.30,26.55)	-0.64(-0.85,-0.42)	0.37(0.21,0.61)	-0.78(-1.00,-0.57)
Algeria	0.77(0.41,1.27)	1.85(1.77,1.94)	6.45(3.48,10.68)	1.93(1.85,2.01)	4.34(2.42,7.18)	-0.09(-0.16,-0.01)	0.10(0.06,0.16)	-0.21(-0.29,-0.14)
American Samoa	7.02(2.80,14.84)	1.88(1.79,1.96)	57.12(22.80,120.75)	1.87(1.79,1.96)	63.21(25.61,134.58)	1.70(1.60,1.79)	1.51(0.62,3.19)	1.68(1.58,1.77)
Andorra	2.51(1.27,4.33)	0.10(-0.15,0.36)	21.22(10.77,36.63)	0.13(-0.13,0.39)	7.98(3.95,13.31)	-0.75(-0.93,-0.58)	0.17(0.09,0.28)	-0.91(-1.08,-0.73)
Angola	0.93(0.44,1.67)	0.66(0.44,0.89)	7.17(3.36,13.00)	0.84(0.61,1.08)	14.22(6.65,25.78)	-0.34(-0.49,-0.18)	0.35(0.16,0.63)	-0.39(-0.54,-0.24)
Antigua and Barbuda	4.77(3.92,5.74)	1.18(0.90,1.46)	36.95(30.40,44.60)	1.18(0.90,1.46)	41.33(35.56,48.14)	0.68(0.44,0.93)	0.98(0.84,1.14)	0.71(0.45,0.97)
Argentina	1.81(1.33,2.41)	-1.56(-1.87,-1.24)	14.90(10.98,19.78)	-1.49(-1.80,-1.18)	13.83(10.46,18.02)	-2.62(-2.98,-2.26)	0.31(0.24,0.41)	-2.72(-3.09,-2.36)
Armenia	4.44(3.52,5.49)	0.14(-0.42,0.71)	36.72(29.20,45.55)	0.24(-0.32,0.81)	27.82(23.00,33.47)	-1.54(-2.09,-0.98)	0.65(0.54,0.78)	-1.54(-2.09,-1.00)
Australia	3.58(2.70,4.67)	1.43(1.22,1.64)	29.90(22.56,39.09)	1.49(1.28,1.71)	11.37(8.72,14.60)	0.41(0.18,0.64)	0.24(0.19,0.31)	0.25(0.01,0.50)
Austria	4.09(3.02,5.33)	-0.00(-0.18,0.17)	35.02(25.88,45.65)	0.04(-0.13,0.21)	12.06(9.09,15.55)	-1.33(-1.58,-1.07)	0.25(0.19,0.32)	-1.59(-1.86,-1.32)
Azerbaijan	2.71(1.86,4.52)	-0.46(-0.75,-0.18)	22.23(15.26,36.99)	-0.34(-0.62,-0.05)	22.28(15.62,36.72)	-2.02(-2.20,-1.84)	0.52(0.37,0.86)	-2.05(-2.22,-1.88)
Bahamas	7.78(5.56,10.65)	1.06(0.67,1.46)	59.15(42.17,80.78)	1.08(0.69,1.47)	80.80(58.28,108.26)	0.62(0.31,0.92)	1.89(1.37,2.53)	0.64(0.34,0.94)
Bahrain	3.65(1.95,6.29)	2.83(2.49,3.17)	30.91(16.52,53.26)	2.91(2.58,3.25)	16.11(8.91,27.31)	0.44(0.11,0.78)	0.35(0.20,0.60)	0.19(-0.15,0.52)
Bangladesh	0.60(0.26,1.46)	0.48(0.23,0.74)	4.82(2.13,11.76)	0.71(0.47,0.95)	6.44(2.79,15.43)	-1.36(-1.58,-1.15)	0.15(0.07,0.36)	-1.46(-1.66,-1.26)
Barbados	7.68(5.56,10.47)	0.97(0.53,1.40)	59.85(43.22,81.62)	0.98(0.55,1.41)	65.62(48.64,87.28)	0.55(0.15,0.95)	1.53(1.13,2.02)	0.57(0.17,0.98)
Belarus	7.99(5.40,11.29)	1.47(1.07,1.87)	67.06(45.25,94.73)	1.53(1.13,1.93)	30.19(20.70,42.13)	-0.35(-0.69,-0.00)	0.66(0.45,0.91)	-0.59(-0.95,-0.24)
Belgium	3.95(2.94,5.21)	0.22(0.01,0.43)	33.46(24.97,44.14)	0.26(0.05,0.47)	13.53(10.22,17.43)	-0.82(-1.01,-0.62)	0.29(0.22,0.37)	-0.97(-1.17,-0.77)
Belize	5.11(4.05,6.34)	1.30(1.00,1.60)	38.45(30.42,47.81)	1.26(0.97,1.56)	57.19(46.35,69.09)	0.93(0.54,1.32)	1.36(1.09,1.64)	0.92(0.52,1.31)
Benin	0.68(0.36,1.16)	0.49(0.37,0.61)	5.27(2.83,9.06)	0.59(0.47,0.72)	10.19(5.56,17.20)	-0.21(-0.34,-0.08)	0.25(0.13,0.41)	-0.26(-0.39,-0.13)
Bermuda	6.46(4.47,9.01)	0.34(-0.10,0.78)	52.28(36.21,73.02)	0.43(-0.00,0.87)	32.64(23.09,44.20)	-1.08(-1.39,-0.78)	0.73(0.52,0.99)	-1.22(-1.52,-0.91)
Bhutan	0.66(0.29,1.62)	0.81(0.68,0.95)	5.34(2.36,12.97)	1.03(0.91,1.16)	7.50(3.28,17.42)	-0.90(-1.02,-0.78)	0.18(0.08,0.41)	-0.97(-1.09,-0.85)
Bolivia (Plurinational State of)	3.06(1.61,5.31)	0.41(0.27,0.55)	24.01(12.57,41.62)	0.61(0.47,0.74)	37.74(20.23,62.96)	-1.00(-1.15,-0.86)	0.92(0.49,1.52)	-1.03(-1.18,-0.89)
Bosnia and Herzegovina	3.13(1.87,4.55)	2.13(1.68,2.58)	26.12(15.60,37.90)	2.25(1.78,2.71)	17.00(10.15,24.06)	-0.04(-0.31,0.23)	0.39(0.23,0.55)	-0.17(-0.44,0.09)
Botswana	0.99(0.44,2.02)	1.40(0.73,2.08)	7.63(3.41,15.55)	1.54(0.77,2.31)	12.79(5.77,26.28)	0.57(-0.01,1.15)	0.31(0.14,0.63)	0.50(-0.07,1.08)

Table S6 The ASIR, ASPR, ASDR and ASMR of Uterine cancer in 204 countries and territories in 2021,and the EAPC from 1990 to 2021. (continued)

location_name	SAIR (1/100,000, 95%UI)	EAPC ASIR (EAPC, 95% CI)	SAPR (1/100,000, 95%UI)	EAPC SAPR (EAPC, 95% CI)	SADR (1/100,000, 95%UI)	EAPC SADR (EAPC, 95% CI)	SAMR (1/100,000, 95%UI)	EAPC SAMR (EAPC, 95% CI)
Brazil	1.94(1.75,2.13)	0.60(0.38,0.82)	15.59(14.05,17.21)	0.71(0.49,0.93)	17.26(15.77,18.90)	-0.55(-0.73,-0.37)	0.41(0.38,0.45)	-0.65(-0.83,-0.48)
Brunei Darussalam	3.00(1.80,4.67)	1.93(1.53,2.33)	24.54(14.71,38.31)	2.02(1.63,2.41)	29.18(17.79,45.45)	0.45(-0.06,0.97)	0.70(0.43,1.09)	0.43(-0.11,0.97)
Bulgaria	11.16(8.24,14.63)	2.04(1.36,2.73)	91.70(67.57,120.48)	2.09(1.40,2.78)	65.52(49.89,83.71)	1.07(0.47,1.68)	1.51(1.15,1.91)	0.99(0.40,1.59)
Burkina Faso	0.63(0.33,1.11)	0.23(0.14,0.32)	4.89(2.58,8.65)	0.38(0.29,0.47)	10.16(5.35,17.86)	-0.26(-0.40,-0.13)	0.25(0.13,0.43)	-0.29(-0.42,-0.16)
Burundi	0.83(0.41,1.56)	-1.11(-1.31,-0.90)	6.34(3.10,11.98)	-0.83(-1.02,-0.64)	14.62(7.27,27.37)	-1.70(-1.91,-1.50)	0.35(0.18,0.66)	-1.75(-1.96,-1.54)
Cabo Verde	1.03(0.50,2.21)	0.43(0.19,0.68)	8.39(4.04,18.00)	0.54(0.29,0.79)	9.56(4.76,20.62)	-1.08(-1.23,-0.92)	0.23(0.11,0.48)	-1.07(-1.22,-0.91)
Cambodia	3.12(1.60,5.56)	1.25(1.10,1.40)	25.40(13.04,45.31)	1.38(1.23,1.54)	33.94(17.28,60.43)	-0.23(-0.31,-0.15)	0.81(0.41,1.43)	-0.27(-0.35,-0.19)
Cameroon	0.89(0.44,1.54)	0.17(0.04,0.29)	6.88(3.44,11.94)	0.22(0.05,0.38)	12.80(6.55,21.93)	-0.48(-0.54,-0.43)	0.31(0.16,0.53)	-0.52(-0.58,-0.47)
Canada	4.24(3.09,5.64)	0.81(0.54,1.08)	36.10(26.29,47.98)	0.84(0.57,1.11)	15.80(11.85,20.49)	-0.27(-0.64,0.10)	0.34(0.26,0.44)	-0.41(-0.78,-0.03)
Central African Republic	0.80(0.39,1.45)	-0.25(-0.34,-0.16)	5.90(2.84,10.64)	-0.14(-0.24,-0.04)	16.78(8.21,30.24)	-0.44(-0.53,-0.35)	0.42(0.20,0.75)	-0.47(-0.56,-0.37)
Chad	0.59(0.30,1.05)	0.92(0.85,1.00)	4.46(2.31,7.97)	0.98(0.90,1.07)	10.44(5.43,18.51)	0.55(0.46,0.64)	0.26(0.13,0.45)	0.53(0.44,0.63)
Chile	1.65(1.22,2.19)	0.59(0.35,0.83)	13.79(10.15,18.24)	0.70(0.47,0.93)	9.11(6.92,11.70)	-1.47(-1.78,-1.16)	0.21(0.16,0.26)	-1.62(-1.94,-1.30)
China	3.52(2.36,5.01)	0.62(0.21,1.03)	28.36(18.95,40.33)	0.89(0.46,1.32)	20.87(13.99,29.50)	-2.61(-2.98,-2.25)	0.47(0.32,0.66)	-2.81(-3.18,-2.44)
Colombia	2.06(1.43,2.86)	1.46(0.87,2.06)	16.64(11.55,23.13)	1.65(1.06,2.24)	14.77(10.63,19.96)	-0.33(-0.92,0.27)	0.34(0.25,0.46)	-0.46(-1.05,0.14)
Comoros	1.41(0.74,2.47)	-0.11(-0.38,0.16)	11.00(5.81,19.35)	-0.00(-0.27,0.26)	21.29(11.46,37.11)	-0.91(-1.24,-0.58)	0.51(0.28,0.89)	-0.93(-1.25,-0.62)
Congo	1.67(0.83,3.07)	0.96(0.76,1.16)	12.89(6.40,23.77)	1.16(0.96,1.35)	23.59(12.00,41.99)	-0.09(-0.30,0.12)	0.58(0.30,1.03)	-0.15(-0.35,0.06)
Cook Islands	2.79(1.54,4.73)	1.03(0.79,1.26)	23.34(12.88,39.45)	1.08(0.85,1.32)	15.22(8.61,25.60)	-0.29(-0.53,-0.06)	0.35(0.20,0.58)	-0.43(-0.66,-0.20)
Costa Rica	3.73(2.68,5.01)	2.00(1.41,2.59)	30.30(21.75,40.71)	2.08(1.49,2.66)	23.91(17.56,31.40)	0.93(0.34,1.53)	0.55(0.41,0.73)	0.84(0.25,1.42)
Croatia	8.45(6.07,11.28)	1.25(0.70,1.80)	72.39(52.01,96.66)	1.29(0.75,1.84)	23.72(17.33,31.14)	-0.38(-0.92,0.17)	0.49(0.36,0.63)	-0.66(-1.20,-0.12)
Cuba	9.04(6.43,12.31)	0.14(-0.40,0.68)	71.46(50.74,97.06)	0.18(-0.35,0.72)	62.56(46.12,83.45)	-0.41(-0.90,0.08)	1.45(1.07,1.92)	-0.42(-0.91,0.07)
Cyprus	3.08(1.75,5.54)	0.67(0.29,1.05)	26.19(14.93,47.11)	0.73(0.35,1.12)	10.20(5.83,18.19)	-1.52(-1.74,-1.29)	0.22(0.13,0.39)	-1.81(-2.03,-1.60)
Czechia	7.22(5.38,9.48)	0.83(0.65,1.01)	61.28(45.69,80.48)	0.90(0.72,1.07)	28.25(21.47,36.19)	-0.83(-1.14,-0.53)	0.61(0.47,0.77)	-1.12(-1.43,-0.81)
Côte d'Ivoire	0.67(0.34,1.17)	1.67(1.51,1.84)	5.19(2.64,9.08)	1.83(1.64,2.03)	9.46(4.88,16.57)	0.90(0.74,1.05)	0.23(0.12,0.40)	0.84(0.68,0.99)
Democratic People's Republic of Korea	4.79(2.37,8.50)	1.33(1.19,1.46)	39.61(19.54,70.31)	1.38(1.23,1.53)	36.31(18.15,65.13)	0.29(0.23,0.34)	0.84(0.42,1.51)	0.24(0.18,0.30)
Democratic Republic of the Congo	0.86(0.39,1.57)	0.39(0.06,0.72)	6.63(3.03,12.13)	0.53(0.17,0.89)	13.60(6.16,24.85)	-0.26(-0.48,-0.03)	0.33(0.15,0.61)	-0.26(-0.48,-0.04)

Table S6 The ASIR, ASPR, ASDR and ASMR of Uterine cancer in 204 countries and territories in 2021,and the EAPC from 1990 to 2021. (continued)

location_name	SAIR (1/100,000, 95%UI)	EAPC ASIR (EAPC, 95% CI)	SAPR (1/100,000, 95%UI)	EAPC SAPR (EAPC, 95% CI)	SADR (1/100,000, 95%UI)	EAPC SADR (EAPC, 95% CI)	SAMR (1/100,000, 95%UI)	EAPC SAMR (EAPC, 95% CI)
Denmark	3.91(2.88,5.21)	-0.22(-0.44,0.00)	33.36(24.53,44.41)	-0.16(-0.38,0.06)	12.64(9.55,16.37)	-1.79(-1.96,-1.62)	0.27(0.21,0.34)	-2.04(-2.22,-1.87)
Djibouti	1.22(0.54,2.58)	0.56(0.48,0.65)	9.45(4.24,20.07)	0.62(0.52,0.72)	17.30(7.79,35.74)	-0.18(-0.31,-0.04)	0.42(0.19,0.86)	-0.21(-0.35,-0.08)
Dominica	3.71(2.10,6.10)	0.95(0.83,1.07)	27.68(15.59,45.37)	0.92(0.79,1.04)	44.98(26.25,72.80)	1.02(0.95,1.08)	1.07(0.63,1.73)	0.96(0.89,1.02)
Dominican Republic	5.04(2.82,8.04)	1.37(1.22,1.51)	37.84(21.17,60.29)	1.44(1.29,1.58)	60.42(33.71,95.52)	0.92(0.72,1.12)	1.43(0.80,2.26)	0.89(0.69,1.09)
Ecuador	3.69(2.43,5.41)	0.13(-0.58,0.84)	29.63(19.49,43.38)	0.27(-0.44,0.99)	33.04(22.45,47.46)	-1.34(-2.04,-0.64)	0.79(0.53,1.13)	-1.41(-2.12,-0.70)
Egypt	1.54(0.86,2.56)	1.00(0.82,1.19)	12.79(7.17,21.35)	1.09(0.90,1.28)	10.49(5.87,17.40)	-0.67(-0.87,-0.46)	0.24(0.14,0.40)	-0.78(-0.97,-0.58)
El Salvador	2.57(1.69,3.74)	1.76(1.59,1.93)	20.51(13.53,29.93)	1.99(1.82,2.16)	21.51(14.57,30.57)	-0.04(-0.24,0.16)	0.51(0.35,0.72)	-0.15(-0.36,0.05)
Equatorial Guinea	1.45(0.64,2.71)	2.41(2.19,2.63)	11.42(5.07,21.25)	2.65(2.43,2.87)	16.51(7.54,30.28)	0.05(-0.11,0.20)	0.40(0.18,0.72)	-0.07(-0.22,0.08)
Eritrea	1.32(0.67,2.47)	0.29(0.20,0.37)	10.04(5.12,18.76)	0.44(0.35,0.52)	22.69(11.36,42.61)	-0.28(-0.35,-0.22)	0.56(0.28,1.04)	-0.33(-0.39,-0.27)
Estonia	6.66(4.76,9.02)	-0.09(-0.37,0.18)	56.72(40.57,76.72)	-0.02(-0.29,0.26)	21.21(15.16,27.58)	-2.18(-2.50,-1.85)	0.45(0.33,0.58)	-2.51(-2.85,-2.16)
Eswatini	1.43(0.58,2.72)	1.70(1.04,2.36)	10.76(4.37,20.48)	1.59(1.09,2.09)	20.24(8.54,37.81)	1.32(0.53,2.11)	0.50(0.21,0.92)	1.30(0.51,2.10)
Ethiopia	0.71(0.41,1.35)	-0.79(-1.07,-0.52)	5.55(3.16,10.66)	-0.44(-0.70,-0.18)	10.49(6.02,20.05)	-2.21(-2.44,-1.98)	0.25(0.14,0.48)	-2.28(-2.50,-2.05)
Fiji	4.56(2.47,7.88)	0.95(0.71,1.18)	36.85(19.95,63.85)	0.95(0.72,1.19)	49.14(27.14,84.84)	0.75(0.53,0.97)	1.18(0.65,2.04)	0.75(0.52,0.98)
Finland	4.12(3.00,5.50)	0.29(0.02,0.57)	35.12(25.57,46.88)	0.34(0.06,0.62)	14.17(10.55,18.90)	-1.04(-1.20,-0.89)	0.30(0.23,0.39)	-1.27(-1.42,-1.11)
France	3.94(2.88,5.25)	0.84(0.64,1.03)	33.44(24.45,44.52)	0.88(0.69,1.08)	13.05(9.72,17.05)	-0.69(-0.84,-0.53)	0.28(0.21,0.36)	-0.90(-1.06,-0.75)
Gabon	1.43(0.71,2.54)	0.94(0.71,1.18)	11.31(5.60,20.04)	1.05(0.85,1.25)	17.40(8.62,29.89)	-0.21(-0.50,0.09)	0.42(0.21,0.72)	-0.25(-0.55,0.05)
Gambia	0.90(0.46,1.57)	1.42(1.11,1.73)	7.01(3.61,12.24)	1.45(1.14,1.77)	12.63(6.54,22.08)	0.78(0.50,1.07)	0.30(0.16,0.53)	0.75(0.46,1.04)
Georgia	6.83(5.62,8.15)	-1.24(-1.96,-0.51)	56.26(46.26,67.16)	-1.18(-1.90,-0.45)	51.63(44.12,59.19)	-1.74(-2.38,-1.10)	1.20(1.04,1.37)	-1.72(-2.36,-1.07)
Germany	3.17(2.37,4.18)	-0.11(-0.34,0.13)	26.69(19.96,35.23)	-0.06(-0.29,0.17)	10.57(8.04,13.69)	-0.98(-1.28,-0.69)	0.23(0.17,0.28)	-1.13(-1.45,-0.81)
Ghana	1.14(0.56,2.09)	0.82(0.72,0.91)	8.95(4.39,16.48)	0.90(0.82,0.98)	15.26(7.65,28.02)	0.10(-0.00,0.20)	0.36(0.18,0.66)	0.09(-0.01,0.18)
Greece	4.30(3.57,5.16)	1.15(0.94,1.36)	36.40(30.17,43.61)	1.16(0.95,1.37)	15.73(13.56,18.28)	0.70(0.45,0.96)	0.35(0.30,0.39)	0.69(0.41,0.96)
Greenland	0.98(0.52,1.79)	0.01(-0.08,0.10)	8.06(4.23,14.76)	0.12(0.03,0.21)	7.52(4.07,13.66)	-1.35(-1.43,-1.27)	0.18(0.10,0.33)	-1.37(-1.45,-1.28)
Grenada	5.31(3.56,7.51)	0.57(0.42,0.73)	40.20(27.04,56.81)	0.55(0.38,0.72)	57.57(38.99,79.72)	0.12(-0.07,0.32)	1.35(0.92,1.86)	0.17(-0.04,0.37)
Guam	5.18(3.49,7.14)	1.97(1.46,2.49)	42.81(28.83,59.01)	1.96(1.45,2.47)	33.67(23.06,45.23)	1.62(0.97,2.29)	0.79(0.55,1.07)	1.63(0.94,2.34)
Guatemala	2.62(2.07,3.29)	0.45(0.09,0.80)	20.25(15.99,25.52)	0.70(0.36,1.04)	30.32(24.68,36.90)	-0.93(-1.36,-0.50)	0.73(0.59,0.89)	-0.97(-1.39,-0.55)
Guinea	0.84(0.42,1.57)	0.91(0.86,0.97)	6.42(3.20,12.02)	0.99(0.93,1.04)	14.21(7.01,26.48)	0.46(0.40,0.51)	0.34(0.17,0.63)	0.41(0.35,0.46)
Guinea-Bissau	0.99(0.50,1.66)	0.52(0.47,0.56)	7.45(3.77,12.55)	0.62(0.56,0.67)	17.61(9.05,29.79)	-0.11(-0.13,-0.08)	0.43(0.22,0.72)	-0.12(-0.14,-0.09)
Guyana	5.67(3.59,8.42)	1.64(1.07,2.21)	40.91(25.79,60.74)	1.63(1.07,2.19)	84.26(53.66,124.24)	1.41(0.85,1.97)	2.01(1.28,2.95)	1.40(0.85,1.95)

Table S6 The ASIR, ASPR, ASDR and ASMR of Uterine cancer in 204 countries and territories in 2021,and the EAPC from 1990 to 2021. (continued)

location_name	SAIR (1/100,000, 95%UI)	EAPC ASIR (EAPC, 95% CI)	SAPR (1/100,000, 95%UI)	EAPC SAPR (EAPC, 95% CI)	SADR (1/100,000, 95%UI)	EAPC SADR (EAPC, 95% CI)	SAMR (1/100,000, 95%UI)	EAPC SAMR (EAPC, 95% CI)
Haiti	3.46(1.64,6.50)	0.16(0.10,0.22)	23.27(11.02,43.83)	0.23(0.13,0.33)	69.21(33.34,128.90)	-0.11(-0.19,-0.03)	1.67(0.81,3.12)	-0.11(-0.19,-0.03)
Honduras	4.77(2.36,8.41)	0.89(0.67,1.11)	36.42(18.07,64.22)	1.04(0.82,1.26)	60.08(30.61,107.87)	-0.05(-0.24,0.13)	1.47(0.76,2.63)	0.02(-0.16,0.20)
Hungary	5.72(4.23,7.51)	-0.62(-0.95,-0.29)	47.97(35.45,62.91)	-0.51(-0.84,-0.18)	26.91(20.81,34.30)	-2.35(-2.63,-2.08)	0.60(0.46,0.75)	-2.51(-2.78,-2.25)
Iceland	4.00(2.89,5.38)	-0.23(-0.46,-0.00)	34.06(24.57,45.74)	-0.20(-0.43,0.03)	11.88(8.68,15.74)	-1.14(-1.41,-0.87)	0.25(0.18,0.32)	-1.39(-1.67,-1.10)
India	0.70(0.54,0.94)	0.97(0.68,1.26)	5.61(4.35,7.50)	1.13(0.84,1.42)	7.76(5.99,10.49)	-0.61(-0.82,-0.40)	0.18(0.14,0.25)	-0.66(-0.87,-0.44)
Indonesia	3.24(1.66,4.84)	1.00(0.94,1.06)	26.40(13.57,39.40)	1.07(1.00,1.13)	32.57(16.69,48.77)	0.07(-0.08,0.23)	0.77(0.40,1.17)	0.06(-0.09,0.21)
Iran (Islamic Republic of)	1.78(0.69,2.53)	2.05(1.60,2.50)	15.03(5.87,21.41)	2.11(1.66,2.56)	8.41(3.46,11.58)	0.23(-0.20,0.65)	0.18(0.08,0.25)	0.04(-0.38,0.47)
Iraq	2.37(1.27,4.01)	2.56(2.31,2.80)	19.87(10.61,33.54)	2.64(2.39,2.89)	13.53(7.37,22.21)	0.49(0.28,0.71)	0.31(0.17,0.51)	0.34(0.13,0.56)
Ireland	3.50(2.53,4.69)	1.38(1.11,1.65)	29.75(21.50,39.88)	1.43(1.16,1.70)	10.90(7.94,14.41)	-0.36(-0.54,-0.19)	0.23(0.17,0.30)	-0.65(-0.81,-0.48)
Israel	2.22(1.61,2.91)	0.92(0.48,1.36)	18.77(13.62,24.58)	0.97(0.52,1.42)	8.39(6.22,10.95)	-0.51(-0.83,-0.18)	0.18(0.14,0.23)	-0.70(-1.02,-0.38)
Italy	6.33(5.52,7.10)	4.66(3.57,5.77)	54.44(47.39,61.10)	4.71(3.61,5.83)	16.02(14.26,17.97)	3.30(2.38,4.23)	0.32(0.30,0.35)	3.06(2.16,3.96)
Jamaica	6.59(4.17,9.90)	2.15(1.53,2.77)	50.54(31.94,75.87)	2.13(1.50,2.76)	63.05(41.00,94.92)	1.96(1.36,2.55)	1.49(0.97,2.23)	1.97(1.40,2.54)
Japan	5.53(4.81,6.35)	3.79(3.51,4.07)	46.94(40.78,53.97)	3.85(3.56,4.13)	23.59(22.26,25.07)	2.04(1.86,2.21)	0.52(0.51,0.55)	1.86(1.69,2.03)
Jordan	2.37(1.29,4.20)	1.00(0.75,1.26)	20.06(10.95,35.49)	1.09(0.83,1.35)	11.31(6.16,20.16)	-1.30(-1.57,-1.03)	0.25(0.14,0.45)	-1.50(-1.77,-1.24)
Kazakhstan	4.39(3.59,5.28)	-0.17(-0.66,0.32)	36.17(29.55,43.52)	-0.06(-0.55,0.44)	30.93(26.38,35.80)	-2.00(-2.52,-1.48)	0.71(0.61,0.82)	-2.13(-2.66,-1.60)
Kenya	0.80(0.50,1.38)	1.77(1.69,1.85)	6.32(3.89,10.84)	1.93(1.82,2.03)	10.70(6.61,18.61)	1.31(1.16,1.47)	0.26(0.16,0.45)	1.31(1.15,1.47)
Kiribati	3.55(1.19,6.90)	0.87(0.81,0.94)	27.78(9.32,54.05)	0.92(0.85,0.99)	55.26(17.94,107.65)	0.54(0.48,0.61)	1.34(0.44,2.60)	0.54(0.47,0.60)
Kuwait	6.29(4.46,8.63)	3.28(2.26,4.31)	53.71(38.02,73.66)	3.35(2.33,4.38)	22.11(16.04,30.06)	1.62(0.62,2.63)	0.47(0.35,0.63)	1.40(0.42,2.39)
Kyrgyzstan	4.28(3.05,5.89)	-0.04(-0.44,0.36)	35.07(24.93,48.28)	0.07(-0.32,0.47)	35.03(25.20,46.80)	-1.49(-1.79,-1.18)	0.81(0.59,1.07)	-1.57(-1.87,-1.26)
Lao People's Democratic Republic	2.67(1.31,4.77)	0.51(0.45,0.58)	21.48(10.56,38.45)	0.68(0.61,0.74)	33.21(16.52,59.16)	-0.77(-0.89,-0.64)	0.79(0.40,1.41)	-0.85(-0.97,-0.73)
Latvia	8.71(6.18,11.97)	0.67(0.22,1.11)	73.35(52.15,100.94)	0.72(0.28,1.16)	34.16(24.76,45.88)	-0.50(-0.86,-0.14)	0.75(0.54,1.01)	-0.64(-1.00,-0.29)
Lebanon	2.47(1.32,4.19)	0.61(0.47,0.75)	20.92(11.21,35.58)	0.69(0.55,0.83)	10.30(5.65,17.43)	-1.67(-1.84,-1.50)	0.23(0.13,0.38)	-1.92(-2.08,-1.75)
Lesotho	1.26(0.59,2.31)	4.05(3.37,4.74)	9.18(4.29,16.82)	3.85(3.26,4.45)	20.85(9.76,37.70)	4.09(3.34,4.84)	0.52(0.24,0.94)	4.07(3.32,4.83)
Liberia	0.92(0.47,1.64)	1.89(1.69,2.10)	7.19(3.66,12.78)	2.08(1.87,2.28)	12.93(6.66,23.12)	0.77(0.55,0.99)	0.31(0.16,0.56)	0.69(0.47,0.91)
Libya	3.03(1.60,5.36)	2.46(2.19,2.73)	25.41(13.40,44.95)	2.50(2.23,2.77)	18.02(9.61,31.15)	1.01(0.90,1.12)	0.41(0.22,0.71)	0.89(0.79,0.98)
Lithuania	7.69(5.44,10.38)	0.71(0.30,1.12)	62.88(44.39,84.62)	0.77(0.36,1.19)	35.02(25.16,46.45)	-0.21(-0.63,0.22)	0.79(0.58,1.05)	-0.31(-0.74,0.12)

Table S6 The ASIR, ASPR, ASDR and ASMR of Uterine cancer in 204 countries and territories in 2021,and the EAPC from 1990 to 2021. (continued)

location_name	SAIR (1/100,000, 95%UI)	EAPC ASIR (EAPC, 95% CI)	SAPR (1/100,000, 95%UI)	EAPC SAPR (EAPC, 95% CI)	SADR (1/100,000, 95%UI)	EAPC SADR (EAPC, 95% CI)	SAMR (1/100,000, 95%UI)	EAPC SAMR (EAPC, 95% CI)
Luxembourg	4.04(3.27,4.97)	0.18(-0.19,0.55)	34.25(27.75,42.21)	0.23(-0.14,0.61)	13.49(11.30,16.27)	-1.36(-1.62,-1.09)	0.29(0.25,0.34)	-1.51(-1.78,-1.24)
Madagascar	1.08(0.58,1.94)	-0.19(-0.37,-0.01)	8.37(4.49,14.95)	-0.09(-0.27,0.09)	17.59(9.68,31.44)	-0.72(-0.88,-0.56)	0.42(0.23,0.76)	-0.74(-0.90,-0.58)
Malawi	0.74(0.36,1.32)	0.58(0.48,0.68)	5.69(2.80,10.15)	0.85(0.75,0.94)	11.49(5.77,20.92)	-0.21(-0.33,-0.10)	0.28(0.14,0.50)	-0.23(-0.35,-0.12)
Malaysia	2.86(1.69,4.53)	1.90(1.72,2.07)	23.76(14.03,37.70)	1.95(1.78,2.13)	19.37(11.73,30.69)	0.46(0.28,0.64)	0.46(0.28,0.72)	0.38(0.20,0.55)
Maldives	0.92(0.49,1.71)	-0.60(-0.68,-0.53)	7.76(4.11,14.34)	-0.45(-0.53,-0.36)	5.45(2.99,9.87)	-3.15(-3.29,-3.02)	0.12(0.07,0.23)	-3.38(-3.52,-3.23)
Mali	0.71(0.36,1.29)	0.41(0.33,0.49)	5.48(2.79,9.94)	0.54(0.46,0.62)	11.17(5.78,20.71)	-0.25(-0.34,-0.16)	0.27(0.14,0.50)	-0.28(-0.37,-0.19)
Malta	4.68(3.39,6.23)	1.49(1.17,1.81)	39.60(28.72,52.69)	1.52(1.20,1.84)	16.78(12.47,22.08)	0.32(0.04,0.59)	0.37(0.28,0.48)	0.12(-0.16,0.39)
Marshall Islands	6.00(2.15,13.67)	1.70(1.54,1.85)	47.45(16.96,108.25)	1.71(1.57,1.85)	77.48(28.55,179.45)	1.35(1.08,1.63)	1.88(0.70,4.32)	1.31(1.03,1.60)
Mauritania	0.95(0.48,1.66)	0.88(0.71,1.05)	7.56(3.82,13.20)	1.07(0.90,1.23)	11.13(5.55,19.40)	-0.46(-0.58,-0.34)	0.27(0.13,0.47)	-0.52(-0.64,-0.40)
Mauritius	8.39(6.78,10.12)	-0.13(-1.01,0.75)	69.85(56.48,84.21)	-0.11(-0.99,0.77)	56.65(47.26,65.46)	-0.79(-1.70,0.13)	1.31(1.09,1.51)	-0.84(-1.75,0.07)
Mexico	2.24(1.83,2.68)	3.20(2.60,3.79)	17.74(14.52,21.23)	3.34(2.76,3.93)	19.61(16.12,23.51)	1.97(1.29,2.65)	0.47(0.38,0.56)	1.92(1.25,2.60)
Micronesia (Federated States of)	5.34(2.45,10.46)	1.48(1.42,1.53)	42.54(19.58,83.26)	1.54(1.48,1.60)	62.42(28.39,124.04)	0.69(0.61,0.76)	1.51(0.69,3.00)	0.65(0.57,0.73)
Monaco	2.68(1.38,4.66)	1.35(1.10,1.60)	22.72(11.67,39.51)	1.37(1.11,1.62)	9.01(4.80,15.47)	0.69(0.50,0.88)	0.19(0.10,0.32)	0.55(0.37,0.73)
Mongolia	2.61(1.54,4.08)	0.92(0.72,1.12)	21.05(12.44,32.96)	1.15(0.95,1.34)	25.93(15.62,40.14)	-1.14(-1.34,-0.93)	0.61(0.37,0.95)	-1.21(-1.43,-1.00)
Montenegro	4.80(3.24,7.00)	0.69(0.43,0.95)	40.22(27.16,58.67)	0.74(0.49,1.01)	22.06(15.10,31.43)	-0.53(-0.87,-0.18)	0.49(0.34,0.70)	-0.64(-1.00,-0.28)
Morocco	0.77(0.39,1.39)	2.43(2.38,2.47)	6.39(3.24,11.55)	2.52(2.47,2.56)	5.59(2.91,10.05)	0.80(0.70,0.90)	0.13(0.07,0.23)	0.75(0.66,0.85)
Mozambique	1.24(0.54,2.47)	0.80(0.65,0.94)	9.30(4.08,18.54)	0.78(0.61,0.96)	21.48(9.53,43.93)	0.36(0.21,0.51)	0.52(0.23,1.05)	0.35(0.20,0.51)
Myanmar	2.98(1.50,5.27)	-0.14(-0.27,-0.00)	24.20(12.25,42.82)	0.01(-0.12,0.14)	32.57(16.93,56.87)	-1.54(-1.76,-1.33)	0.77(0.41,1.33)	-1.56(-1.78,-1.35)
Namibia	1.18(0.57,2.16)	0.93(0.66,1.20)	9.22(4.50,17.00)	1.06(0.85,1.26)	14.34(7.06,25.58)	-0.11(-0.44,0.22)	0.35(0.17,0.62)	-0.15(-0.49,0.19)
Nauru	6.37(2.35,12.69)	0.98(0.83,1.14)	50.85(18.74,101.32)	1.01(0.83,1.20)	72.72(27.14,146.57)	0.62(0.49,0.75)	1.75(0.66,3.52)	0.59(0.45,0.73)
Nepal	0.61(0.27,1.39)	0.97(0.62,1.33)	4.86(2.14,11.11)	1.18(0.84,1.52)	7.47(3.34,16.77)	-0.49(-0.85,-0.13)	0.18(0.08,0.40)	-0.54(-0.91,-0.17)
Netherlands	2.38(1.76,3.16)	-0.15(-0.30,0.00)	19.39(14.28,25.67)	-0.10(-0.26,0.05)	10.66(8.01,13.83)	-0.94(-1.11,-0.77)	0.24(0.18,0.31)	-1.04(-1.23,-0.86)
New Zealand	3.69(2.90,4.63)	1.70(1.43,1.97)	30.77(24.19,38.71)	1.77(1.50,2.04)	19.19(15.45,23.44)	0.37(0.14,0.60)	0.44(0.36,0.54)	0.25(0.02,0.49)
Nicaragua	1.04(0.59,1.70)	1.14(0.92,1.36)	8.30(4.69,13.55)	1.29(1.07,1.50)	9.45(5.53,15.40)	-0.26(-0.46,-0.06)	0.22(0.13,0.36)	-0.33(-0.53,-0.14)
Niger	0.48(0.25,0.85)	-0.22(-0.35,-0.10)	3.64(1.89,6.53)	-0.11(-0.23,0.00)	8.09(4.20,14.49)	-0.91(-1.08,-0.74)	0.20(0.10,0.35)	-0.94(-1.11,-0.77)
Nigeria	0.39(0.23,0.67)	1.02(0.85,1.18)	3.02(1.74,5.17)	1.15(0.97,1.33)	5.46(3.26,9.22)	0.01(-0.10,0.13)	0.13(0.08,0.22)	-0.02(-0.14,0.10)
Niue	6.26(2.70,12.77)	1.25(1.10,1.40)	51.54(22.21,105.00)	1.29(1.13,1.45)	53.63(23.91,107.68)	0.56(0.39,0.72)	1.23(0.55,2.45)	0.47(0.33,0.60)

Table S6 The ASIR, ASPR, ASDR and ASMR of Uterine cancer in 204 countries and territories in 2021,and the EAPC from 1990 to 2021. (continued)

location_name	SAIR (1/100,000, 95%UI)	EAPC ASIR (EAPC, 95% CI)	SAPR (1/100,000, 95%UI)	EAPC SAPR (EAPC, 95% CI)	SADR (1/100,000, 95%UI)	EAPC SADR (EAPC, 95% CI)	SAMR (1/100,000, 95%UI)	EAPC SAMR (EAPC, 95% CI)
North Macedonia	5.23(3.32,7.83)	1.22(0.76,1.68)	43.51(27.70,65.18)	1.35(0.87,1.82)	29.51(18.85,43.46)	-0.82(-1.11,-0.53)	0.68(0.44,0.99)	-0.95(-1.23,-0.66)
Northern Mariana Islands	10.75(6.37,17.37)	1.37(1.13,1.62)	89.16(52.75,144.04)	1.39(1.15,1.63)	73.08(44.35,112.95)	1.34(1.04,1.63)	1.71(1.03,2.63)	1.33(1.01,1.64)
Norway	3.05(2.52,3.67)	-0.69(-1.24,-0.14)	26.03(21.56,31.29)	-0.63(-1.19,-0.07)	9.65(8.53,11.02)	-2.30(-2.70,-1.91)	0.21(0.19,0.23)	-2.55(-2.92,-2.18)
Oman	0.50(0.24,0.96)	1.20(0.85,1.55)	4.27(2.05,8.10)	1.29(0.94,1.63)	2.27(1.11,4.32)	-0.77(-1.08,-0.46)	0.05(0.02,0.09)	-1.01(-1.34,-0.69)
Pakistan	2.38(1.42,3.74)	1.48(1.32,1.64)	18.27(10.90,28.74)	1.58(1.43,1.73)	34.55(20.56,54.94)	0.73(0.47,0.98)	0.82(0.49,1.29)	0.67(0.41,0.92)
Palau	0.74(0.38,1.29)	0.44(0.37,0.51)	6.11(3.10,10.57)	0.48(0.41,0.55)	6.45(3.21,11.09)	0.11(0.02,0.20)	0.15(0.07,0.25)	-0.03(-0.12,0.06)
Palestine	4.57(2.54,7.61)	1.05(0.85,1.26)	38.46(21.34,64.18)	1.11(0.90,1.33)	26.45(14.85,44.07)	-0.53(-0.69,-0.37)	0.60(0.34,0.98)	-0.68(-0.83,-0.53)
Panama	3.55(2.41,4.95)	2.38(1.96,2.79)	28.63(19.47,39.88)	2.50(2.08,2.91)	26.15(18.33,35.71)	1.26(0.86,1.65)	0.61(0.42,0.83)	1.20(0.80,1.60)
Papua New Guinea	2.62(1.11,5.38)	0.38(0.26,0.50)	20.81(8.76,42.75)	0.42(0.29,0.55)	35.19(14.85,73.44)	0.38(0.32,0.45)	0.85(0.36,1.78)	0.37(0.31,0.43)
Paraguay	2.41(1.34,4.00)	0.32(0.19,0.45)	19.31(10.75,32.10)	0.38(0.24,0.51)	22.02(12.20,36.60)	-0.58(-0.68,-0.48)	0.53(0.29,0.88)	-0.65(-0.75,-0.54)
Peru	3.45(1.93,5.68)	0.58(0.31,0.86)	28.01(15.68,46.20)	0.78(0.51,1.06)	25.56(14.24,40.97)	-1.69(-1.96,-1.41)	0.60(0.34,0.95)	-1.80(-2.08,-1.53)
Philippines	3.44(2.21,4.59)	0.41(0.28,0.53)	27.73(17.78,37.07)	0.42(0.29,0.55)	34.49(22.10,45.29)	0.13(0.03,0.24)	0.81(0.52,1.07)	0.17(0.06,0.28)
Poland	4.70(3.87,5.62)	0.66(0.44,0.87)	39.26(32.31,46.94)	0.78(0.56,0.99)	22.38(19.09,25.70)	-1.48(-1.74,-1.22)	0.51(0.43,0.58)	-1.71(-1.96,-1.45)
Portugal	4.31(3.16,5.82)	0.31(0.12,0.50)	36.71(26.85,49.51)	0.38(0.18,0.57)	14.12(10.59,18.67)	-1.75(-1.95,-1.54)	0.30(0.23,0.39)	-2.05(-2.27,-1.83)
Puerto Rico	6.02(4.21,8.35)	1.88(1.52,2.24)	48.13(33.59,66.74)	1.94(1.58,2.30)	35.96(25.92,48.42)	0.72(0.38,1.06)	0.82(0.59,1.10)	0.69(0.36,1.03)
Qatar	3.29(1.72,6.44)	1.54(1.07,2.02)	28.01(14.67,54.82)	1.62(1.15,2.09)	11.84(6.15,23.54)	-1.00(-1.39,-0.60)	0.25(0.13,0.49)	-1.31(-1.69,-0.93)
Republic of Korea	2.47(1.39,3.80)	1.21(0.74,1.69)	20.89(11.74,32.15)	1.37(0.92,1.82)	11.01(6.26,16.61)	-2.38(-3.05,-1.70)	0.24(0.14,0.36)	-2.72(-3.40,-2.03)
Republic of Moldova	4.67(3.76,5.81)	-0.19(-0.48,0.11)	38.82(31.23,48.32)	-0.11(-0.41,0.19)	22.60(18.83,26.80)	-1.57(-1.84,-1.29)	0.51(0.43,0.61)	-1.68(-1.96,-1.41)
Romania	4.62(3.28,6.26)	0.07(-0.15,0.28)	38.45(27.29,52.05)	0.20(-0.01,0.41)	26.05(19.19,34.68)	-2.04(-2.28,-1.81)	0.59(0.44,0.79)	-2.19(-2.42,-1.96)
Russian Federation	11.09(9.58,12.57)	0.19(-0.21,0.58)	93.12(80.50,105.58)	0.23(-0.17,0.63)	45.84(39.66,52.17)	-1.47(-1.78,-1.16)	1.00(0.87,1.14)	-1.66(-1.97,-1.34)
Rwanda	1.06(0.54,1.92)	-1.60(-2.06,-1.14)	8.28(4.22,14.96)	-1.20(-1.63,-0.76)	15.76(8.10,27.93)	-2.98(-3.43,-2.54)	0.38(0.20,0.67)	-3.03(-3.47,-2.58)
Saint Kitts and Nevis	4.21(2.97,5.69)	-1.26(-1.58,-0.95)	32.04(22.55,43.25)	-1.14(-1.45,-0.83)	43.63(31.06,57.61)	-2.38(-2.74,-2.02)	1.05(0.75,1.38)	-2.32(-2.66,-1.98)
Saint Lucia	5.30(3.97,6.86)	0.67(0.39,0.96)	40.43(30.24,52.47)	0.71(0.42,1.00)	53.91(41.62,67.97)	0.04(-0.17,0.25)	1.27(0.98,1.61)	0.03(-0.18,0.24)
Saint Vincent and the Grenadines	5.68(3.57,8.11)	0.69(0.49,0.90)	42.44(26.61,60.76)	0.66(0.45,0.87)	64.88(41.42,91.40)	0.41(0.22,0.60)	1.54(0.98,2.17)	0.45(0.25,0.64)
Samoa	4.46(2.14,8.25)	1.50(1.38,1.62)	36.27(17.43,67.11)	1.53(1.40,1.65)	41.69(20.64,74.29)	0.96(0.84,1.09)	1.00(0.50,1.78)	0.92(0.79,1.05)
San Marino	0.86(0.39,1.57)	0.78(0.33,1.24)	7.34(3.30,13.32)	0.80(0.34,1.26)	2.83(1.29,5.19)	0.28(-0.12,0.68)	0.06(0.03,0.11)	0.19(-0.20,0.58)

Table S6 The ASIR, ASPR, ASDR and ASMR of Uterine cancer in 204 countries and territories in 2021,and the EAPC from 1990 to 2021. (continued)

location_name	SAIR (1/100,000, 95%UI)	EAPC ASIR (EAPC, 95% CI)	SAPR (1/100,000, 95%UI)	EAPC SAPR (EAPC, 95% CI)	SADR (1/100,000, 95%UI)	EAPC SADR (EAPC, 95% CI)	SAMR (1/100,000, 95%UI)	EAPC SAMR (EAPC, 95% CI)
Sao Tome and Principe	1.24(0.59,2.37)	1.71(1.45,1.97)	9.96(4.71,18.93)	1.84(1.60,2.09)	14.43(6.99,26.98)	0.55(0.22,0.88)	0.35(0.17,0.64)	0.51(0.20,0.83)
Saudi Arabia	2.45(1.32,4.22)	3.85(3.67,4.02)	20.56(11.06,35.38)	3.92(3.75,4.09)	11.92(6.42,20.26)	1.13(1.00,1.26)	0.27(0.14,0.46)	0.91(0.78,1.05)
Senegal	0.89(0.45,1.60)	0.72(0.56,0.87)	6.99(3.55,12.60)	0.81(0.66,0.96)	12.59(6.39,22.49)	0.06(-0.09,0.21)	0.30(0.16,0.54)	0.04(-0.11,0.20)
Serbia	3.93(2.23,6.32)	0.53(0.28,0.78)	32.42(18.38,52.04)	0.62(0.38,0.87)	22.00(12.71,34.94)	-1.22(-1.47,-0.97)	0.51(0.30,0.81)	-1.31(-1.57,-1.05)
Seychelles	4.26(2.51,6.72)	0.94(0.68,1.19)	35.37(20.86,55.84)	0.97(0.71,1.22)	31.69(19.06,49.96)	-0.03(-0.29,0.23)	0.72(0.43,1.13)	-0.08(-0.35,0.18)
Sierra Leone	0.66(0.36,1.12)	1.74(1.64,1.85)	5.13(2.77,8.67)	1.82(1.71,1.93)	10.20(5.53,17.12)	1.19(1.05,1.32)	0.25(0.13,0.41)	1.15(1.01,1.28)
Singapore	2.23(1.63,2.94)	0.86(0.63,1.10)	18.87(13.73,24.86)	0.96(0.72,1.20)	10.37(7.74,13.48)	-1.77(-1.88,-1.65)	0.23(0.18,0.30)	-1.95(-2.06,-1.84)
Slovakia	5.48(3.07,8.85)	0.48(0.34,0.61)	45.11(25.23,72.73)	0.59(0.46,0.73)	31.40(18.17,49.47)	-1.27(-1.39,-1.14)	0.72(0.42,1.13)	-1.42(-1.54,-1.30)
Slovenia	5.49(3.78,7.50)	0.44(0.20,0.68)	46.72(32.14,63.89)	0.51(0.28,0.75)	19.09(13.66,26.21)	-1.64(-1.97,-1.31)	0.41(0.30,0.55)	-1.94(-2.28,-1.61)
Solomon Islands	4.07(1.91,7.56)	1.88(1.75,2.01)	32.31(15.16,60.20)	1.92(1.78,2.06)	54.61(25.93,101.23)	1.55(1.47,1.63)	1.34(0.64,2.47)	1.51(1.44,1.59)
Somalia	0.87(0.41,1.64)	-0.57(-0.65,-0.49)	6.47(3.01,12.23)	-0.49(-0.57,-0.41)	17.64(8.02,33.31)	-0.76(-0.83,-0.69)	0.43(0.20,0.82)	-0.77(-0.85,-0.70)
South Africa	1.28(0.98,1.91)	1.20(0.62,1.78)	9.84(7.53,14.77)	1.14(0.55,1.74)	13.93(10.70,20.96)	0.36(-0.13,0.86)	0.34(0.26,0.50)	0.45(-0.05,0.94)
South Sudan	1.13(0.57,2.05)	0.42(0.06,0.79)	8.67(4.31,15.70)	0.48(0.10,0.86)	18.25(9.01,33.15)	-0.11(-0.47,0.25)	0.44(0.22,0.80)	-0.15(-0.50,0.21)
Spain	4.12(3.01,5.47)	0.24(-0.02,0.51)	35.20(25.66,46.74)	0.29(0.02,0.56)	12.29(9.14,16.16)	-1.31(-1.52,-1.09)	0.26(0.19,0.33)	-1.54(-1.77,-1.31)
Sri Lanka	2.06(0.96,3.72)	0.28(0.07,0.48)	17.26(8.03,31.23)	0.35(0.14,0.55)	11.99(5.59,21.56)	-1.61(-1.83,-1.40)	0.27(0.13,0.48)	-1.70(-1.92,-1.49)
Sudan	1.40(0.62,2.54)	2.34(2.16,2.53)	11.53(5.11,20.95)	2.46(2.27,2.65)	11.98(5.45,21.09)	0.78(0.64,0.93)	0.28(0.13,0.49)	0.66(0.51,0.80)
Suriname	2.60(1.49,4.29)	1.08(0.85,1.30)	19.23(10.97,31.88)	1.09(0.87,1.32)	34.24(19.66,57.35)	0.73(0.54,0.93)	0.81(0.47,1.35)	0.72(0.52,0.92)
Sweden	2.36(1.76,3.10)	0.07(-0.40,0.53)	20.26(15.06,26.61)	0.09(-0.37,0.56)	6.78(5.03,8.90)	-0.82(-1.22,-0.41)	0.14(0.11,0.18)	-1.04(-1.42,-0.66)
Switzerland	3.54(2.63,4.72)	-0.03(-0.25,0.18)	30.16(22.35,40.15)	-0.00(-0.22,0.22)	10.80(8.10,14.16)	-1.00(-1.23,-0.78)	0.23(0.17,0.29)	-1.12(-1.36,-0.88)
Syrian Arab Republic	2.23(1.33,3.56)	0.84(0.63,1.05)	18.74(11.21,29.99)	0.92(0.71,1.12)	11.96(7.21,18.67)	-1.26(-1.61,-0.91)	0.27(0.16,0.42)	-1.43(-1.78,-1.07)
Taiwan (Province of China)	5.41(3.93,7.20)	3.00(2.50,3.51)	45.95(33.40,61.28)	3.04(2.54,3.55)	19.33(14.54,25.63)	1.46(1.03,1.90)	0.41(0.32,0.54)	1.35(0.92,1.77)
Tajikistan	2.83(1.32,5.76)	-0.92(-1.32,-0.52)	22.80(10.68,46.45)	-0.81(-1.21,-0.41)	31.82(14.90,65.29)	-1.55(-1.88,-1.22)	0.75(0.35,1.51)	-1.56(-1.88,-1.24)
Thailand	3.50(1.87,5.93)	1.69(1.39,1.98)	29.30(15.65,49.67)	1.76(1.46,2.06)	19.54(10.58,32.09)	0.03(-0.35,0.42)	0.44(0.24,0.71)	-0.15(-0.51,0.21)
Timor-Leste	1.98(0.97,3.58)	1.33(0.85,1.81)	15.95(7.84,28.92)	1.44(0.95,1.94)	23.64(11.87,43.04)	0.29(-0.16,0.74)	0.57(0.28,1.04)	0.23(-0.20,0.67)
Togo	0.83(0.43,1.45)	0.74(0.53,0.95)	6.48(3.33,11.29)	0.82(0.58,1.06)	12.00(6.33,20.31)	0.13(0.00,0.26)	0.29(0.15,0.49)	0.11(-0.02,0.24)
Tokelau	6.28(3.03,11.19)	1.36(1.18,1.54)	51.35(24.73,91.49)	1.44(1.25,1.62)	56.85(27.67,100.70)	0.32(0.15,0.48)	1.31(0.64,2.32)	0.20(0.06,0.34)
Tonga	4.01(1.93,7.16)	0.61(0.49,0.72)	32.78(15.78,58.66)	0.63(0.51,0.76)	35.58(17.59,64.46)	0.24(0.16,0.32)	0.85(0.42,1.53)	0.21(0.12,0.29)

Table S6 The ASIR, ASPR, ASDR and ASMR of Uterine cancer in 204 countries and territories in 2021,and the EAPC from 1990 to 2021. (continued)

location_name	SAIR (1/100,000, 95%UI)	EAPC ASIR (EAPC, 95% CI)	SAPR (1/100,000, 95%UI)	EAPC SAPR (EAPC, 95% CI)	SADR (1/100,000, 95%UI)	EAPC SADR (EAPC, 95% CI)	SAMR (1/100,000, 95%UI)	EAPC SAMR (EAPC, 95% CI)
Trinidad and Tobago	7.10(4.79,9.91)	1.29(0.93,1.65)	53.76(36.27,75.12)	1.34(0.99,1.70)	72.43(49.32,100.09)	0.51(0.12,0.91)	1.71(1.16,2.36)	0.48(0.09,0.87)
Tunisia	1.54(0.78,2.68)	1.63(1.55,1.72)	12.98(6.60,22.60)	1.69(1.60,1.78)	7.43(3.75,12.66)	-0.16(-0.24,-0.09)	0.17(0.08,0.28)	-0.34(-0.41,-0.26)
Turkmenistan	2.35(1.69,3.24)	0.19(-0.42,0.80)	19.04(13.68,26.28)	0.29(-0.32,0.90)	22.05(16.36,29.80)	-0.96(-1.52,-0.39)	0.51(0.38,0.69)	-1.03(-1.59,-0.46)
Tuvalu	4.94(2.35,9.10)	1.24(1.15,1.32)	39.84(18.99,73.50)	1.33(1.24,1.41)	54.40(25.42,99.55)	0.36(0.29,0.43)	1.31(0.62,2.41)	0.31(0.24,0.38)
Turkey	3.64(2.09,5.83)	1.14(0.83,1.45)	30.85(17.66,49.40)	1.25(0.95,1.55)	17.21(9.85,27.37)	-1.67(-2.06,-1.29)	0.38(0.22,0.61)	-1.91(-2.29,-1.54)
Uganda	1.66(0.85,2.89)	1.03(0.69,1.37)	12.91(6.62,22.52)	1.31(1.01,1.61)	24.41(12.49,42.52)	0.25(-0.17,0.67)	0.58(0.30,1.01)	0.21(-0.21,0.63)
Ukraine	6.30(3.34,10.27)	0.91(-0.31,2.14)	51.14(27.12,83.36)	0.96(-0.25,2.20)	43.07(23.84,69.94)	0.07(-1.19,1.35)	1.01(0.56,1.64)	0.01(-1.26,1.29)
United Arab Emirates	4.33(2.38,7.61)	0.40(0.16,0.65)	36.40(19.98,63.93)	0.47(0.23,0.71)	25.73(14.60,44.88)	-1.11(-1.37,-0.85)	0.57(0.32,0.99)	-1.36(-1.65,-1.07)
United Kingdom	3.67(3.45,3.89)	3.16(2.78,3.54)	31.08(29.28,32.95)	3.19(2.81,3.57)	14.03(13.16,14.94)	2.09(1.73,2.45)	0.31(0.30,0.32)	1.92(1.57,2.28)
United Republic of Tanzania	1.15(0.62,2.01)	0.14(-0.01,0.29)	9.01(4.81,15.74)	0.30(0.11,0.49)	16.70(9.09,29.45)	-0.49(-0.60,-0.37)	0.40(0.22,0.70)	-0.50(-0.61,-0.39)
United States of America	8.21(7.75,8.71)	2.47(2.35,2.59)	69.99(66.04,74.24)	2.49(2.37,2.60)	24.98(23.24,27.04)	1.87(1.72,2.02)	0.52(0.50,0.55)	1.74(1.58,1.89)
United States Virgin Islands	3.93(1.91,8.01)	0.13(-0.15,0.41)	30.51(14.87,62.41)	0.13(-0.15,0.41)	35.58(17.38,74.85)	-0.14(-0.39,0.10)	0.81(0.40,1.68)	-0.16(-0.43,0.11)
Uruguay	2.59(1.91,3.40)	0.01(-0.17,0.18)	21.42(15.76,28.17)	0.07(-0.11,0.24)	17.23(13.16,22.17)	-1.09(-1.29,-0.90)	0.39(0.30,0.50)	-1.18(-1.38,-0.98)
Uzbekistan	3.02(2.10,4.16)	1.16(0.82,1.51)	24.55(17.08,33.79)	1.24(0.90,1.58)	28.13(19.86,37.44)	0.09(-0.22,0.41)	0.66(0.46,0.87)	0.06(-0.27,0.39)
Vanuatu	3.37(1.62,5.94)	0.96(0.79,1.12)	26.76(12.84,46.95)	0.97(0.79,1.14)	44.64(20.87,79.11)	0.88(0.74,1.01)	1.09(0.51,1.92)	0.86(0.73,1.00)
Venezuela (Bolivarian Republic of)	3.02(1.97,4.32)	0.92(0.36,1.48)	24.10(15.64,34.34)	1.05(0.50,1.61)	27.05(17.75,37.97)	-0.23(-0.82,0.35)	0.64(0.42,0.90)	-0.35(-0.94,0.25)
Viet Nam	1.27(0.69,2.18)	1.86(1.71,2.01)	10.62(5.77,18.24)	1.94(1.79,2.09)	8.15(4.43,13.49)	0.11(-0.01,0.22)	0.19(0.10,0.31)	0.04(-0.08,0.15)
Yemen	0.97(0.49,1.74)	2.07(1.89,2.24)	7.91(4.05,14.29)	2.17(1.99,2.35)	9.40(4.89,16.36)	0.78(0.64,0.93)	0.23(0.12,0.39)	0.70(0.56,0.83)
Zambia	1.80(0.65,4.65)	0.82(0.38,1.26)	13.88(5.00,35.96)	1.08(0.58,1.59)	27.01(9.96,70.97)	-0.12(-0.44,0.19)	0.65(0.24,1.69)	-0.14(-0.45,0.17)
Zimbabwe	2.69(1.27,4.88)	3.52(2.44,4.61)	20.41(9.56,37.04)	3.57(2.60,4.55)	43.33(20.17,79.62)	4.13(3.00,5.28)	1.05(0.49,1.93)	4.13(3.00,5.27)

DALYs disability-adjusted life-years, ASIR age-standardized incidence rate, ASPR age-standardized prevalence rate, ASMR age-standardized mortality rate, ASDR age-standardized DALYs rate, EAPC estimated annual percentage change, CI confdence interval, UI uncertainty intervals

Table S7 The ASIR, ASPR, ASDR and ASMR of of Diabetes mellitus type 2 in 204 countries and territories in 2021,and the EAPC from 1990 to 2021. (continued)

Diabetes mellitus type location	SAIR (1/100,000, 95%UI)	EAPC ASIR (EAPC, 95% CI)	SAPR (1/100,000, 95%UI)	EAPC SAPR (EAPC, 95% CI)	SADR (1/100,000, 95%UI)	EAPC SADR (EAPC, 95% CI)	SAMR (1/100,000, 95%UI)	EAPC SAMR (EAPC, 95% CI)
Afghanistan	839.47(583.54,1127.77)	3.45(3.40,3.50)	10137.61(8508.04,12044.69)	3.90(3.78,4.02)	982.33(680.73,1361.06)	3.05(2.97,3.13)	6.62(3.57,11.51)	1.70(1.63,1.77)
Albania	149.19(104.63,199.53)	1.78(1.71,1.84)	1341.47(1072.03,1664.74)	1.60(1.53,1.67)	111.20(70.38,165.07)	1.46(1.37,1.55)	0.16(0.09,0.25)	-0.12(-0.41,0.16)
Algeria	498.85(365.19,643.16)	3.32(3.29,3.35)	5541.21(4667.60,6484.22)	3.25(3.16,3.33)	451.19(307.82,622.49)	2.85(2.80,2.90)	1.64(1.05,2.47)	1.48(1.30,1.67)
American Samoa	1287.30(894.29,1734.54)	2.94(2.77,3.12)	17113.67 (14294.62,20406.84)	2.96(2.77,3.14)	1970.85(1446.62,2637.66)	2.53(2.24,2.82)	17.25(11.61,24.85)	2.02(1.59,2.46)
Andorra	186.88(127.70,257.08)	2.83(2.80,2.86)	2787.66(2242.84,3379.87)	2.66(2.63,2.68)	180.41(113.26,262.67)	2.32(2.28,2.35)	0.19(0.10,0.31)	-1.03(-1.22,-0.84)
Angola	219.11(151.72,296.75)	2.33(2.24,2.42)	3394.13(2843.80,4058.55)	2.53(2.44,2.63)	431.86(309.96,589.34)	0.98(0.87,1.10)	3.62(2.23,5.55)	-0.48(-0.64,-0.32)
Antigua and Barbuda	499.20(356.09,662.94)	1.70(1.68,1.71)	5517.23(4582.05,6603.87)	1.78(1.74,1.82)	608.38(439.32,839.81)	0.39(0.27,0.50)	3.10(2.63,3.60)	-1.79(-2.03,-1.56)
Argentina	228.55(164.79,296.27)	2.30(2.23,2.36)	2218.71(1768.06,2680.14)	3.27(3.22,3.32)	197.82(142.64,270.43)	1.19(1.07,1.31)	1.09(0.93,1.27)	-1.34(-1.52,-1.15)
Armenia	236.93(162.10,323.51)	1.83(1.64,2.02)	2503.16(2047.31,3051.54)	1.60(1.39,1.81)	239.35(171.41,332.40)	1.18(0.92,1.44)	1.15(0.95,1.36)	0.10(-0.54,0.75)
Australia	120.60(79.14,171.05)	1.89(1.81,1.98)	1146.03(861.17,1478.77)	2.07(2.02,2.12)	96.46(64.30,138.43)	1.29(1.20,1.37)	0.40(0.33,0.47)	-0.84(-1.22,-0.45)
Austria	117.26(79.18,161.02)	2.88(2.80,2.96)	1594.83(1247.66,1968.15)	2.98(2.89,3.06)	116.41(74.37,174.05)	1.94(1.89,2.00)	0.17(0.14,0.20)	-2.85(-3.05,-2.65)
Azerbaijan	288.02(201.10,382.65)	3.11(2.94,3.28)	3351.88(2769.29,4009.03)	3.12(2.99,3.26)	291.39(199.49,418.61)	2.01(1.80,2.21)	1.05(0.71,1.43)	-0.73(-1.23,-0.24)
Bahamas	485.80(345.24,652.94)	1.73(1.67,1.78)	5912.95(4857.07,7121.51)	2.15(2.07,2.22)	692.79(493.66,947.28)	0.59(0.47,0.71)	4.18(3.03,5.59)	-1.49(-1.76,-1.22)
Bahrain	591.88(413.38,805.24)	2.54(2.45,2.64)	5588.45(4564.29,6712.30)	2.79(2.70,2.89)	604.72(440.09,812.03)	1.63(1.43,1.82)	4.33(3.11,5.76)	0.19(-0.17,0.55)
Bangladesh	370.79(261.84,493.33)	3.01(2.89,3.14)	5426.92(4612.73,6392.41)	2.87(2.77,2.98)	525.19(378.12,720.27)	1.99(1.82,2.16)	3.67(2.28,5.66)	0.93(0.59,1.27)
Barbados	484.55(349.39,643.52)	1.80(1.68,1.92)	5306.02(4384.17,6398.48)	2.08(2.01,2.16)	652.32(478.84,885.70)	0.40(0.28,0.53)	4.30(3.16,5.76)	-1.40(-1.63,-1.18)
Belarus	124.78(88.10,167.16)	1.58(1.45,1.72)	1381.14(1117.62,1662.91)	1.35(1.19,1.50)	123.81(84.25,175.76)	1.27(1.06,1.48)	0.47(0.34,0.63)	0.21(-1.08,1.51)
Belgium	201.84(139.04,276.91)	2.48(2.44,2.53)	3040.61(2460.82,3663.80)	2.58(2.53,2.63)	215.48(136.37,318.76)	2.18(2.04,2.31)	0.17(0.14,0.20)	-3.03(-3.34,-2.72)
Belize	499.17(367.50,662.23)	2.27(2.15,2.38)	5446.26(4549.69,6495.69)	2.37(2.23,2.50)	751.91(577.82,982.75)	1.00(0.74,1.26)	6.24(5.09,7.57)	-0.18(-0.63,0.28)
Benin	280.85(193.01,393.69)	2.67(2.48,2.87)	4546.91(3862.24,5369.26)	3.12(2.97,3.28)	463.26(326.64,639.20)	2.00(1.89,2.11)	2.96(1.82,4.55)	0.50(0.24,0.77)
Bermuda	262.70(187.26,351.08)	1.82(1.73,1.90)	3652.34(3040.45,4322.82)	2.24(2.17,2.31)	334.93(224.61,477.62)	0.95(0.80,1.09)	0.77(0.59,0.97)	-3.45(-3.71,-3.19)
Bhutan	255.79(181.32,342.16)	1.97(1.89,2.05)	3407.66(2852.98,4031.82)	1.88(1.77,2.00)	324.62(228.27,452.76)	0.79(0.70,0.87)	2.09(1.21,3.28)	-0.88(-1.03,-0.72)
Bolivia (Plurinational State of)	251.49(178.53,343.94)	2.04(1.97,2.11)	2546.82(2105.51,3072.80)	2.04(1.98,2.10)	355.04(253.31,483.67)	0.11(-0.00,0.23)	3.55(2.16,5.55)	-1.18(-1.34,-1.01)
Bosnia and Herzegovina	226.88(153.98,312.65)	2.41(2.33,2.49)	1790.35(1393.94,2240.17)	2.42(2.30,2.55)	185.53(123.45,269.04)	1.68(1.55,1.81)	0.89(0.56,1.33)	0.15(-0.11,0.41)
Botswana	173.63(121.09,237.16)	2.28(2.23,2.32)	2244.06(1868.69,2665.40)	2.58(2.53,2.62)	300.82(214.85,413.82)	1.54(1.09,1.98)	2.77(1.62,4.30)	0.68(-0.13,1.51)
Brazil	231.79(163.72,313.88)	0.77(0.67,0.88)	2347.60(1884.58,2858.15)	0.96(0.88,1.05)	302.12(235.58,385.28)	-0.55(-0.71,-0.39)	2.40(2.25,2.56)	-2.07(-2.29,-1.85)
Brunei Darussalam	561.67(382.54,768.61)	3.89(3.75,4.02)	6401.83(5254.19,7775.12)	4.29(4.10,4.48)	598.90(423.98,820.88)	1.35(0.97,1.74)	3.60(2.38,5.15)	-1.34(-1.75,-0.94)

Table S7 The ASIR, ASPR, ASDR and ASMR of of Diabetes mellitus type 2 in 204 countries and territories in 2021,and the EAPC from 1990 to 2021. (continued)

Diabetes mellitus type location	SAIR (1/100,000, 95%UI)	EAPC ASIR (EAPC, 95% CI)	SAPR (1/100,000, 95%UI)	EAPC SAPR (EAPC, 95% CI)	SADR (1/100,000, 95%UI)	EAPC SADR (EAPC, 95% CI)	SAMR (1/100,000, 95%UI)	EAPC SAMR (EAPC, 95% CI)
Bulgaria	217.13(160.44,285.83)	2.03(1.92,2.13)	1702.75(1362.78,2084.43)	1.79(1.70,1.88)	185.77(131.44,257.21)	1.14(1.04,1.23)	1.07(0.82,1.36)	-0.19(-0.47,0.08)
Burkina Faso	169.41(118.73,232.55)	2.72(2.62,2.82)	2693.03(2270.38,3198.23)	3.18(3.09,3.27)	339.58(244.98,460.79)	0.96(0.86,1.05)	3.04(1.84,4.54)	-0.57(-0.72,-0.43)
Burundi	106.41(72.76,146.88)	1.32(1.25,1.40)	1659.91(1358.18,1994.96)	1.62(1.55,1.69)	332.85(228.64,470.06)	-0.77(-0.97,-0.58)	4.01(2.25,6.44)	-1.79(-2.04,-1.55)
Cabo Verde	236.75(160.63,327.86)	2.77(2.54,3.00)	3990.89(3344.12,4755.94)	2.59(2.40,2.78)	382.02(270.36,524.71)	2.11(1.64,2.59)	2.15(1.37,3.22)	1.35(0.39,2.32)
Cambodia	192.34(136.97,259.40)	2.36(2.28,2.44)	2172.41(1821.75,2584.51)	2.55(2.48,2.62)	312.57(219.69,429.40)	0.55(0.38,0.71)	3.22(1.88,5.16)	-0.66(-0.86,-0.46)
Cameroon	192.53(133.80,262.26)	2.57(2.46,2.68)	3038.66(2553.37,3619.79)	2.93(2.80,3.06)	432.77(300.44,597.48)	1.35(1.08,1.62)	4.40(2.44,7.09)	0.43(0.06,0.80)
Canada	181.04(119.47,255.03)	3.85(3.71,3.99)	1306.61(931.11,1757.63)	6.94(6.77,7.11)	105.54(69.92,154.94)	2.45(2.19,2.71)	0.45(0.37,0.53)	-1.74(-2.08,-1.40)
Central African Republic	314.81(212.24,439.50)	2.56(2.47,2.65)	4939.03(4103.85,5860.73)	2.79(2.66,2.91)	652.30(468.44,896.96)	1.23(1.12,1.34)	5.86(3.30,9.43)	-0.11(-0.28,0.06)
Chad	204.80(140.39,282.78)	2.55(2.40,2.71)	3154.18(2625.40,3757.03)	2.70(2.55,2.84)	407.39(289.25,557.70)	2.09(1.83,2.36)	3.78(2.22,5.96)	1.48(1.09,1.87)
Chile	233.87(157.10,323.27)	2.33(2.19,2.47)	2165.26(1719.72,2659.89)	2.69(2.55,2.83)	166.64(113.00,235.85)	1.34(1.22,1.46)	0.52(0.44,0.61)	-1.98(-2.34,-1.63)
China	279.45(195.29,377.89)	1.43(1.30,1.57)	4888.34(4124.06,5747.30)	2.62(2.32,2.92)	300.92(198.17,429.55)	1.83(1.57,2.10)	0.46(0.34,0.62)	-2.28(-2.58,-1.97)
Colombia	280.41(198.78,371.62)	0.68(0.47,0.90)	3811.88(3203.75,4498.90)	0.99(0.80,1.17)	362.06(255.10,504.40)	-0.13(-0.42,0.16)	1.42(1.13,1.77)	-2.78(-3.27,-2.29)
Comoros	162.45(107.45,227.79)	2.45(2.31,2.59)	2651.20(2213.58,3174.16)	2.63(2.54,2.72)	411.54(291.55,558.98)	0.39(0.20,0.58)	3.99(2.28,6.34)	-1.10(-1.43,-0.77)
Congo	225.45(153.83,314.84)	2.31(2.20,2.42)	3269.89(2674.53,3914.64)	2.46(2.36,2.56)	575.07(400.85,802.58)	0.72(0.48,0.96)	6.76(3.99,10.88)	-0.18(-0.49,0.14)
Cook Islands	1119.85(804.94,1468.43)	2.08(1.98,2.17)	15011.64 (12649.82,17773.19)	2.17(2.01,2.32)	1565.11(1140.43,2120.06)	0.49(0.43,0.55)	11.84(7.90,17.11)	-1.31(-1.47,-1.16)
Costa Rica	353.00(247.33,474.88)	1.77(1.65,1.88)	4988.53(4171.44,5925.27)	2.08(2.01,2.15)	452.99(315.70,639.65)	1.40(1.27,1.52)	1.50(1.24,1.79)	-1.23(-1.99,-0.47)
Croatia	178.41(125.15,243.70)	1.71(1.66,1.76)	1431.62(1100.36,1798.31)	1.53(1.46,1.60)	136.65(89.24,200.50)	1.19(1.14,1.24)	0.49(0.39,0.60)	-0.28(-0.58,0.03)
Cuba	310.91(222.08,416.39)	1.36(1.27,1.45)	4334.47(3653.41,5104.85)	1.68(1.59,1.78)	395.06(262.83,567.63)	0.46(0.27,0.65)	0.78(0.61,0.96)	-4.26(-4.85,-3.67)
Cyprus	175.37(112.58,252.77)	1.90(1.88,1.93)	2395.98(1846.83,2986.00)	1.91(1.88,1.95)	169.54(110.63,245.05)	0.34(0.22,0.46)	0.43(0.27,0.64)	-3.97(-4.37,-3.57)
Czechia	189.94(134.02,261.36)	1.67(1.61,1.74)	1563.53(1221.89,1958.18)	1.55(1.50,1.60)	146.83(99.53,216.16)	1.36(1.28,1.44)	0.48(0.38,0.59)	0.21(-0.24,0.67)
Côte d'Ivoire	215.39(147.59,297.18)	2.50(2.42,2.57)	3444.03(2860.33,4067.80)	2.70(2.61,2.79)	413.13(294.27,563.06)	2.37(2.14,2.60)	3.46(1.96,5.44)	1.93(1.54,2.32)
Democratic People's Republic of Korea	262.00(184.10,352.76)	2.16(2.14,2.18)	3732.62(3102.18,4399.45)	2.13(2.10,2.16)	304.49(209.32,426.88)	1.64(1.62,1.66)	1.52(0.80,2.61)	0.44(0.37,0.51)
Democratic Republic of the Congo	189.44(132.13,256.15)	2.26(2.22,2.30)	2954.87(2446.58,3522.87)	2.37(2.32,2.41)	423.92(299.60,589.32)	0.98(0.90,1.06)	4.17(2.41,6.65)	-0.06(-0.17,0.05)
Denmark	153.84(105.86,209.01)	2.72(2.62,2.81)	2101.26(1672.63,2576.85)	2.73(2.61,2.85)	126.89(79.06,183.33)	1.83(1.68,1.99)	0.16(0.13,0.19)	-3.22(-3.72,-2.71)
Djibouti	102.54(70.33,140.59)	2.12(2.07,2.17)	1580.78(1303.12,1889.86)	2.43(2.39,2.48)	300.71(206.10,430.58)	0.95(0.79,1.11)	3.56(1.97,6.12)	0.19(-0.04,0.41)
Dominica	536.01(371.16,721.83)	1.71(1.66,1.76)	6807.06(5640.98,8107.20)	1.95(1.85,2.05)	840.44(611.19,1148.81)	1.10(1.05,1.15)	5.65(3.73,8.03)	-0.28(-0.48,-0.07)

Table S7 The ASIR, ASPR, ASDR and ASMR of of Diabetes mellitus type 2 in 204 countries and territories in 2021,and the EAPC from 1990 to 2021. (continued)

Diabetes mellitus type location	SAIR (1/100,000, 95%UI)	EAPC ASIR (EAPC, 95% CI)	SAPR (1/100,000, 95%UI)	EAPC SAPR (EAPC, 95% CI)	SADR (1/100,000, 95%UI)	EAPC SADR (EAPC, 95% CI)	SAMR (1/100,000, 95%UI)	EAPC SAMR (EAPC, 95% CI)
Dominican Republic	439.83(314.63,589.70)	2.61(2.55,2.67)	5451.55(4520.90,6497.79)	2.68(2.61,2.75)	634.55(454.80,861.96)	2.17(2.08,2.25)	3.74(2.48,5.47)	1.23(0.99,1.47)
Ecuador	311.35(221.27,408.67)	2.96(2.86,3.06)	3387.16(2866.87,3994.92)	2.86(2.74,2.98)	361.35(267.15,473.00)	1.10(0.84,1.35)	2.50(1.81,3.33)	-0.65(-1.22,-0.08)
Egypt	404.75(295.73,534.40)	4.66(4.57,4.76)	3926.95(3214.60,4692.85)	4.63(4.55,4.70)	440.66(325.41,593.78)	2.41(2.25,2.57)	3.38(2.20,4.90)	0.50(0.29,0.71)
El Salvador	343.14(248.21,462.23)	2.23(2.08,2.38)	4131.14(3460.85,4925.68)	2.10(1.98,2.21)	577.58(429.33,759.38)	1.77(1.63,1.90)	5.36(3.64,7.53)	1.43(1.21,1.65)
Equatorial Guinea	239.44(163.11,333.12)	2.60(2.58,2.63)	3591.20(2951.32,4321.92)	2.67(2.66,2.69)	487.18(330.82,685.38)	0.93(0.82,1.03)	4.46(2.29,7.57)	-0.48(-0.65,-0.30)
Eritrea	141.01(94.88,195.01)	2.19(2.16,2.23)	2188.02(1797.50,2626.29)	2.63(2.60,2.66)	452.38(313.68,625.59)	0.89(0.79,1.00)	5.70(3.33,8.93)	0.10(-0.04,0.24)
Estonia	192.21(135.46,259.31)	2.61(2.48,2.74)	2024.78(1602.11,2518.95)	2.57(2.43,2.70)	175.75(117.87,253.20)	2.58(2.49,2.67)	0.54(0.42,0.67)	1.85(0.26,3.46)
Eswatini	206.19(140.15,285.50)	2.09(1.81,2.38)	2555.64(2082.86,3056.52)	2.23(1.99,2.46)	507.94(324.10,758.23)	2.29(1.43,3.16)	6.71(3.31,11.80)	2.36(1.21,3.52)
Ethiopia	124.89(85.27,170.73)	0.54(0.41,0.66)	2055.18(1689.17,2445.39)	0.76(0.64,0.87)	320.23(246.50,404.31)	-2.46(-2.73,-2.20)	3.13(2.28,4.21)	-4.28(-4.61,-3.94)
Fiji	948.61(706.20,1266.39)	2.35(2.27,2.43)	10620.73 (9053.50,12409.61)	2.46(2.39,2.53)	2302.97(1727.28,3036.69)	1.36(1.17,1.54)	32.99(22.56,46.04)	0.95(0.68,1.23)
Finland	274.91(185.02,374.92)	2.61(2.51,2.72)	3616.66(2840.11,4462.84)	2.84(2.71,2.96)	229.79(143.11,341.32)	2.63(2.51,2.76)	0.11(0.09,0.14)	-1.38(-1.84,-0.91)
France	126.14(85.76,173.04)	2.86(2.75,2.96)	1754.50(1390.15,2178.71)	3.50(3.33,3.68)	107.91(67.92,160.19)	2.63(2.50,2.76)	0.16(0.13,0.19)	-1.97(-2.44,-1.50)
Gabon	236.57(159.86,330.16)	2.32(2.22,2.41)	3519.66(2915.50,4248.45)	2.47(2.38,2.55)	485.67(341.58,668.85)	0.91(0.66,1.17)	4.61(2.58,7.56)	-0.25(-0.64,0.15)
Gambia	201.89(138.91,276.50)	2.70(2.62,2.78)	3107.10(2603.51,3690.21)	2.80(2.72,2.88)	411.90(288.33,580.02)	2.03(1.86,2.21)	3.94(2.26,6.40)	1.33(1.02,1.64)
Georgia	278.21(194.74,377.09)	3.47(3.28,3.65)	3169.79(2610.88,3817.81)	3.20(3.02,3.37)	274.95(188.79,393.34)	2.88(2.68,3.08)	0.93(0.76,1.09)	1.57(0.95,2.20)
Germany	183.49(127.85,248.06)	3.35(3.23,3.47)	2506.62(2029.46,3043.11)	3.46(3.33,3.60)	158.34(102.40,227.76)	2.56(2.34,2.78)	0.25(0.21,0.30)	-2.11(-2.34,-1.88)
Ghana	202.29(139.16,276.59)	2.12(2.06,2.19)	3212.54(2645.08,3806.84)	2.36(2.25,2.47)	407.43(294.60,551.40)	0.76(0.63,0.89)	3.76(2.34,5.65)	-0.34(-0.60,-0.08)
Greece	210.72(142.28,298.19)	2.05(2.00,2.09)	3415.13(2785.65,4126.79)	2.55(2.52,2.58)	238.65(152.79,346.07)	2.11(2.05,2.17)	0.21(0.18,0.25)	-1.30(-1.51,-1.10)
Greenland	118.10(80.61,156.98)	4.61(4.46,4.76)	1255.74(965.89,1548.79)	7.84(7.70,7.98)	108.23(74.54,149.79)	0.75(0.46,1.04)	0.63(0.39,0.93)	-3.07(-3.44,-2.70)
Grenada	589.76(412.21,792.67)	1.68(1.64,1.73)	6663.37(5515.12,8073.68)	1.73(1.68,1.78)	903.87(682.91,1206.30)	-0.03(-0.22,0.16)	7.11(5.54,9.00)	-1.64(-1.97,-1.32)
Guam	453.83(331.07,590.35)	1.94(1.86,2.01)	6731.60(5740.68,7832.55)	2.02(1.96,2.08)	622.69(453.38,841.91)	1.16(0.97,1.35)	3.75(2.70,4.99)	-0.09(-0.61,0.44)
Guatemala	515.47(369.97,686.74)	3.53(3.35,3.71)	5896.16(4928.95,7075.38)	3.28(3.15,3.40)	1011.78(809.47,1260.77)	3.13(2.91,3.35)	11.69(9.48,14.33)	3.16(2.78,3.55)
Guinea	179.18(124.03,245.47)	2.22(1.97,2.48)	2762.01(2328.80,3281.64)	2.62(2.37,2.87)	413.51(292.98,560.18)	1.59(1.52,1.66)	4.39(2.62,6.77)	0.92(0.72,1.12)
Guinea-Bissau	253.66(171.64,355.33)	2.22(2.15,2.30)	3962.40(3307.19,4713.49)	2.39(2.30,2.48)	602.19(423.56,825.64)	1.32(1.11,1.53)	6.60(3.88,10.25)	0.69(0.41,0.97)
Guyana	944.54(662.16,1258.80)	1.96(1.87,2.05)	10765.53 (8919.23,12858.86)	2.53(2.41,2.64)	1421.92(1040.96,1907.20)	1.01(0.72,1.29)	10.92(7.27,15.41)	-0.55(-1.09,-0.01)

Table S7 The ASIR, ASPR, ASDR and ASMR of of Diabetes mellitus type 2 in 204 countries and territories in 2021,and the EAPC from 1990 to 2021. (continued)

Diabetes mellitus type location	SAIR (1/100,000, 95%UI)	EAPC ASIR (EAPC, 95% CI)	SAPR (1/100,000, 95%UI)	EAPC SAPR (EAPC, 95% CI)	SADR (1/100,000, 95%UI)	EAPC SADR (EAPC, 95% CI)	SAMR (1/100,000, 95%UI)	EAPC SAMR (EAPC, 95% CI)
Haiti	691.46(480.19,928.81)	1.58(1.54,1.61)	7783.40(6407.69,9292.70)	1.92(1.87,1.98)	1188.26(841.61,1643.64)	0.51(0.45,0.57)	10.99(6.35,17.63)	-0.62(-0.72,-0.52)
Honduras	412.95(293.02,547.04)	2.31(2.24,2.38)	5101.17(4273.46,6077.20)	2.16(2.10,2.21)	553.38(387.23,771.86)	1.37(1.26,1.48)	3.39(1.97,5.44)	0.09(-0.18,0.35)
Hungary	197.49(144.61,260.44)	1.66(1.51,1.81)	1610.98(1266.90,1990.58)	1.40(1.26,1.53)	158.09(107.62,223.37)	0.87(0.78,0.97)	0.64(0.52,0.78)	-0.91(-1.32,-0.49)
Iceland	195.46(135.77,262.94)	2.88(2.83,2.92)	3033.37(2475.76,3620.25)	2.81(2.77,2.85)	190.23(119.97,275.92)	2.65(2.61,2.69)	0.06(0.05,0.08)	-3.05(-3.39,-2.72)
India	280.24(198.92,374.24)	1.96(1.89,2.04)	3940.73(3276.52,4654.48)	2.12(2.09,2.16)	354.92(263.69,466.21)	1.33(1.20,1.47)	2.20(1.78,2.68)	0.14(-0.06,0.35)
Indonesia	207.74(149.23,276.89)	0.25(-0.49,1.00)	2103.87(1700.54,2541.99)	-0.47(-1.45,0.51)	299.75(235.33,381.04)	-0.25(-0.57,0.07)	3.01(2.20,4.18)	-0.17(-0.31,-0.04)
Iran (Islamic Republic of)	334.78(239.41,444.76)	2.81(2.69,2.93)	3362.69(2752.26,4040.17)	2.69(2.52,2.87)	292.87(210.77,398.32)	2.22(2.09,2.35)	1.09(0.93,1.34)	0.75(0.60,0.91)
Iraq	802.93(570.48,1077.34)	2.80(2.69,2.90)	9179.42(7670.72,10979.54)	2.81(2.64,2.97)	794.17(561.38,1109.88)	1.60(1.49,1.72)	3.83(2.31,6.01)	-0.67(-0.84,-0.50)
Ireland	164.47(112.90,224.22)	2.08(2.01,2.16)	2158.67(1705.32,2673.82)	1.77(1.70,1.84)	138.55(85.80,208.10)	1.50(1.43,1.57)	0.09(0.08,0.11)	-2.82(-3.15,-2.49)
Israel	174.84(114.49,249.20)	1.67(1.53,1.81)	2191.92(1725.53,2723.32)	1.27(1.22,1.31)	158.16(104.79,229.00)	0.43(0.36,0.50)	0.42(0.35,0.49)	-2.60(-2.97,-2.24)
Italy	162.60(92.00,248.58)	0.86(0.69,1.02)	2010.90(1390.06,2692.40)	1.48(1.25,1.71)	142.24(87.35,213.98)	0.77(0.56,0.98)	0.20(0.18,0.21)	-3.33(-3.49,-3.18)
Jamaica	373.27(266.33,490.05)	1.69(1.62,1.77)	4243.85(3510.60,5007.57)	2.44(2.36,2.51)	644.01(483.85,844.64)	0.62(0.39,0.84)	5.93(4.11,8.33)	-0.70(-1.05,-0.34)
Japan	192.76(131.96,267.87)	1.30(1.22,1.37)	2739.22(2240.65,3295.96)	2.19(2.14,2.25)	186.64(122.60,268.22)	1.76(1.68,1.84)	0.13(0.12,0.14)	-3.88(-4.56,-3.20)
Jordan	594.47(428.25,777.94)	2.44(2.31,2.57)	5698.86(4746.81,6739.63)	2.73(2.60,2.86)	485.24(338.69,667.11)	0.38(0.18,0.58)	2.13(1.43,2.99)	-3.48(-4.06,-2.90)
Kazakhstan	312.54(219.89,412.19)	2.67(2.58,2.76)	4222.22(3591.53,4913.22)	3.08(2.94,3.23)	330.17(220.07,469.14)	2.28(2.09,2.46)	0.64(0.53,0.77)	-1.31(-1.96,-0.66)
Kenya	77.83(53.79,106.27)	1.18(1.11,1.25)	1142.51(931.36,1363.11)	0.93(0.86,1.00)	229.33(174.60,291.95)	0.94(0.79,1.08)	2.78(1.95,3.87)	0.91(0.67,1.16)
Kiribati	936.37(694.44,1214.72)	2.22(2.08,2.36)	11618.32(9795.80,13629.47)	2.18(2.08,2.29)	2283.21(1647.25,3107.31)	1.40(1.30,1.50)	31.36(19.97,46.85)	1.05(0.95,1.15)
Kuwait	678.02(482.92,898.45)	2.50(2.43,2.57)	7303.64(6175.18,8575.48)	2.56(2.47,2.66)	530.08(347.25,756.34)	1.70(1.53,1.88)	0.77(0.61,0.94)	-2.87(-3.73,-2.00)
Kyrgyzstan	211.28(148.69,278.36)	3.02(2.87,3.16)	2537.40(2094.12,3025.51)	2.69(2.56,2.83)	219.81(152.56,310.12)	2.30(2.13,2.46)	0.75(0.56,0.98)	0.64(0.16,1.12)
Lao People's Democratic Republic	282.96(203.32,371.21)	2.48(2.43,2.52)	3123.44(2586.55,3733.76)	2.56(2.46,2.65)	452.85(324.06,615.32)	0.20(0.09,0.31)	4.64(2.82,7.02)	-1.17(-1.32,-1.03)
Latvia	191.76(132.46,258.36)	2.57(2.43,2.72)	2086.22(1686.86,2550.50)	2.52(2.39,2.65)	223.66(165.16,306.63)	2.47(2.12,2.82)	1.42(1.14,1.76)	1.77(-0.03,3.60)
Lebanon	461.26(340.93,604.42)	2.36(2.28,2.45)	5011.76(4196.34,5889.77)	2.59(2.53,2.64)	454.19(311.71,638.61)	0.91(0.83,1.00)	1.72(1.05,2.58)	-2.82(-3.09,-2.56)
Lesotho	182.12(128.85,242.63)	3.36(3.29,3.44)	2122.14(1743.68,2535.90)	3.31(3.25,3.38)	426.67(277.62,629.58)	4.77(4.11,5.42)	5.69(2.97,9.57)	5.78(4.72,6.85)
Liberia	213.53(146.62,293.54)	2.48(2.41,2.55)	3358.36(2841.62,3993.52)	2.75(2.66,2.85)	468.66(332.07,657.91)	2.04(1.86,2.23)	4.69(2.68,7.61)	1.41(1.13,1.70)
Libya	492.88(352.22,669.18)	3.62(3.40,3.84)	5107.76(4244.70,6098.32)	3.63(3.42,3.85)	456.86(312.40,634.27)	3.34(3.13,3.55)	2.34(1.34,3.83)	2.67(2.39,2.94)

Table S7 The ASIR, ASPR, ASDR and ASMR of of Diabetes mellitus type 2 in 204 countries and territories in 2021,and the EAPC from 1990 to 2021. (continued)

Diabetes mellitus type location	SAIR (1/100,000, 95%UI)	EAPC ASIR (EAPC, 95% CI)	SAPR (1/100,000, 95%UI)	EAPC SAPR (EAPC, 95% CI)	SADR (1/100,000, 95%UI)	EAPC SADR (EAPC, 95% CI)	SAMR (1/100,000, 95%UI)	EAPC SAMR (EAPC, 95% CI)
Lithuania	161.56(112.98,220.78)	2.46(2.35,2.57)	1775.33(1433.37,2165.82)	2.47(2.37,2.57)	157.28(107.57,225.59)	2.54(2.40,2.68)	0.58(0.46,0.72)	2.55(0.99,4.13)
Luxembourg	191.68(131.05,264.23)	2.43(2.40,2.46)	2870.15(2307.20,3489.77)	2.49(2.45,2.52)	182.45(113.64,269.13)	2.03(1.99,2.08)	0.10(0.08,0.13)	-3.46(-3.75,-3.18)
Madagascar	107.21(73.41,148.00)	1.81(1.77,1.85)	1681.12(1401.97,2009.29)	1.83(1.78,1.89)	353.01(245.63,483.62)	0.34(0.24,0.44)	4.36(2.60,6.77)	-0.35(-0.45,-0.24)
Malawi	68.65(47.66,93.94)	0.77(0.63,0.92)	1092.40(896.97,1301.03)	1.35(1.22,1.49)	255.36(178.74,356.28)	-0.38(-0.55,-0.21)	3.39(2.02,5.25)	-0.99(-1.22,-0.75)
Malaysia	273.36(193.75,365.08)	1.44(1.20,1.68)	3182.70(2631.60,3788.12)	1.80(1.57,2.03)	325.65(238.61,437.74)	0.82(0.71,0.93)	2.24(1.56,3.13)	-0.51(-0.82,-0.21)
Maldives	195.33(135.85,264.51)	1.34(1.17,1.51)	2325.95(1900.57,2795.29)	1.72(1.55,1.88)	223.38(156.52,313.71)	-1.78(-2.21,-1.36)	1.20(0.76,1.76)	-5.32(-5.73,-4.92)
Mali	322.84(213.18,462.31)	2.25(2.06,2.44)	5487.87(4548.00,6556.03)	2.62(2.34,2.91)	618.54(448.55,849.27)	1.47(1.25,1.68)	4.82(3.03,7.25)	0.16(-0.04,0.36)
Malta	249.34(166.85,346.18)	3.53(3.46,3.59)	3325.58(2663.52,4092.91)	3.84(3.81,3.87)	229.10(150.16,329.61)	2.68(2.57,2.79)	0.47(0.39,0.56)	-1.75(-2.03,-1.47)
Marshall Islands	1374.15(969.34,1825.18)	3.01(2.84,3.18)	19293.07(16264.74,22925.23)	3.30(3.13,3.47)	3370.49(2301.71,4684.49)	2.68(2.32,3.04)	43.53(23.94,68.31)	2.34(1.85,2.83)
Mauritania	170.96(124.13,221.41)	1.83(1.68,1.98)	2227.66(1902.11,2591.11)	1.75(1.65,1.85)	305.94(217.91,419.90)	0.77(0.63,0.90)	3.00(1.76,4.78)	-0.04(-0.29,0.22)
Mauritius	439.39(305.48,582.04)	2.14(2.02,2.26)	4931.27(4037.18,5910.67)	3.12(2.98,3.25)	881.17(730.29,1070.75)	3.20(2.82,3.58)	10.89(9.23,12.68)	3.22(2.55,3.90)
Mexico	535.49(386.68,712.59)	0.89(0.71,1.06)	6795.22(5731.98,7931.96)	1.13(0.92,1.33)	855.67(660.33,1084.67)	0.25(0.11,0.39)	6.95(5.74,8.28)	-0.83(-1.40,-0.26)
Micronesia (Federated States of)	855.46(629.20,1109.32)	3.02(2.78,3.26)	10721.16(9182.95,12467.83)	2.90(2.61,3.19)	1668.01(1198.06,2230.05)	1.95(1.65,2.24)	19.94(12.46,29.77)	1.32(0.98,1.67)
Monaco	179.94(122.16,247.58)	2.93(2.89,2.96)	2767.58(2222.96,3360.53)	2.80(2.77,2.82)	175.35(108.75,254.45)	2.65(2.63,2.68)	0.10(0.06,0.17)	-0.38(-0.52,-0.24)
Mongolia	186.89(135.14,244.86)	2.88(2.81,2.95)	2493.48(2090.02,2959.10)	2.87(2.81,2.93)	210.40(145.94,299.52)	2.25(2.15,2.35)	0.71(0.48,1.01)	0.24(-0.05,0.53)
Montenegro	228.77(164.01,308.82)	1.87(1.79,1.94)	1849.17(1482.94,2273.73)	1.66(1.54,1.77)	169.90(111.66,247.67)	1.40(1.23,1.58)	0.52(0.34,0.78)	0.23(-0.23,0.70)
Morocco	779.03(556.56,1039.24)	3.96(3.88,4.05)	8335.23(6881.24,9992.69)	4.13(4.01,4.24)	667.20(442.79,940.89)	3.73(3.61,3.85)	2.32(1.30,3.82)	2.39(2.15,2.63)
Mozambique	107.03(73.21,151.37)	2.53(2.49,2.57)	1625.40(1316.39,2001.43)	3.03(2.97,3.09)	334.38(226.42,478.82)	1.33(1.15,1.52)	4.18(2.30,6.79)	0.66(0.42,0.90)
Myanmar	354.16(261.77,456.32)	1.59(1.45,1.73)	3812.69(3228.46,4432.31)	1.58(1.42,1.75)	674.36(495.37,902.46)	-1.03(-1.21,-0.85)	8.21(5.19,11.96)	-2.04(-2.26,-1.83)
Namibia	151.12(105.46,208.17)	1.43(1.34,1.51)	1993.08(1649.35,2385.25)	1.75(1.67,1.83)	292.98(198.28,417.03)	0.47(0.15,0.79)	3.06(1.72,5.11)	-0.46(-0.96,0.04)
Nauru	953.14(681.57,1252.06)	2.08(1.89,2.27)	12232.56(10436.14,14318.97)	2.24(2.11,2.38)	2069.48(1469.55,2858.62)	1.40(1.19,1.61)	26.21(16.16,40.51)	0.95(0.69,1.21)
Nepal	324.33(228.10,444.69)	2.33(2.06,2.59)	5279.41(4487.23,6175.29)	2.59(2.27,2.91)	454.53(324.44,624.98)	1.76(1.44,2.08)	2.38(1.41,3.72)	0.12(-0.22,0.46)
Netherlands	140.20(93.03,197.23)	2.44(2.39,2.50)	1915.03(1493.68,2391.25)	2.86(2.76,2.96)	131.35(81.85,194.29)	1.86(1.73,1.99)	0.17(0.15,0.21)	-3.56(-3.85,-3.27)
New Zealand	173.91(128.31,222.84)	1.45(0.97,1.93)	2268.60(1973.29,2565.02)	2.93(2.34,3.53)	176.04(127.06,237.62)	1.50(1.12,1.88)	0.59(0.52,0.66)	-1.87(-2.27,-1.47)
Nicaragua	416.32(298.25,565.22)	1.92(1.83,2.01)	4879.22(4112.92,5821.49)	1.78(1.73,1.83)	539.34(389.81,731.60)	0.87(0.78,0.96)	3.32(2.21,4.69)	-0.49(-0.68,-0.31)
Niger	199.26(137.38,275.20)	2.19(2.14,2.24)	3124.70(2598.21,3706.27)	2.36(2.31,2.41)	331.97(231.74,458.65)	1.40(1.27,1.52)	2.30(1.33,3.74)	0.09(-0.16,0.33)

Table S7 The ASIR, ASPR, ASDR and ASMR of of Diabetes mellitus type 2 in 204 countries and territories in 2021,and the EAPC from 1990 to 2021. (continued)

Diabetes mellitus type location	SAIR (1/100,000, 95%UI)	EAPC ASIR (EAPC, 95% CI)	SAPR (1/100,000, 95%UI)	EAPC SAPR (EAPC, 95% CI)	SADR (1/100,000, 95%UI)	EAPC SADR (EAPC, 95% CI)	SAMR (1/100,000, 95%UI)	EAPC SAMR (EAPC, 95% CI)
Nigeria	134.11(92.17,184.40)	1.27(1.21,1.34)	1946.42(1598.86,2323.29)	1.56(1.49,1.63)	251.97(181.72,339.55)	0.29(0.18,0.39)	2.37(1.39,3.65)	-0.75(-0.90,-0.61)
Niue	1036.79(732.75,1370.41)	2.81(2.73,2.90)	13919.47(11840.19,16487.79)	2.97(2.86,3.07)	1767.40(1300.30,2335.57)	2.05(1.93,2.16)	16.89(10.47,24.77)	1.14(0.97,1.32)
North Macedonia	248.08(176.09,341.38)	1.96(1.85,2.06)	1809.05(1406.57,2271.22)	1.89(1.78,2.00)	188.80(125.53,270.39)	1.07(0.88,1.26)	0.93(0.58,1.40)	-0.58(-1.00,-0.17)
Northern Mariana Islands	511.48(368.40,672.57)	2.06(2.00,2.12)	7289.53(6080.68,8600.84)	2.06(2.00,2.13)	802.98(589.64,1064.83)	0.60(0.49,0.72)	6.65(4.59,9.26)	-0.87(-1.13,-0.61)
Norway	157.75(99.43,227.20)	0.75(0.65,0.86)	2199.82(1651.70,2768.84)	0.98(0.90,1.07)	144.18(91.96,211.66)	0.68(0.59,0.76)	0.16(0.15,0.17)	-2.25(-2.89,-1.61)
Oman	390.73(273.37,526.50)	1.24(0.89,1.58)	3947.13(3174.02,4866.90)	1.66(1.25,2.07)	349.85(240.10,478.45)	0.47(0.27,0.68)	1.70(1.06,2.59)	-1.45(-1.70,-1.19)
Pakistan	348.83(245.71,476.26)	2.74(2.51,2.97)	4582.74(3782.24,5461.21)	2.91(2.71,3.12)	541.57(397.25,711.97)	2.07(1.84,2.29)	4.59(2.93,6.79)	1.07(0.74,1.40)
Palau	998.90(726.25,1291.41)	2.50(2.40,2.60)	13066.18(11246.32,15108.98)	2.57(2.52,2.62)	1489.42(1104.56,1986.70)	1.79(1.69,1.88)	12.33(7.96,17.88)	0.80(0.60,1.00)
Palestine	381.62(274.68,501.33)	2.80(2.68,2.92)	3792.89(3128.17,4530.47)	2.61(2.55,2.68)	373.74(270.57,497.16)	1.28(1.17,1.39)	2.32(1.56,3.23)	-0.55(-0.69,-0.40)
Panama	348.24(248.52,464.45)	2.21(2.11,2.30)	4302.96(3588.79,5086.54)	2.05(2.00,2.09)	468.72(345.07,630.73)	1.14(1.00,1.29)	2.77(2.12,3.51)	-0.36(-0.68,-0.04)
Papua New Guinea	620.48(447.90,807.75)	2.93(2.88,2.97)	8518.57(7207.26,9981.11)	2.75(2.71,2.79)	1198.19(872.35,1596.75)	1.08(1.03,1.13)	12.88(8.44,18.95)	0.06(-0.01,0.14)
Paraguay	294.14(217.84,377.95)	1.95(1.77,2.13)	2811.39(2323.39,3358.64)	1.82(1.72,1.92)	446.89(332.36,589.04)	1.25(1.08,1.43)	4.56(3.01,6.60)	0.90(0.63,1.16)
Peru	133.66(95.91,176.49)	1.60(1.37,1.83)	1658.37(1392.35,1961.99)	1.76(1.55,1.97)	199.16(145.34,267.55)	0.94(0.68,1.19)	1.67(1.07,2.49)	0.08(-0.26,0.43)
Philippines	165.99(117.54,224.39)	1.72(1.53,1.91)	1887.67(1544.26,2254.26)	0.75(0.43,1.07)	370.19(294.60,461.65)	0.87(0.69,1.06)	4.83(3.73,6.14)	0.95(0.80,1.09)
Poland	180.09(126.51,243.75)	0.70(0.61,0.79)	1503.90(1185.41,1861.10)	0.77(0.69,0.84)	140.67(99.11,193.26)	0.35(0.13,0.57)	0.46(0.40,0.53)	-1.28(-2.10,-0.44)
Portugal	290.05(191.59,412.42)	2.46(2.26,2.66)	4431.53(3559.53,5412.94)	2.78(2.73,2.84)	291.57(189.46,418.51)	1.70(1.61,1.79)	0.38(0.32,0.45)	-3.63(-4.02,-3.25)
Puerto Rico	516.44(366.67,687.66)	2.30(2.12,2.48)	5974.49(4918.17,7214.84)	2.59(2.50,2.67)	614.21(427.11,868.52)	1.25(1.18,1.32)	2.45(1.87,3.09)	-1.63(-1.94,-1.33)
Qatar	708.42(477.24,1051.05)	2.77(2.68,2.86)	6262.71(5006.11,7840.05)	2.87(2.82,2.92)	513.49(343.83,732.54)	1.35(1.13,1.57)	1.73(1.13,2.54)	-1.65(-2.22,-1.09)
Republic of Korea	410.29(290.23,546.55)	3.23(2.92,3.55)	5968.98(5151.39,6849.71)	4.54(4.11,4.98)	413.19(272.89,600.90)	2.51(2.09,2.94)	0.49(0.35,0.67)	-4.38(-4.59,-4.16)
Republic of Moldova	241.78(168.05,322.30)	2.34(2.13,2.55)	2689.36(2168.07,3242.38)	2.32(2.12,2.51)	246.06(169.90,345.14)	2.08(1.91,2.25)	0.97(0.78,1.19)	0.33(-1.33,2.01)
Romania	149.14(107.19,201.16)	1.47(1.38,1.56)	1291.17(1013.03,1623.97)	1.26(1.19,1.33)	116.79(76.44,169.16)	0.65(0.56,0.74)	0.33(0.27,0.40)	-1.94(-2.39,-1.49)
Russian Federation	168.33(117.02,229.01)	2.32(2.26,2.39)	1669.43(1321.12,2048.34)	1.74(1.66,1.82)	156.27(113.52,211.45)	1.74(1.55,1.94)	0.69(0.61,0.78)	1.12(0.07,2.19)
Rwanda	70.11(47.63,96.16)	0.07(-0.14,0.28)	1068.69(867.47,1287.57)	0.43(0.20,0.65)	239.12(158.18,351.79)	-2.93(-3.40,-2.46)	3.12(1.65,5.29)	-3.96(-4.48,-3.43)
Saint Kitts and Nevis	482.40(342.03,650.48)	1.26(1.20,1.32)	5341.83(4364.74,6449.65)	1.22(1.12,1.32)	567.53(403.88,797.35)	-0.92(-1.20,-0.65)	2.53(1.84,3.39)	-4.28(-4.71,-3.84)
Saint Lucia	644.63(468.23,838.87)	1.33(1.27,1.40)	7693.60(6571.69,9052.38)	1.70(1.63,1.77)	898.68(660.89,1218.78)	-0.12(-0.28,0.04)	5.31(4.09,6.86)	-2.52(-2.83,-2.20)

Table S7 The ASIR, ASPR, ASDR and ASMR of of Diabetes mellitus type 2 in 204 countries and territories in 2021,and the EAPC from 1990 to 2021. (continued)

**Diabetes mellitus type location**	**SAIR (1/100,000, 95%UI)**	**EAPC ASIR (EAPC, 95% CI)**	**SAPR (1/100,000, 95%UI)**	**EAPC SAPR (EAPC, 95% CI)**	**SADR (1/100,000, 95%UI)**	**EAPC SADR (EAPC, 95% CI)**	**SAMR (1/100,000, 95%UI)**	**EAPC SAMR (EAPC, 95% CI)**
Saint Vincent and the Grenadines	650.95(454.03,867.07)	1.84(1.79,1.88)	7469.78(6173.07,8953.02)	1.92(1.84,2.00)	1042.14(794.84,1350.97)	-0.13(-0.30,0.04)	8.60(6.91,10.67)	-1.81(-2.08,-1.54)
Samoa	937.14(670.71,1237.89)	2.81(2.65,2.96)	12732.28(10788.43,15049.46)	3.01(2.81,3.20)	1581.29(1127.62,2130.47)	2.55(2.46,2.64)	15.39(9.50,23.49)	2.10(2.01,2.19)
San Marino	187.30(126.32,260.50)	2.79(2.76,2.82)	2818.69(2258.55,3438.25)	2.63(2.60,2.67)	178.85(114.22,261.36)	2.42(2.38,2.46)	0.11(0.05,0.19)	-0.89(-1.24,-0.53)
Sao Tome and Principe	237.72(162.50,327.96)	2.59(2.57,2.60)	4254.29(3608.12,4970.97)	3.02(3.00,3.05)	343.11(235.49,485.07)	2.35(2.21,2.48)	1.07(0.65,1.76)	0.22(-0.29,0.73)
Saudi Arabia	494.59(356.30,656.16)	2.73(2.59,2.86)	5411.25(4370.28,6498.72)	3.21(3.03,3.39)	512.17(372.78,688.46)	2.29(2.18,2.40)	3.76(2.35,5.63)	1.01(0.88,1.14)
Senegal	254.40(172.80,346.08)	2.33(2.18,2.48)	3841.36(3270.18,4500.18)	2.33(2.20,2.46)	490.18(356.71,662.04)	1.60(1.38,1.81)	4.47(2.77,6.98)	0.88(0.56,1.20)
Serbia	234.61(166.53,310.88)	1.51(1.46,1.57)	1786.34(1411.36,2235.86)	1.09(1.01,1.17)	173.43(116.00,248.52)	0.54(0.42,0.67)	0.68(0.47,0.92)	-1.11(-1.45,-0.78)
Seychelles	426.59(288.28,593.29)	4.04(3.86,4.22)	4840.83(3947.74,5867.33)	4.07(3.95,4.19)	483.23(344.62,660.69)	3.34(3.25,3.44)	2.68(1.89,3.64)	2.06(1.76,2.35)
Sierra Leone	215.22(147.39,304.81)	2.79(2.69,2.88)	3417.40(2844.66,4101.05)	3.00(2.90,3.10)	416.14(294.23,583.27)	2.76(2.49,3.03)	3.55(2.05,5.66)	2.43(1.97,2.88)
Singapore	317.39(225.02,429.34)	0.35(0.24,0.46)	4483.05(3761.48,5327.09)	1.78(1.59,1.98)	291.79(187.04,428.01)	0.64(0.42,0.86)	0.18(0.15,0.21)	-6.46(-7.12,-5.80)
Slovakia	161.18(114.59,216.66)	1.43(1.35,1.51)	1347.12(1049.42,1670.68)	1.44(1.36,1.51)	122.38(79.88,178.34)	1.08(1.01,1.14)	0.35(0.23,0.52)	-0.55(-0.78,-0.32)
Slovenia	157.41(110.76,211.63)	1.53(1.42,1.64)	1390.27(1076.43,1725.69)	1.52(1.41,1.63)	123.49(81.16,183.28)	0.91(0.84,0.98)	0.32(0.24,0.40)	-1.61(-1.82,-1.41)
Solomon Islands	546.53(412.67,695.56)	2.42(2.35,2.49)	7000.75(6021.44,8095.75)	2.68(2.62,2.74)	1629.22(1149.47,2235.40)	1.93(1.86,2.00)	24.32(15.45,36.68)	1.66(1.56,1.75)
Somalia	121.47(82.22,169.55)	1.85(1.81,1.89)	1805.06(1472.68,2191.45)	2.17(2.14,2.20)	411.65(275.33,589.87)	0.52(0.45,0.60)	5.43(2.98,8.85)	-0.13(-0.25,-0.01)
South Africa	206.53(140.01,284.10)	1.23(1.16,1.29)	2788.46(2294.32,3325.83)	1.16(1.06,1.26)	462.59(374.00,567.07)	0.95(0.57,1.33)	5.41(4.36,6.53)	1.08(0.42,1.74)
South Sudan	98.78(67.57,137.14)	1.97(1.93,2.01)	1556.59(1285.49,1879.27)	2.28(2.24,2.32)	367.00(248.53,528.16)	1.15(0.86,1.45)	4.89(2.78,7.85)	0.64(0.24,1.03)
Spain	260.54(173.17,365.70)	2.30(2.15,2.45)	3712.08(3010.56,4480.53)	3.05(2.66,3.44)	259.49(166.75,380.45)	2.25(1.87,2.62)	0.20(0.17,0.23)	-3.72(-3.89,-3.54)
Sri Lanka	400.28(281.15,537.20)	3.22(3.01,3.42)	4524.24(3727.25,5418.29)	3.12(2.86,3.38)	506.81(355.99,718.59)	2.54(2.30,2.77)	3.32(1.84,5.31)	1.26(1.01,1.50)
Sudan	397.41(290.73,510.84)	2.84(2.75,2.93)	4479.70(3785.58,5198.73)	3.07(2.97,3.16)	360.66(251.19,500.09)	2.50(2.45,2.55)	1.86(0.99,3.14)	0.90(0.77,1.03)
Suriname	620.13(450.25,821.59)	2.78(2.70,2.87)	7257.35(6135.07,8505.94)	2.80(2.74,2.85)	865.04(631.47,1180.13)	1.76(1.62,1.89)	5.35(3.50,7.63)	0.14(-0.19,0.46)
Sweden	181.77(117.20,259.54)	1.73(1.63,1.82)	2489.80(1912.41,3106.07)	1.56(1.45,1.68)	159.34(104.23,229.04)	1.00(0.88,1.12)	0.27(0.22,0.33)	-2.19(-2.47,-1.91)
Switzerland	266.60(182.93,368.16)	2.64(2.49,2.79)	3984.88(3241.99,4789.75)	2.64(2.48,2.79)	252.00(158.64,371.80)	2.10(1.93,2.28)	0.14(0.12,0.17)	-3.87(-4.12,-3.62)
Syrian Arab Republic	385.70(276.14,518.50)	2.20(2.16,2.24)	4266.44(3492.99,5084.31)	2.12(2.07,2.17)	354.26(243.62,491.93)	0.90(0.75,1.06)	1.39(0.85,2.15)	-1.94(-2.37,-1.51)
Taiwan (Province of China)	228.77(165.08,300.46)	1.01(0.86,1.15)	2619.69(2169.30,3110.08)	0.92(0.76,1.08)	231.58(160.49,323.31)	-0.36(-0.59,-0.14)	0.88(0.73,1.06)	-3.27(-3.77,-2.77)

Table S7 The ASIR, ASPR, ASDR and ASMR of of Diabetes mellitus type 2 in 204 countries and territories in 2021,and the EAPC from 1990 to 2021. (continued)

Diabetes mellitus type location	SAIR (1/100,000, 95%UI)	EAPC ASIR (EAPC, 95% CI)	SAPR (1/100,000, 95%UI)	EAPC SAPR (EAPC, 95% CI)	SADR (1/100,000, 95%UI)	EAPC SADR (EAPC, 95% CI)	SAMR (1/100,000, 95%UI)	EAPC SAMR (EAPC, 95% CI)
Tajikistan	225.38(160.98,300.26)	3.10(2.99,3.21)	2456.35(2038.37,2920.96)	2.61(2.51,2.71)	250.45(177.13,344.58)	1.04(0.82,1.26)	1.46(0.88,2.25)	-1.18(-1.61,-0.76)
Thailand	195.77(139.34,261.92)	1.98(1.90,2.05)	2299.01(1889.74,2752.51)	2.27(2.16,2.38)	301.46(217.41,401.35)	0.38(-0.04,0.80)	2.79(1.78,4.12)	-1.11(-1.72,-0.50)
Timor-Leste	227.22(159.72,303.01)	3.28(3.22,3.35)	2559.86(2088.92,3052.60)	3.52(3.47,3.58)	292.67(204.02,403.73)	1.78(1.46,2.09)	2.23(1.31,3.52)	-0.05(-0.61,0.52)
Togo	131.54(92.93,177.68)	1.84(1.73,1.95)	2055.20(1733.58,2431.78)	2.13(2.03,2.23)	303.05(211.56,416.19)	1.24(1.16,1.32)	3.18(1.84,5.01)	0.64(0.46,0.82)
Tokelau	974.17(711.63,1280.91)	2.20(2.04,2.37)	12336.68(10414.51,14582.10)	2.11(2.00,2.21)	1600.35(1164.50,2147.10)	1.25(1.15,1.35)	15.64(10.27,23.43)	0.38(0.24,0.52)
Tonga	874.98(638.52,1140.55)	2.38(2.31,2.46)	10810.81(9147.42,12736.79)	2.35(2.30,2.40)	1439.86(1049.50,1947.36)	1.04(0.98,1.11)	14.96(9.72,22.05)	0.06(-0.06,0.19)
Trinidad and Tobago	662.36(467.07,863.95)	0.95(0.91,0.99)	7159.58(5932.81,8530.54)	1.62(1.52,1.72)	1050.14(797.62,1385.32)	-0.42(-0.58,-0.25)	9.22(6.63,12.47)	-2.08(-2.38,-1.77)
Tunisia	426.15(304.99,563.38)	3.03(2.82,3.23)	4688.97(3867.12,5585.04)	3.25(3.14,3.36)	365.96(247.88,525.35)	2.87(2.78,2.97)	1.07(0.65,1.68)	1.28(1.19,1.37)
Turkmenistan	192.11(139.24,249.14)	3.10(2.98,3.23)	2409.69(2009.21,2841.71)	3.41(3.24,3.57)	310.47(234.36,409.43)	3.26(2.96,3.56)	2.76(2.01,3.85)	2.95(2.29,3.61)
Tuvalu	699.40(509.90,910.62)	2.43(2.38,2.48)	8762.31(7492.04,10249.80)	2.44(2.39,2.50)	1415.40(1016.72,1914.71)	1.14(1.07,1.20)	17.35(11.04,26.46)	0.45(0.37,0.53)
Turkey	300.60(220.59,384.60)	3.31(2.91,3.71)	3298.82(2751.23,3877.80)	3.13(2.78,3.47)	300.69(203.80,423.70)	1.20(0.81,1.58)	1.05(0.68,1.54)	-2.76(-3.09,-2.43)
Uganda	80.50(55.96,110.09)	1.68(1.60,1.75)	1258.11(1039.96,1511.09)	2.03(1.98,2.08)	259.04(179.45,363.51)	0.44(0.04,0.83)	3.16(1.81,5.01)	-0.33(-0.86,0.21)
Ukraine	171.27(115.81,236.51)	1.99(1.83,2.16)	1977.63(1550.54,2438.20)	1.70(1.51,1.89)	165.36(114.17,230.72)	0.93(0.67,1.19)	0.45(0.25,0.71)	-2.32(-3.07,-1.57)
United Arab Emirates	401.70(285.40,530.22)	2.18(2.10,2.26)	3937.25(3185.64,4762.55)	2.46(2.33,2.58)	297.80(203.25,423.34)	1.28(1.13,1.43)	0.91(0.58,1.35)	-1.89(-2.33,-1.45)
United Kingdom	326.96(233.94,434.14)	3.83(3.56,4.10)	5783.94(4869.59,6779.37)	4.04(3.80,4.27)	342.73(223.72,490.19)	3.44(3.22,3.65)	0.27(0.26,0.28)	-1.66(-1.96,-1.36)
United Republic of Tanzania	96.28(66.29,132.43)	2.54(2.45,2.62)	1386.40(1122.35,1679.25)	2.79(2.67,2.90)	266.95(188.91,365.47)	0.73(0.66,0.81)	3.11(1.89,4.71)	-0.22(-0.33,-0.11)
United States of America	330.94(249.83,422.90)	2.78(2.73,2.83)	3663.13(3170.30,4195.91)	3.84(3.72,3.95)	281.09(206.88,374.40)	2.07(2.01,2.14)	1.18(1.12,1.25)	-1.12(-1.33,-0.90)
United States Virgin Islands	652.29(455.86,878.57)	1.97(1.87,2.08)	7353.95(6097.87,8771.18)	2.22(2.08,2.36)	715.61(484.76,1006.63)	1.32(1.20,1.44)	2.09(1.19,3.38)	-1.09(-1.46,-0.71)
Uruguay	210.26(149.36,280.62)	3.27(3.13,3.41)	1941.21(1553.02,2396.59)	3.94(3.76,4.13)	173.16(124.68,235.79)	1.70(1.58,1.81)	0.95(0.81,1.12)	-1.24(-1.49,-0.99)
Uzbekistan	282.54(200.64,382.69)	3.80(3.58,4.02)	3069.76(2552.67,3699.41)	3.43(3.25,3.60)	317.33(229.46,432.85)	2.44(2.13,2.75)	1.92(1.39,2.52)	0.81(0.17,1.45)
Vanuatu	633.29(463.93,826.32)	3.05(2.99,3.10)	8511.28(7312.04,9867.39)	3.11(3.08,3.13)	1261.78(919.09,1680.12)	1.73(1.64,1.81)	14.60(9.23,21.53)	0.91(0.78,1.04)

Table S7 The ASIR, ASPR, ASDR and ASMR of of Diabetes mellitus type 2 in 204 countries and territories in 2021,and the EAPC from 1990 to 2021. (continued)

**Diabetes mellitus type**<br>**location**	**SAIR**<br>**(1/100,000, 95%UI)**	**EAPC ASIR**<br>**(EAPC, 95% CI)**	**SAPR**<br>**(1/100,000, 95%UI)**	**EAPC SAPR**<br>**(EAPC, 95% CI)**	**SADR**<br>**(1/100,000, 95%UI)**	**EAPC SADR**<br>**(EAPC, 95% CI)**	**SAMR**<br>**(1/100,000, 95%UI)**	**EAPC SAMR**<br>**(EAPC, 95% CI)**
Venezuela (Bolivarian Republic of)	326.57(232.47,438.23)	1.78(1.67,1.89)	4433.64(3720.51,5196.03)	2.20(2.08,2.32)	526.02(388.11,696.31)	1.33(1.01,1.66)	3.84(2.74,5.22)	0.14(-0.50,0.78)
Viet Nam	147.48(107.88,192.09)	1.30(1.07,1.52)	1717.33(1413.69,2021.38)	1.49(1.26,1.72)	252.51(183.79,343.41)	0.51(0.29,0.74)	2.64(1.67,4.09)	-0.14(-0.35,0.07)
Yemen	283.75(204.77,378.70)	1.75(1.29,2.22)	3123.49(2579.49,3745.35)	1.60(1.00,2.19)	267.40(180.29,384.17)	1.24(0.81,1.66)	1.28(0.68,2.38)	0.35(0.21,0.49)
Zambia	160.38(107.16,221.70)	1.71(1.62,1.80)	2857.09(2383.81,3373.05)	2.15(2.09,2.20)	412.63(293.76,563.22)	-0.11(-0.28,0.06)	3.81(2.23,6.05)	-1.57(-1.80,-1.35)
Zimbabwe	173.60(119.00,239.28)	2.57(2.53,2.62)	2192.08(1803.17,2625.35)	2.32(2.26,2.37)	390.98(270.20,549.66)	3.82(3.06,4.59)	4.73(2.82,7.53)	5.12(3.78,6.48)

T2DM type 2 diabetes mellitus, DALYs disability-adjusted life-years, ASIR age-standardized incidence rate, ASPR age-standardized prevalence rate, ASMR age-standardized mortality rate, ASDR age-standardized DALYs rate, EAPC estimated annual percentage change, CI confdence interval, UI uncertainty intervals

Table S8 Percentage of uterine cancer,T2DM deaths and DALYs attributed to risk factors in 1990 and 2021

location	Leading risk	T2DM<br>Percent of DALYs<br>1990 (95% UI)	Percent of DALYs<br>2021 (95% UI)	Percent of death<br>1990 (95% UI)	Percent of death<br>2021 (95% UI)	Uterine cancer<br>Percent of DALYs<br>1990 (95% UI)	Percent of DALYs<br>2021 (95% UI)	Percent of death<br>1990 (95% UI)	Percent of death<br>2021 (95% UI)
Global	Metabolic risks	100.00 (100.00, 100.00)	100.00 (100.00, 100.00)	100.00 (100.00, 100.00)	100.00 (100.00, 100.00)	18.41 (24.47, 13.11)	30.57 (39.69, 22.15)	18.65 (24.82, 13.29)	30.76 (39.95, 22.29)
	High body-mass index	49.47 (65.55, 26.12)	62.44(78.73, 35.24)	50.86 (66.69, 26.81)	62.87 (79.26, 35.28)	18.41 (24.47, 13.11)	30.57 (39.69, 22.15)	18.65 (24.82, 13.29)	30.76 (39.95, 22.29)
	High fasting plasma glucose	100.00 (100.00, 100.00)	100.00 (100.00, 100.00)	100.00 (100.00, 100.00)	100.00 (100.00, 100.00)	-	-	-	-
High-middle SDI	Metabolic risks	100.00 (100.00, 100.00)	100.00 (100.00, 100.00)	100.00 (100.00, 100.00)	100.00 (100.00, 100.00)	22.06 (29.31, 15.48)	33.54 (43.96, 24.28)	22.43 (29.84, 15.72)	33.88 (44.40, 24.55)
	High body-mass index	52.86 (69.66, 28.39)	66.26 (82.78, 38.22)	56.54 (73.57, 30.49)	69.30 (85.48, 41.10)	22.06 (29.31, 15.48)	33.54 (43.96, 24.28)	22.43 (29.84, 15.72)	33.88 (44.40, 24.55)
	High fasting plasma glucose	100.00 (100.00, 100.00)	100.00 (100.00, 100.00)	100.00 (100.00, 100.00)	100.00 (100.00, 100.00)	-	-	-	-
High SDI	Metabolic risks	100.00 (100.00, 100.00)	100.00 (100.00, 100.00)	100.00 (100.00, 100.00)	100.00 (100.00, 100.00)	25.13 (33.27, 17.84)	36.60 (46.62, 26.54)	25.13 (33.26, 17.89)	36.49 (46.44, 26.43)
	High body-mass index	60.24 (77.76, 33.71)	69.95 (85.46, 42.30)	62.65 (80.13, 35.39)	74.84 (89.37, 47.27)	25.13 (33.27, 17.84)	36.60 (46.62, 26.54)	25.13 (33.26, 17.89)	36.49 (46.44, 26.43)
	High fasting plasma glucose	100.00 (100.00, 100.00)	100.00 (100.00, 100.00)	100.00 (100.00, 100.00)	100.00 (100.00, 100.00)	-	-	-	-
Middle SDI	Metabolic risks	100.00 (100.00, 100.00)	100.00 (100.00, 100.00)	100.00 (100.00, 100.00)	100.00 (100.00, 100.00)	14.19 (19.00, 10.24)	29.80（38.59，21.47）	14.48 (19.35, 10.42)	30.14 (39.00, 21.71)
	High body-mass index	51.05 (67.15, 27.16)	65.26 (81.60, 37.26)	53.77 (69.98, 28.74)	66.38 (82.97, 38.09)	14.19 (19.00, 10.24)	29.80（38.59，21.47）	14.48 (19.35, 10.42)	30.14 (39.00, 21.71)
	High fasting plasma glucose	100.00 (100.00, 100.00)	100.00 (100.00, 100.00)	100.00 (100.00, 100.00)	100.00 (100.00, 100.00)	-	-	-	
Low-middle SDI	Metabolic risks	100.00 (100.00, 100.00)	100.00 (100.00, 100.00)	100.00 (100.00, 100.00)	100.00 (100.00, 100.00)	15.59 (20.35, 11.38)	26.92 (35.34, 18.81)	15.95 (20.79, 11.61)	27.27 (35.75, 19.04)

Table S8 Percentage of uterine cancer,T2DM deaths and DALYs attributed to risk factors in 1990 and 2021(continued)

location	Leading risk	T2DM Percent of DALYs 1990 (95% UI)	T2DM Percent of DALYs 2021 (95% UI)	T2DM Percent of death 1990 (95% UI)	T2DM Percent of death 2021 (95% UI)	Uterine cancer Percent of DALYs 1990 (95% UI)	Uterine cancer Percent of DALYs 2021 (95% UI)	Uterine cancer Percent of death 1990 (95% UI)	Uterine cancer Percent of death 2021 (95% UI)
	High body-mass index	43.43 (59.04, 22.20)	59.19 (75.85, 32.48)	45.52 (61.74, 23.51)	60.94 (77.91, 33.51)	15.59 (20.35, 11.38)	26.92 (35.34, 18.81)	15.95 (20.79, 11.61)	27.27 (35.75, 19.04)
	High fasting plasma glucose	100.00 (100.00, 100.00)	100.00 (100.00, 100.00)	100.00 (100.00, 100.00)	100.00 (100.00, 100.00)	-	-	-	-
Low SDI	Metabolic risks	100.00 (100.00, 100.00)	100.00 (100.00, 100.00)	100.00 (100.00, 100.00)	100.00 (100.00, 100.00)	13.53 (17.65, 9.62)	23.09 (30.56,15.66)	13.83 (17.99, 9.80)	23.48(31.10, 15.90)
	High body-mass index	40.94 (56.15, 19.91)	52.00 (68.18, 27.25)	42.52 (58.42, 20.62)	53.83 (70.23, 28.33)	13.53 (17.65, 9.62)	23.09 (30.56,15.66)	13.83 (17.99, 9.80)	23.48(31.10, 15.90)
	High fasting plasma glucose	100.00 (100.00, 100.00)	100.00 (100.00, 100.00)	100.00 (100.00, 100.00)	100.00 (100.00, 100.00)	-	-	-	-

PCOS polycystic ovary syndrome, T2DM type 2 diabetes mellitus, DALYs disability-adjusted life-years

A

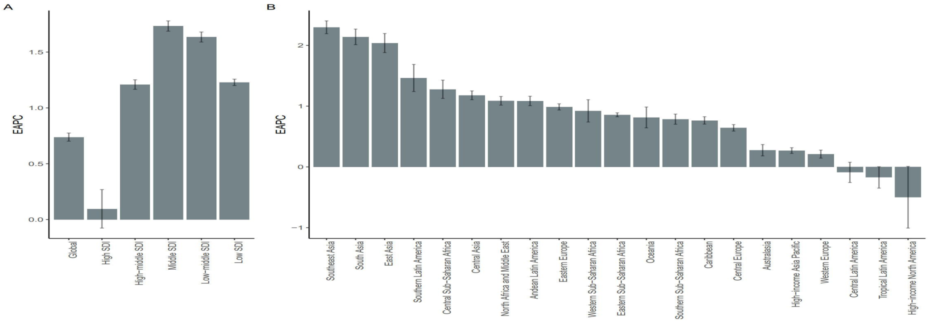

B

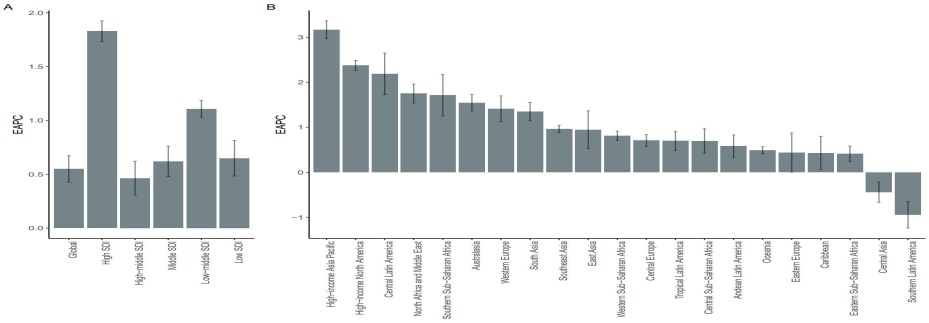

C

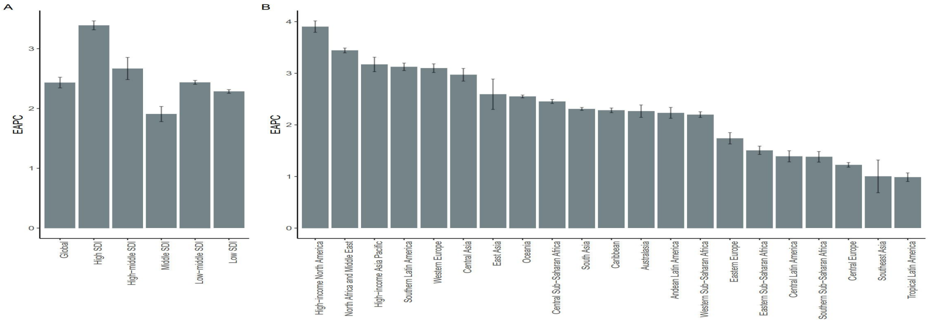

Fig. S1 The EAPC of ASPR for PCOS,Uterine cancer,T2DM in global and 21 regions. ASPR age-standardized prevalence rate, EAPC estimated annual percentage change, A PCOS Polycystic ovary syndrome , B Uterine cancer ,C T2DM type 2 diabetes mellitus

A

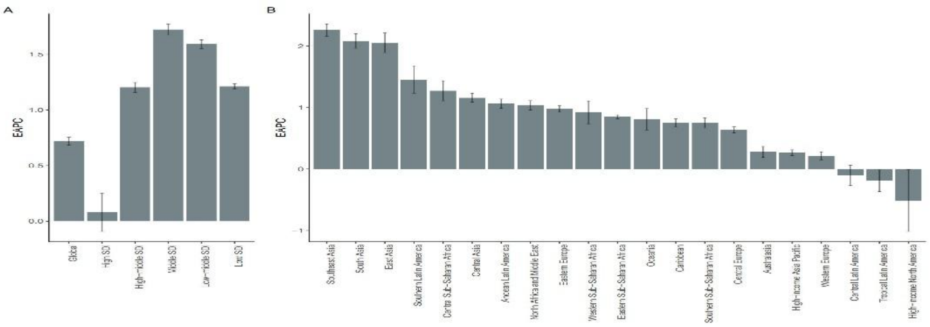

B

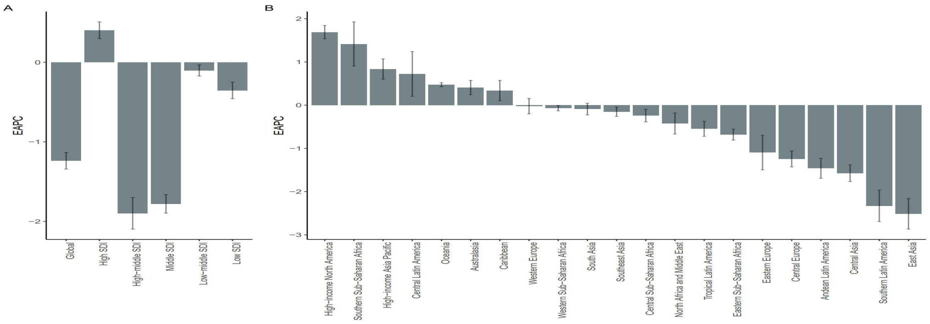

C

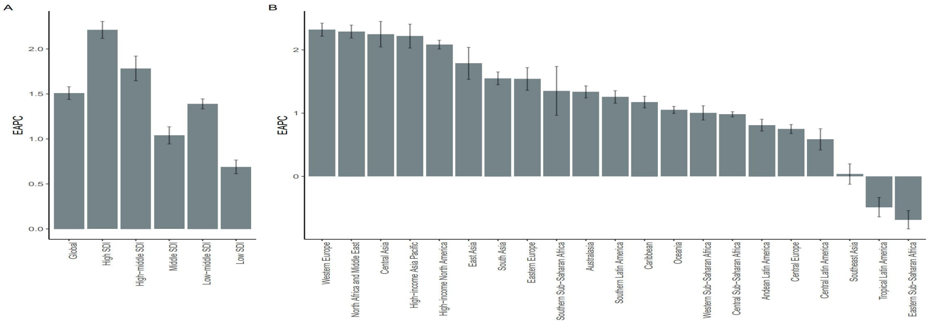

Fig. S2 The EAPC of ASDR for PCOS,Uterine cancer,T2DM in global and 21 regions. ASDR age-standardized DALYs , EAPC estimated annual percentage change, A PCOS Polycystic ovary syndrome , B Uterine cancer ,C T2DM type 2 diabetes mellitus

A

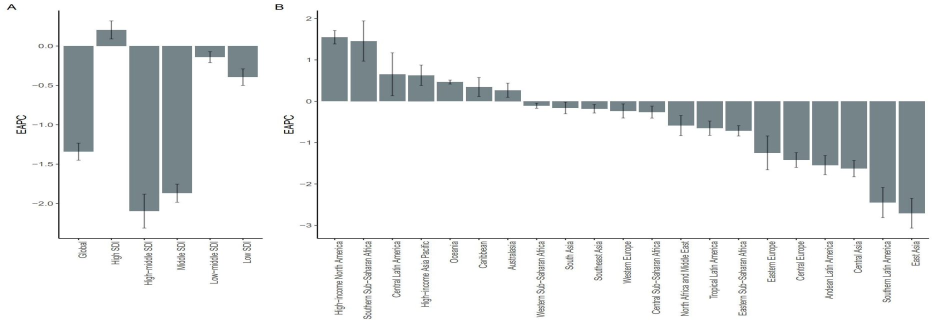

B

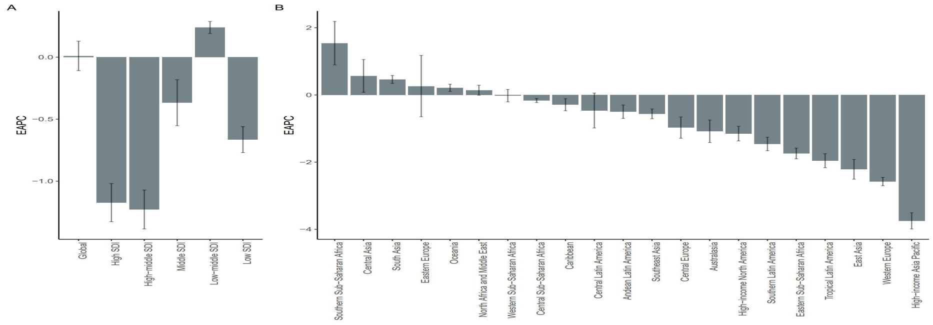

Fig. S3 The EAPC of ASMR for Uterine cancer,T2DM in global and 21 regions. ASMR age- standardized Mortality rate , EAPC estimated annual percentage change, A Uterine cancer ,B T2DM type 2 diabetes mellitus

A r=0.4128, p=0.000e+00"

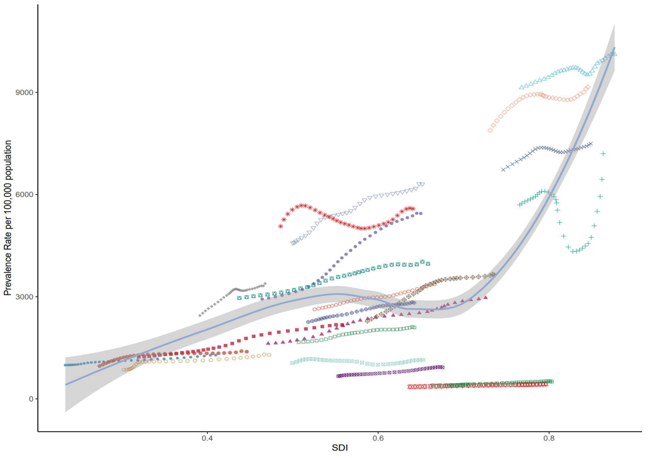

B r=0.7307, p=0.000e+00

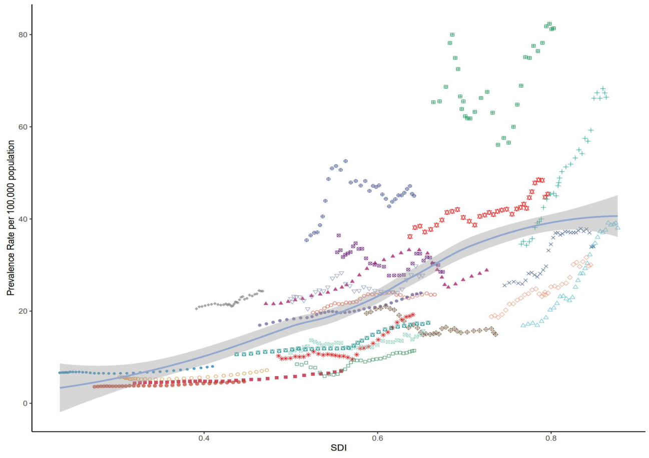

C r=-0.1446, p=1.200e-04

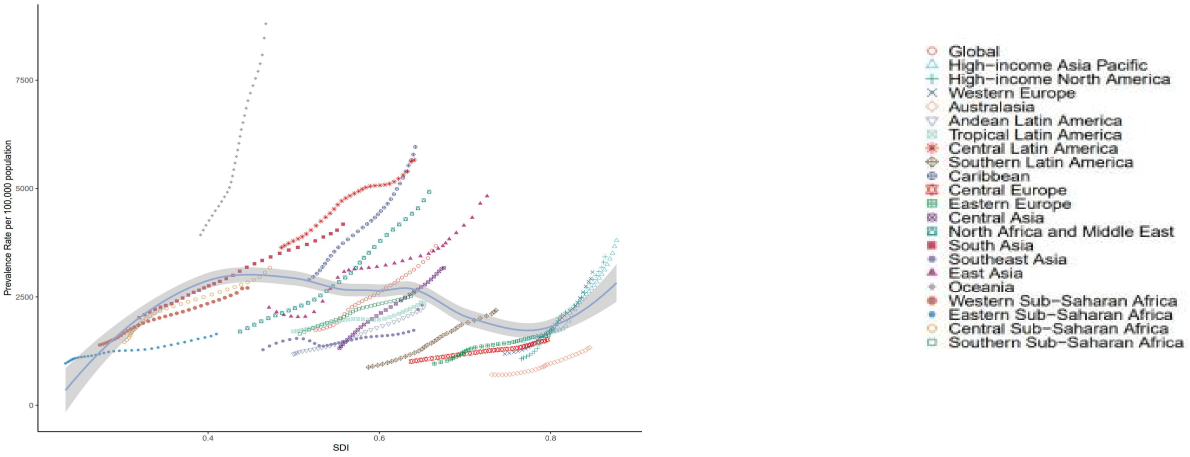

Fig. S4. ASPR for PCOS,Uterine cancer,T2DM in global and 21 regions. From 1990 to 2021. ASPR age-standardized prevalence rate incidence rate, A PCOS Polycystic ovary syndrome , B Uterine cancer ,C T2DM type 2 diabetes mellitus

A r=0.4150, p=0.000e+00

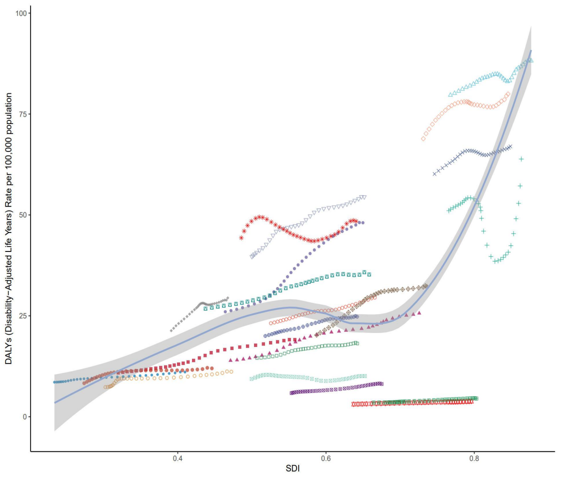

B r=0.1398, p=2.001e-04

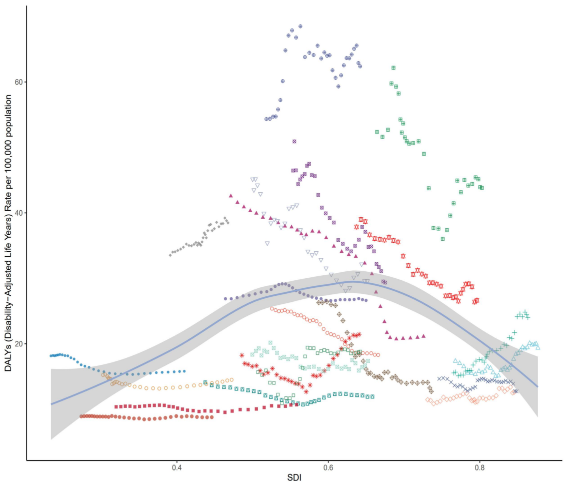

C r=-0.6300, p=0.000e+00

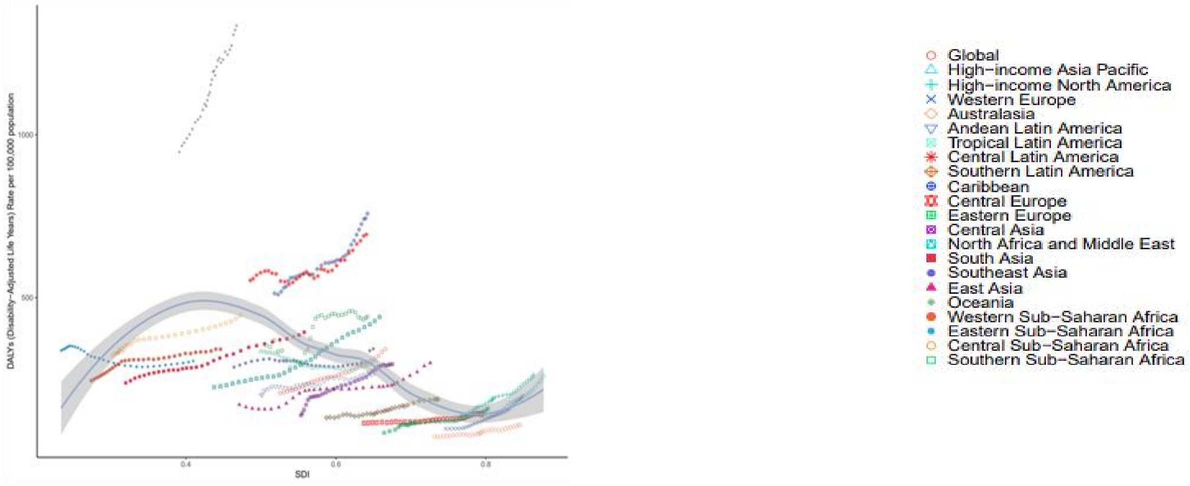

Fig. S5. ASDR for PCOS,Uterine cancer,T2DM in global and 21 regions. From 1990 to 2021. ASDR age-standardized DALYs rate, A PCOS Polycystic ovary syndrome , B Uterine cancer ,C T2DM type 2 diabetes mellitus

A r=0.0702, p=6.277e-02

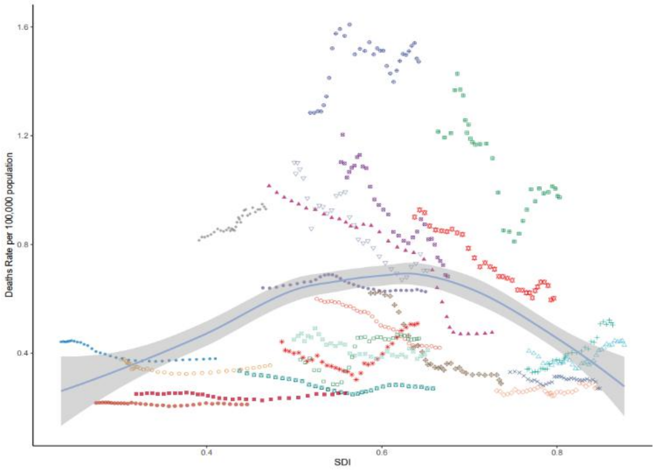

B r =-0.7500, p=0.000e+00

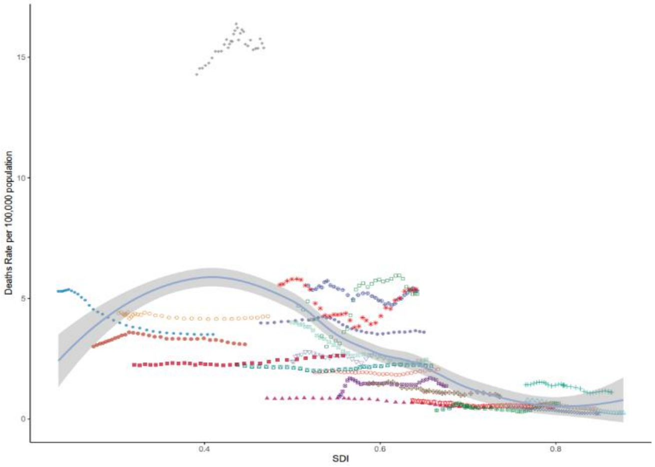

Fig. S6. ASMR for Uterine cancer,T2DM in global and 21 regions. From 1990 to 2021. ASMR age-standardized mortality rate, A Uterine cancer ,B T2DM type 2 diabetes mellitus

A

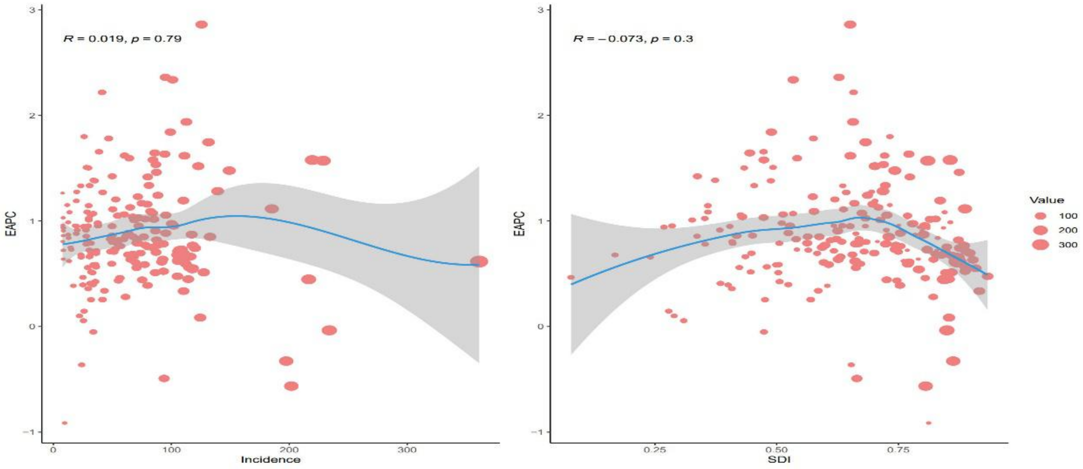

B

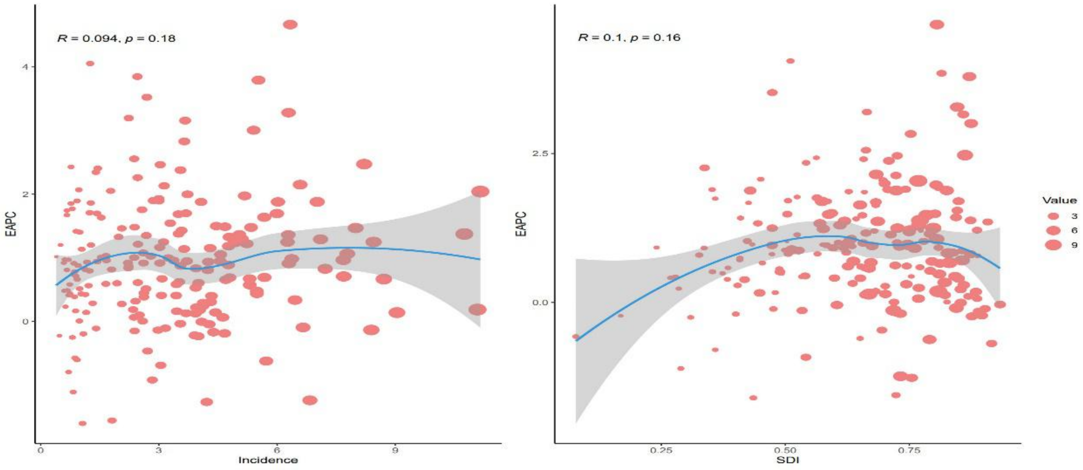

C

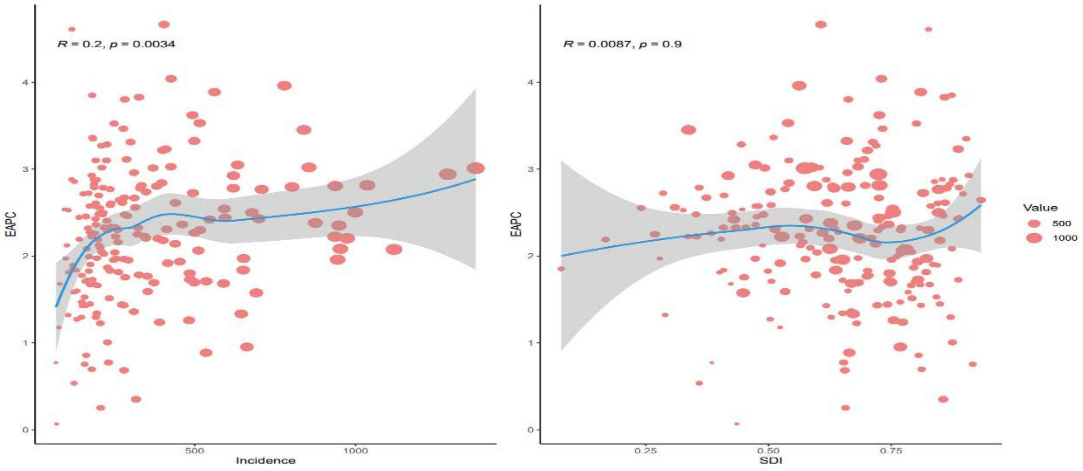

Fig. S7 The EAPC of ASIR for PCOS,uterine cancer,T2DM in 204 countries and territories by SDI. ASIR age- standardized incidence rate, SDI sociodemographic index, A PCOS Polycystic ovary syndrome , B Uterine cancer ,C T2DM type 2 diabetes mellitus

A

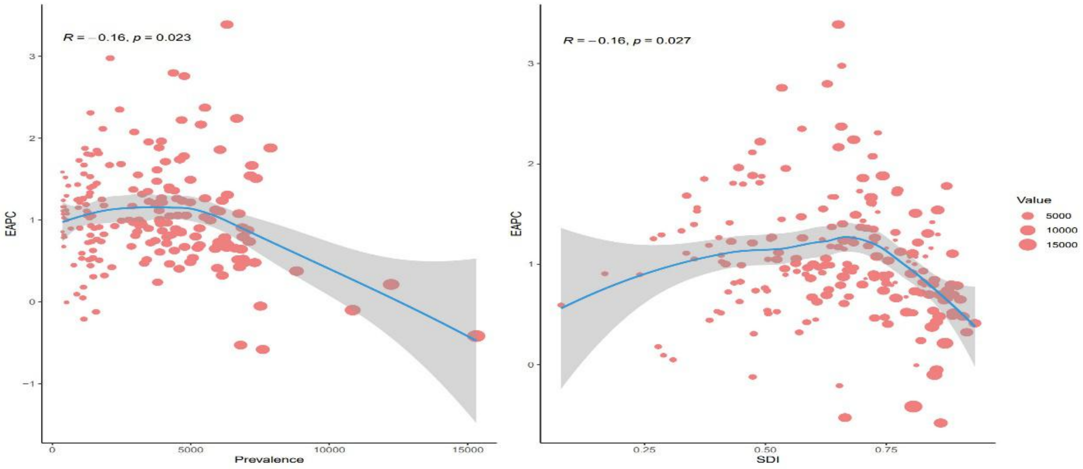

B

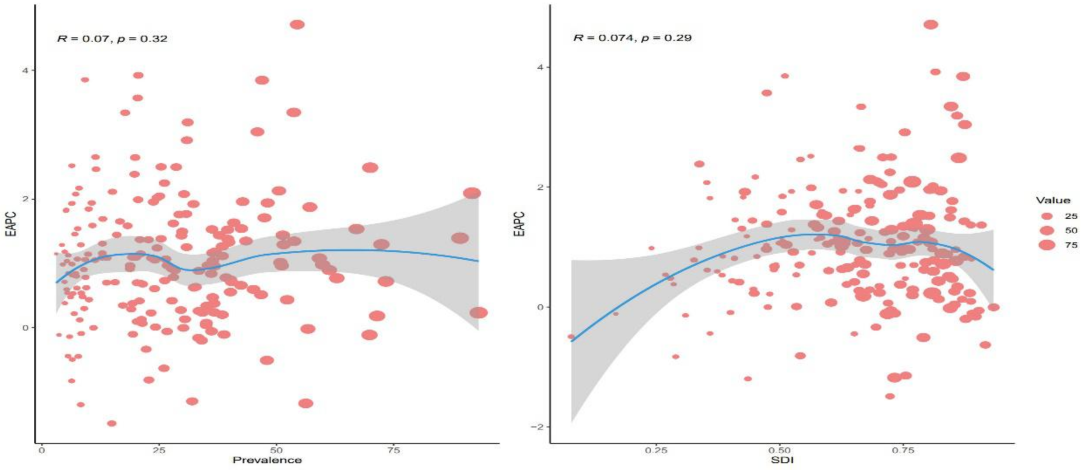

C

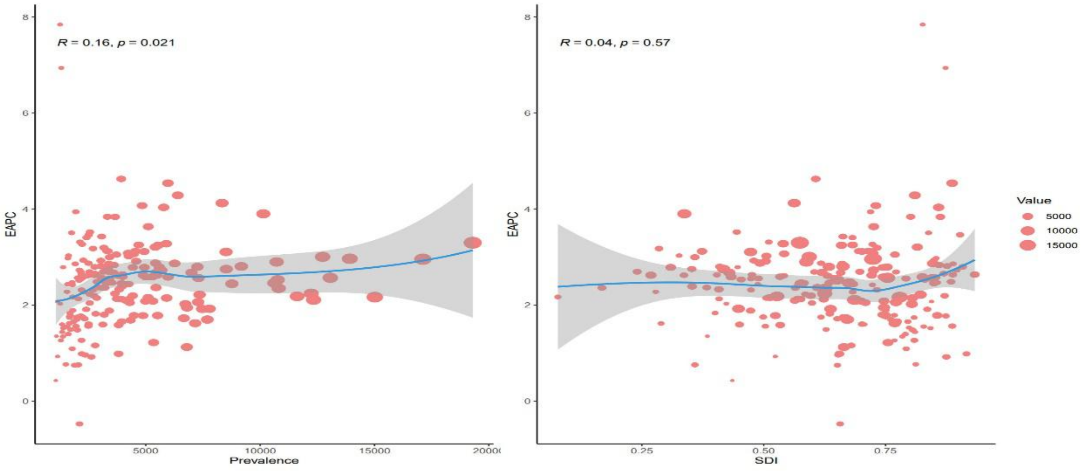

Fig. S8 The EAPC of ASPR for PCOS,uterine cancer,T2DM in 204 countries and territories by SDI. ASPR age- standardized prevalence rate, SDI sociodemographic index, A PCOS Polycystic ovary syndrome , B Uterine cancer ,C T2DM type 2 diabetes mellitus

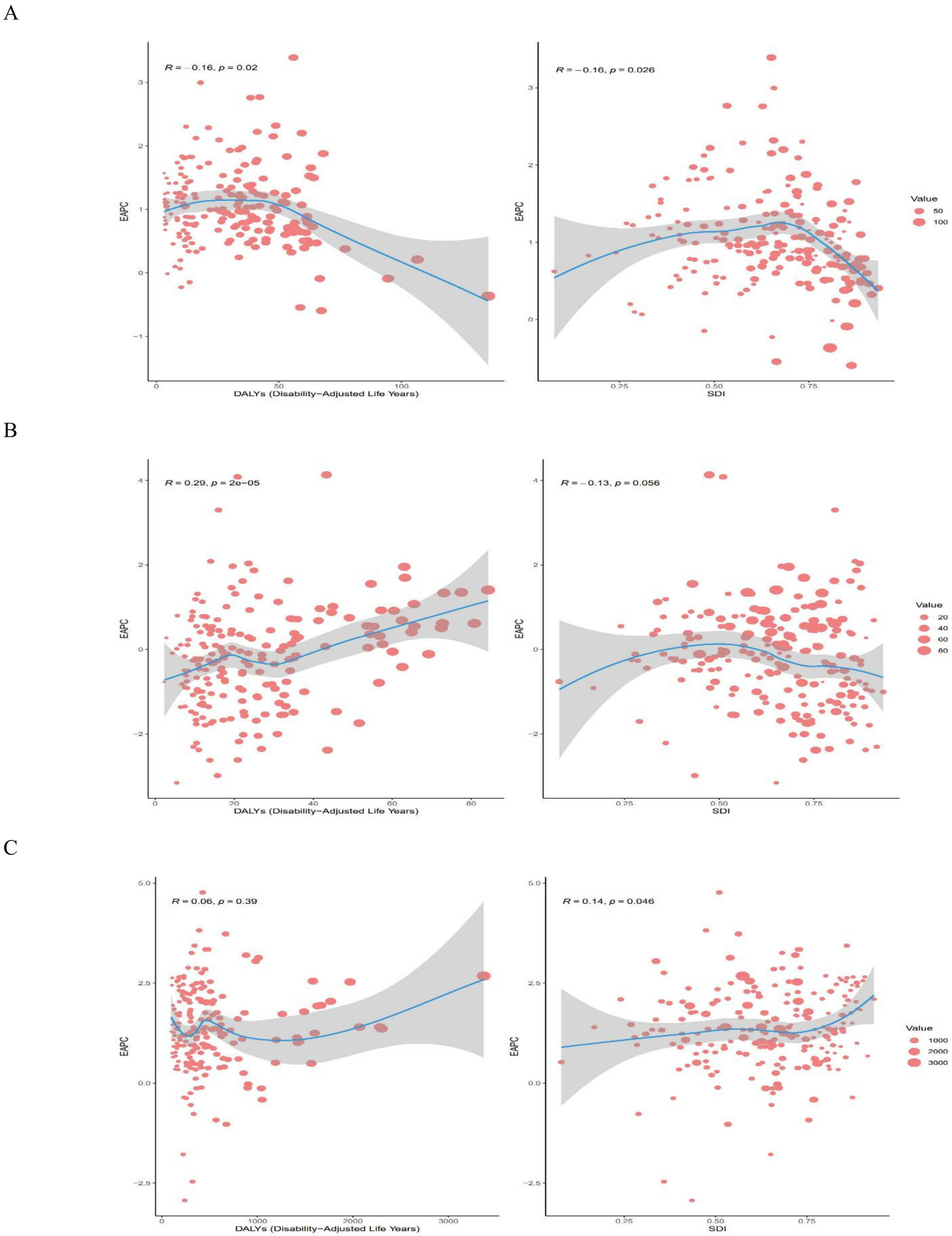

Fig. S9 The EAPC of ASDR for PCOS,uterine cancer,T2DM in 204 countries and territories by SDI. ASDR age-standardized DALYs , SDI sociodemographic index, A PCOS Polycystic ovary syndrome , B Uterine cancer ,C T2DM type 2 diabetes mellitus

A

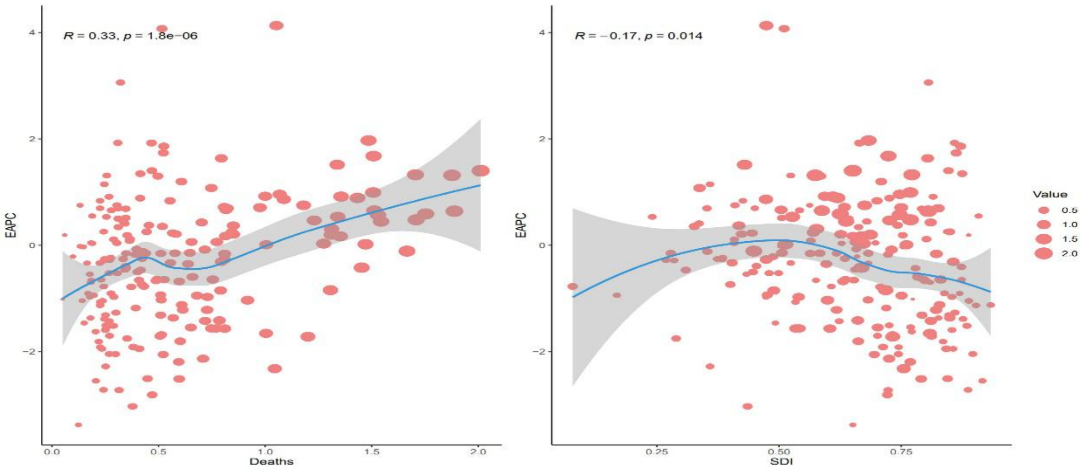

B

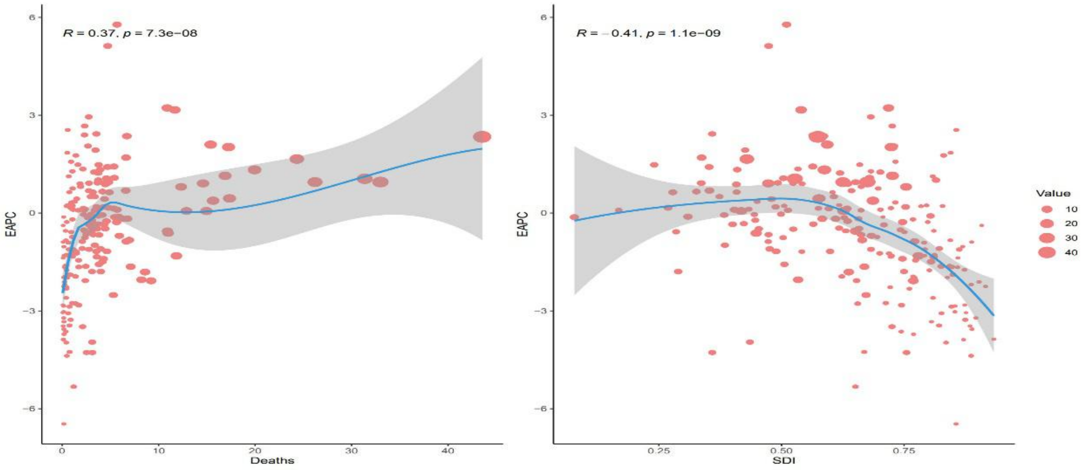

Fig. S10 The EAPC of ASMR for uterine cancer,T2DM in 204 countries and territories by SDI. ASMR age- standardized mortality , SDI sociodemographic index, A Uterine cancer,B T2DM type 2 diabetes mellitus

A r=0.4500, p=1.890e-11

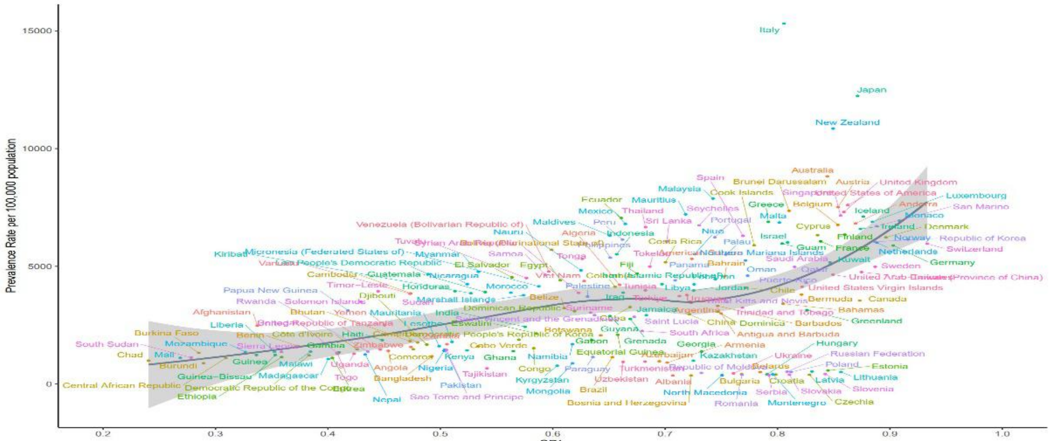

B r=0.6110, p=0.000e+00

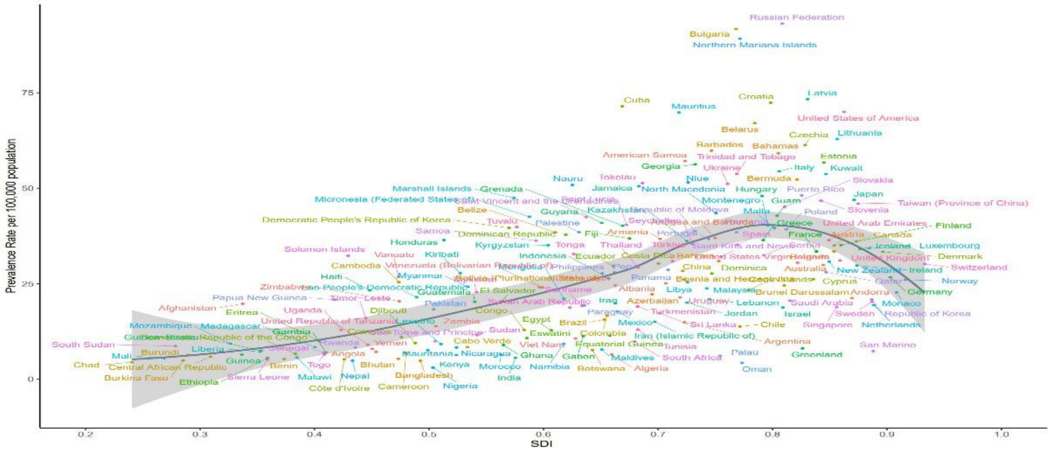

C r=-0.0722, p=3.045e-01

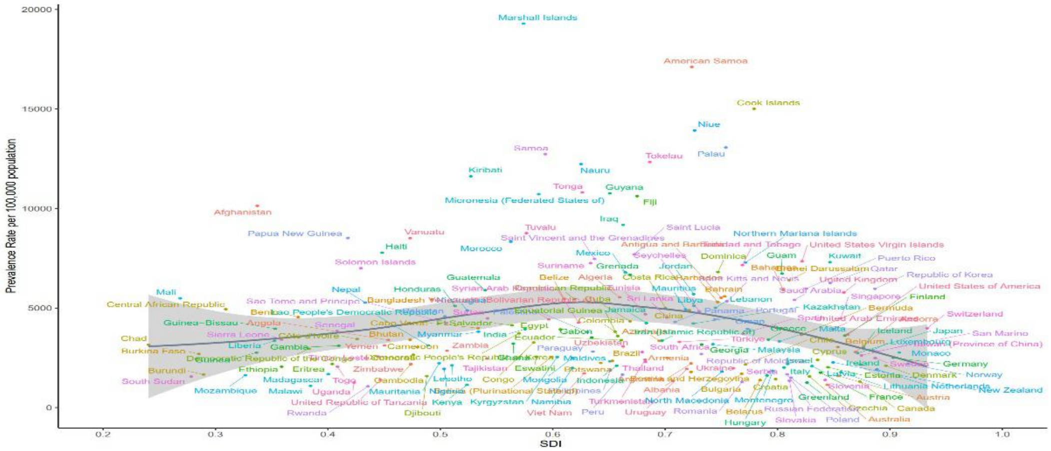

Fig. S11 ASPR for PCOS,uterine cancer,T2DM in 204 countries and territories by SDI in 2021. ASPR age-standardized prevalence rate, SDI sociodemographic index ,A PCOS Polycystic ovary syndrome , B Uterine cancer ,C T2DM type 2 diabetes mellitus

A r=0.4547, p=9.909e-12

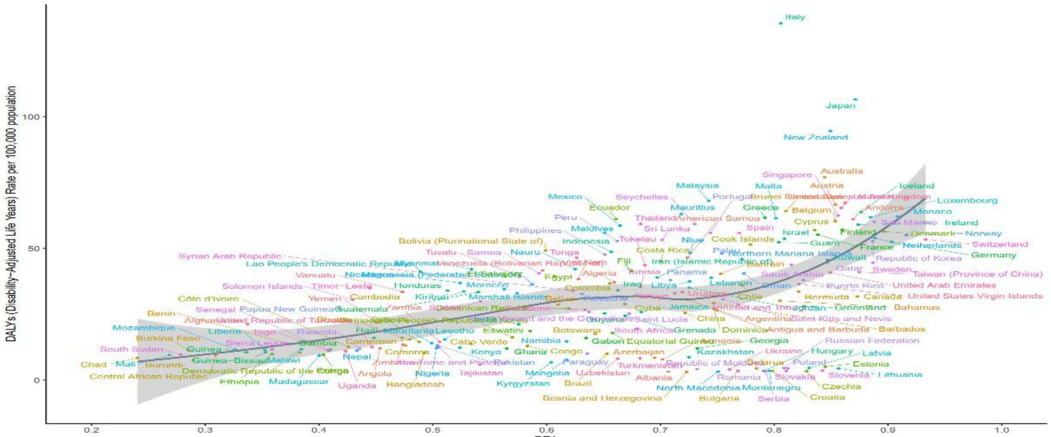

B r=-0.0121, p=8.638e-01

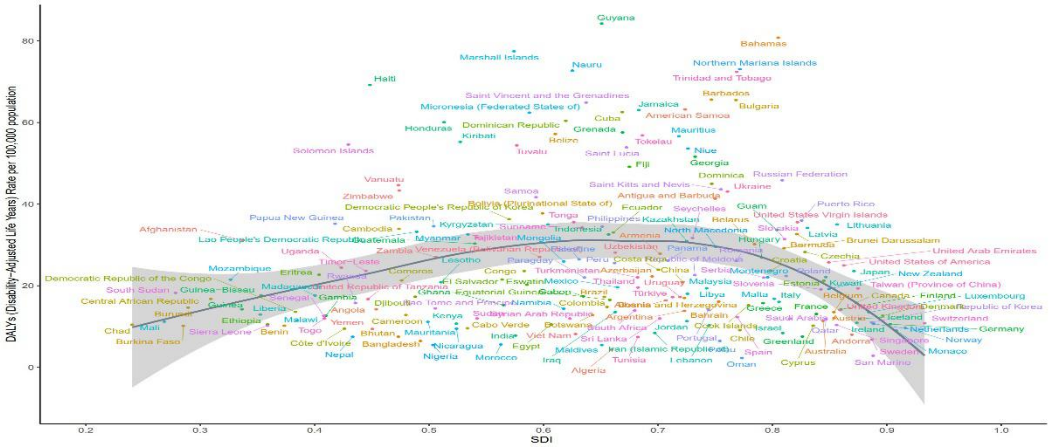

C r=-0.4315, p=1.710e-10

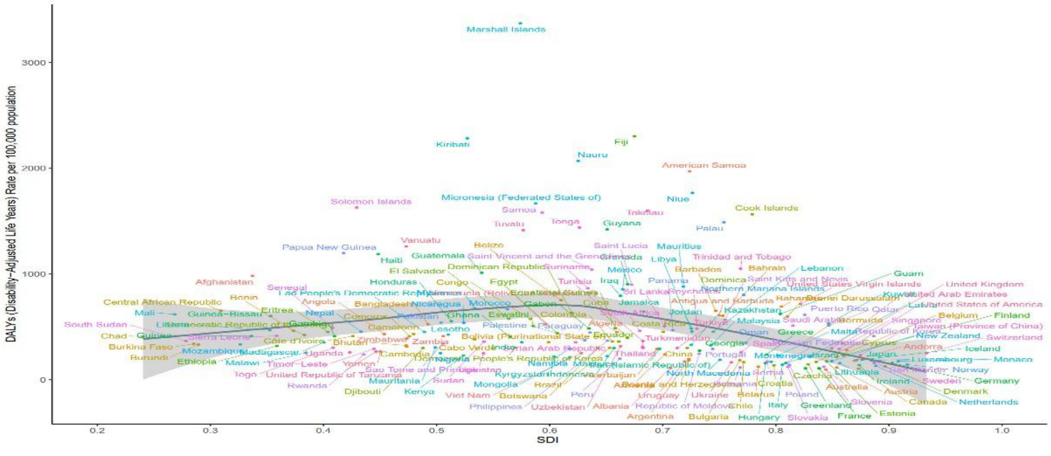

Fig. S12 ASDR for PCOS,uterine cancer,T2DM in 204 countries and territories by SDI in 2021. ASDR age-standardized DALYs , SDI sociodemographic index, A PCOS Polycystic ovary syndrome , B Uterine cancer ,C T2DM type 2 diabetes mellitus

A r=-0.0723, p=3.038e-01

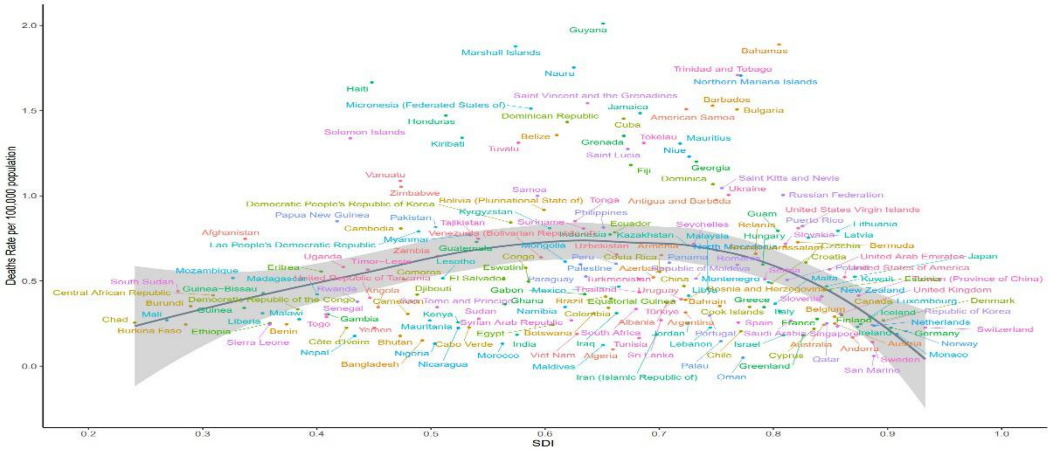

B r=-0.6463, p=0.000e+00

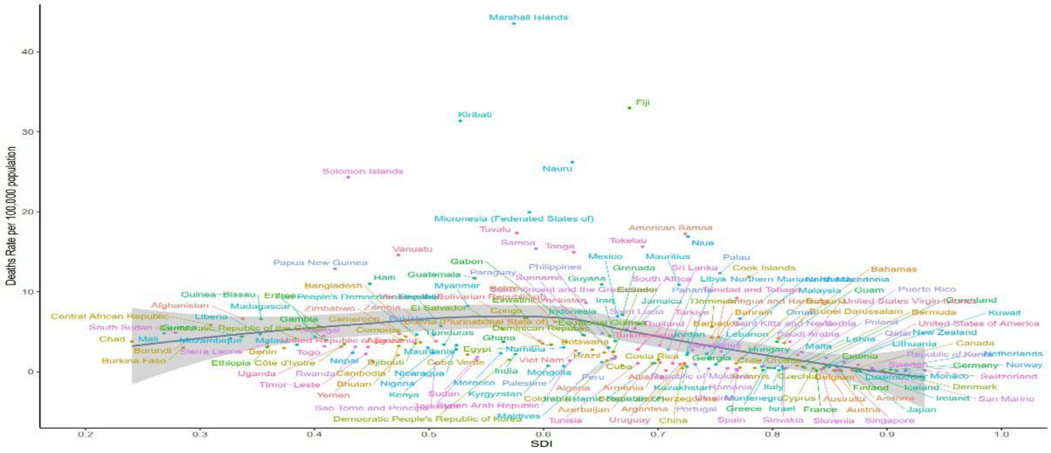

Fig. S13 ASMR for uterine cancer,T2DM in 204 countries and territories by SDI in 2021. ASMR age-standardized mortality , SDI sociodemographic index, A Uterine cancer ,B T2DM type 2 diabetes mellitus

A

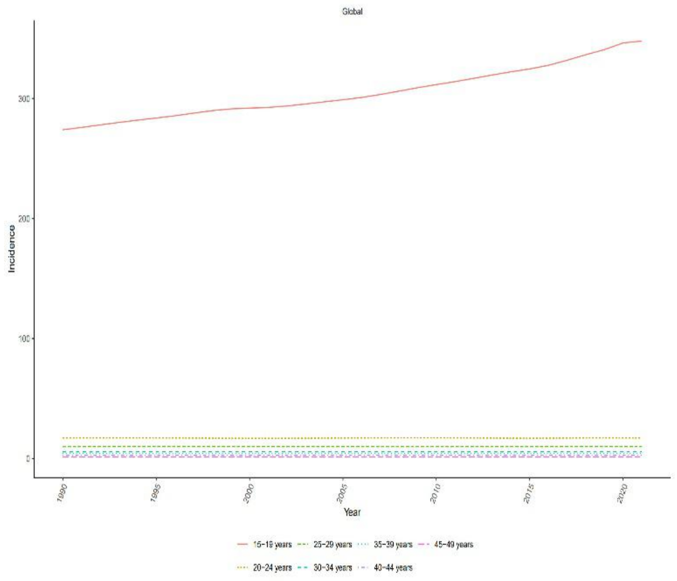

B

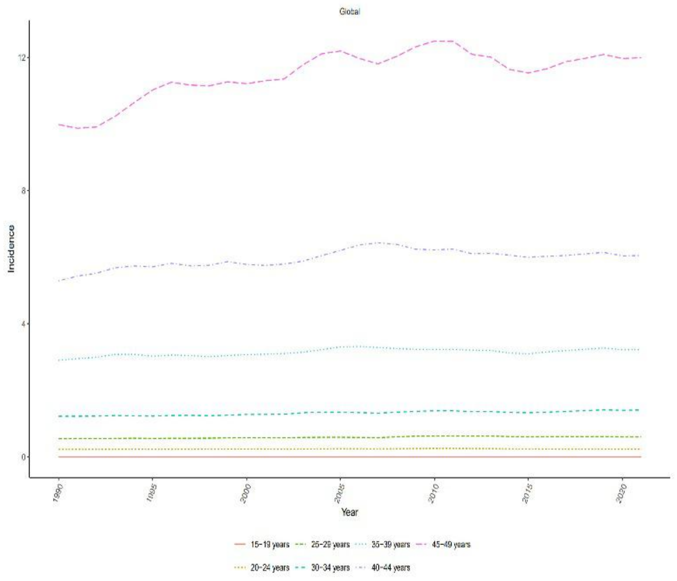

C

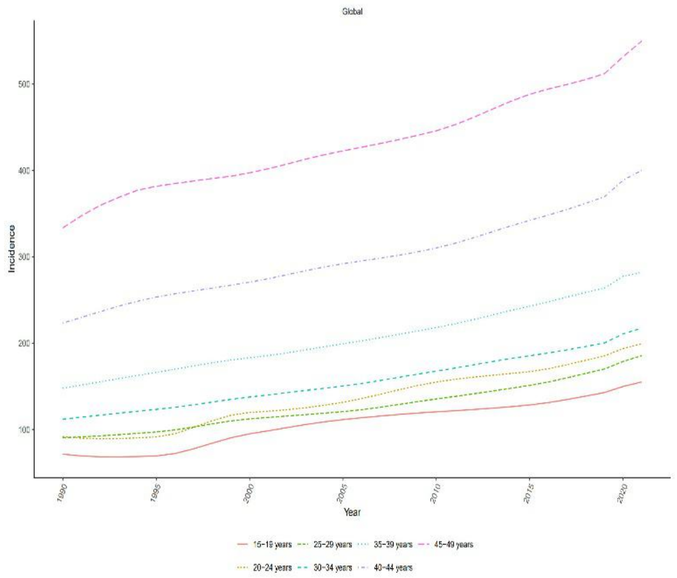

Fig. S14 Global ASIR of PCOS,uterine cancer and T2DM by age in 2021. A PCOS Polycystic ovary syndrome , B Uterine cancer ,C T2DM type 2 diabetes mellitus

A

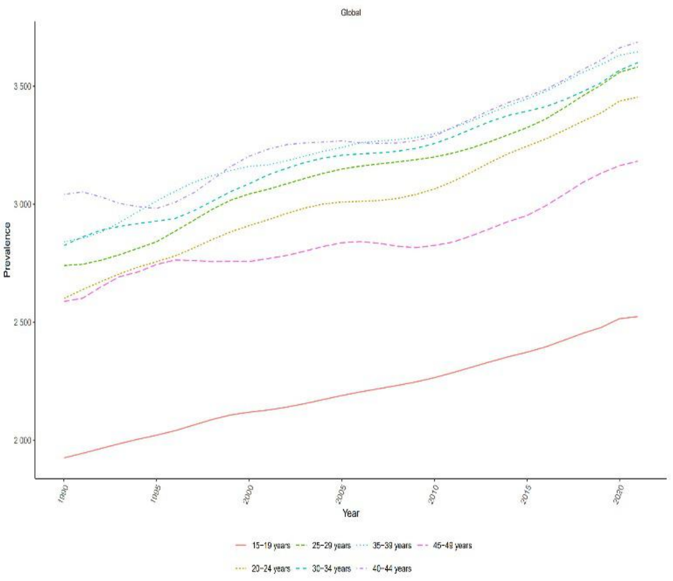

B

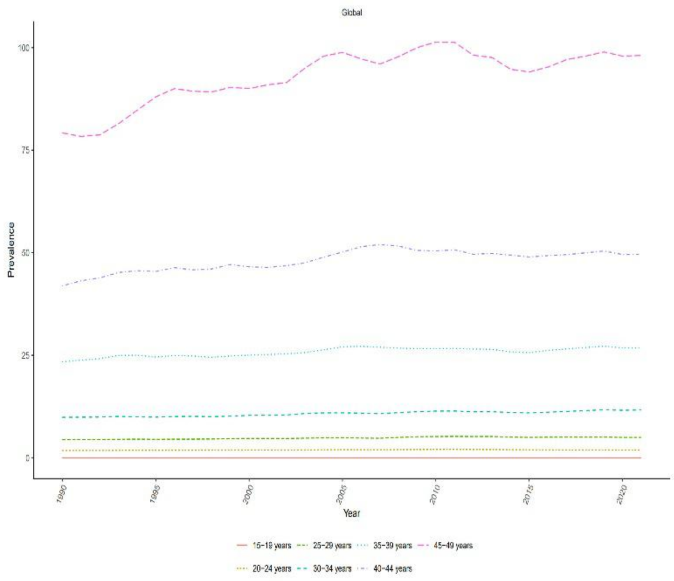

C

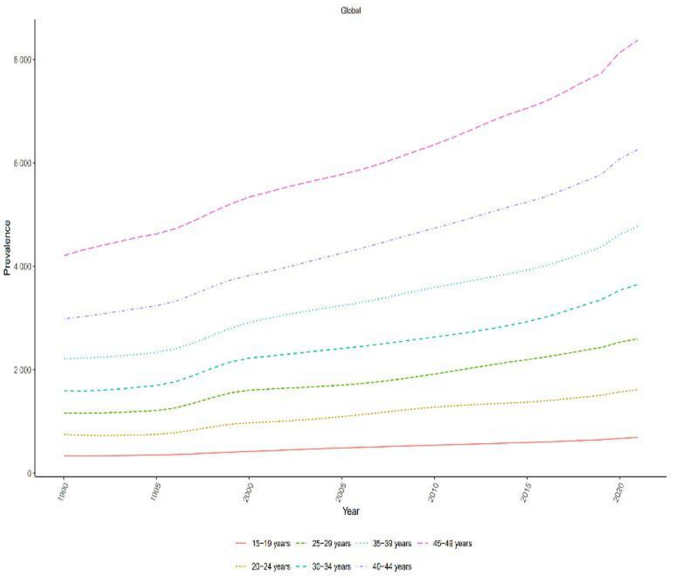

Fig. S15 Global ASPR of PCOS,uterine cancer and T2DM by age in 2021. A PCOS Polycystic ovary syndrome , B Uterine cancer ,C T2DM type 2 diabetes mellitus

A

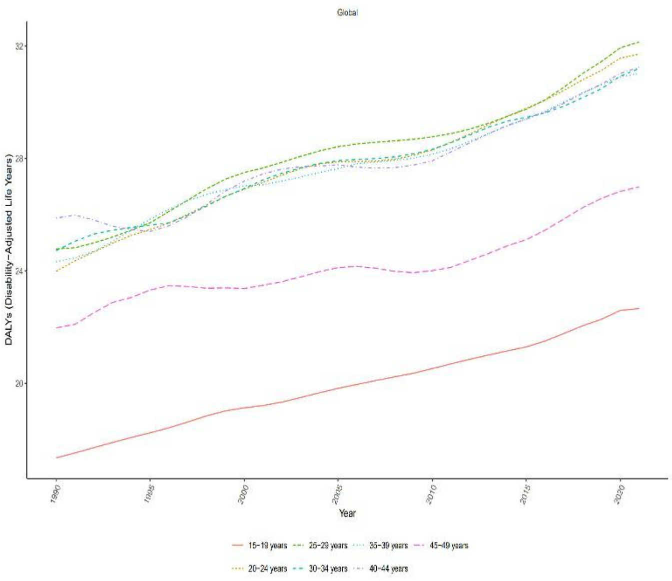

B

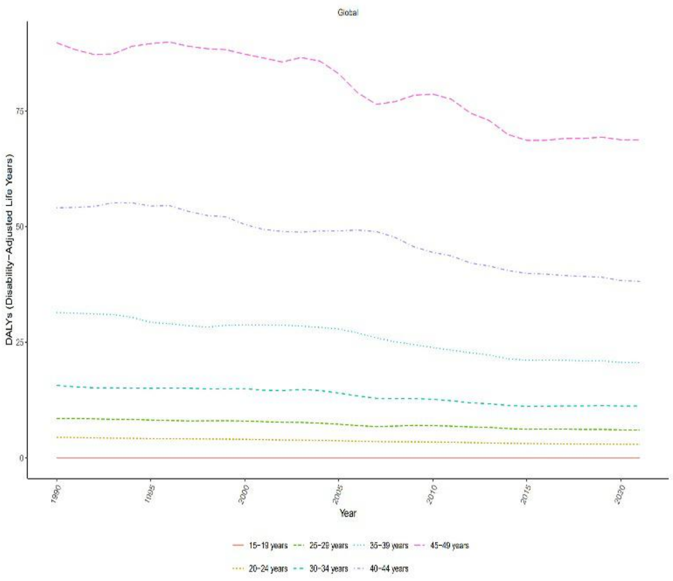

C

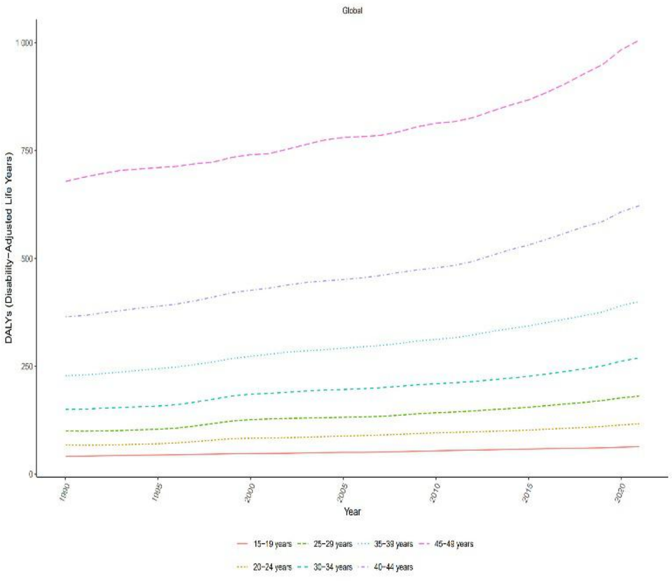

Fig. S16 Global ASDR of PCOS,uterine cancer and T2DM by age in 2021. A PCOS Polycystic ovary syndrome , B Uterine cancer ,C T2DM type 2 diabetes mellitus

A

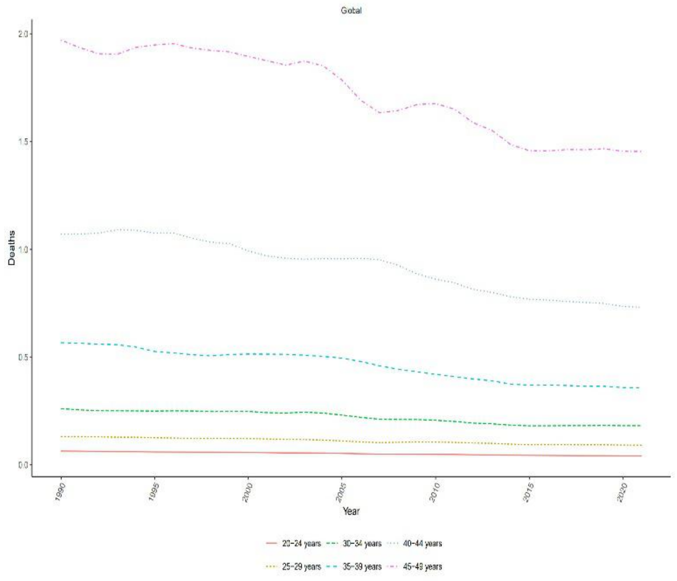

B

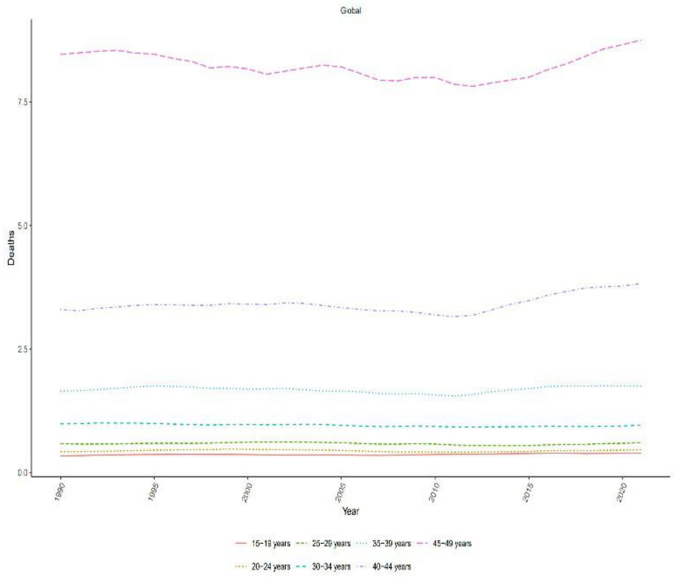

Fig. S17 Global ASMR of uterine cancer and T2DM by age in 2021. A Uterine cancer ,B T2DM type 2 diabetes mellitus
